# Dactylospenes A–E, Sesterterpenes from the Marine Sponge *Dactylospongia elegans*

**DOI:** 10.3390/md18100491

**Published:** 2020-09-25

**Authors:** Hao-Bing Yu, Bin-Bin Gu, Arihiro Iwasaki, Wen-Li Jiang, Andrew Ecker, Shu-Ping Wang, Fan Yang, Hou-Wen Lin

**Affiliations:** 1 Department of Marine Biomedicine and Polar Medicine, Naval Medical Center of PLA, Second Military Medical University, Shanghai 200433, China; yuhaobing1986@126.com; 2 Research Center for Marine Drugs, State Key Laboratory of Oncogenes and Related Genes, School of Medicine, Shanghai Jiao Tong University, Shanghai 200127, China; 18521018251@163.com (B.-B.G.); shupingwang2007@163.com (S.-P.W.); 3 Department of Chemistry, Faculty of Science and Technology, Keio University, 3-14-1 Hiyoshi, Kohoku-ku, Yokohama, Kanagawa 223-8522, Japan; a.iwasaki@chem.keio.ac.jp; 4 Department of Biochemistry and Molecular Biology, College of Basic Medical Sciences, Second Military Medical University, Shanghai 200433, China; jwlsally@163.com; 5 Center for Marine Biotechnology and Biomedicine, Scripps Institution of Oceanography, University of California, San Diego, CA 92093, USA; aecker@ucsd.edu

**Keywords:** sesterterpenes, hydroxybutenolide, *Dactylospongia*, cytotoxicity, anti-inflammatory

## Abstract

Chemical investigation on a marine sponge, *Dactylospongia elegans*, yielded five new γ-oxygenated butenolide sesterterpene derivatives, dactylospenes A–E (**1**–**5**), as well as two known biosynthetically related compounds, luffariellolide (**6**) and furospinosulin B (**7**). The structures of these compounds were elucidated on the basis of their spectroscopic data, experimental and calculated electronic circular dichroism (ECD) analysis, as well as comparison of the NMR data with those of known analogs. These metabolites are the first γ-oxygenated butenolide sesterterpenes to be reported from this genus. These compounds were evaluated in antimicrobial, anti-inflammatory, and cytotoxic assays. Only compounds **1**, **3**, and **6** exhibited moderate cytotoxicity against DU145, SW1990, Huh7, and PANC-1 cancer cell lines with IC_50_ values in the range of 2.11–13.35 μM. Furthermore, compound **2**, without cytotoxicity, exhibited significant inhibitory effects (inhibitory rate 77.5%) on nitric oxide production induced by lipopolysaccharide at 10 μM.

## 1. Introduction

Sesterterpenoids, mainly found in marine organisms, are a rare class of terpene that are structurally diverse and have a wide spectrum of biological activities [1,2]. Marine sponges represent an abundant source of bioactive sesterterpenoids [3,4]. A literature survey revealed that only three sesterterpenoids and no γ-oxygenated butenolide sesterterpenes or related derivatives have been isolated from sponges of the genus *Dactylospongia* [5,6]. Aside from a few sesterterpenoids, sponges of the genus *Dactylospongia*, in particular *D. elegans*, have been widely investigated as a rich source of sesquiterpene quinones/quinols, sesquiterpene acids, sesterterpene lactones, macrolides, and steroids [7]. These metabolites showed a spectrum of bioactivities, such as cytotoxic [8], anti-inflammatory [9], antibacterial [10], and protein kinase inhibitory activities [11].

**Figure 1:**
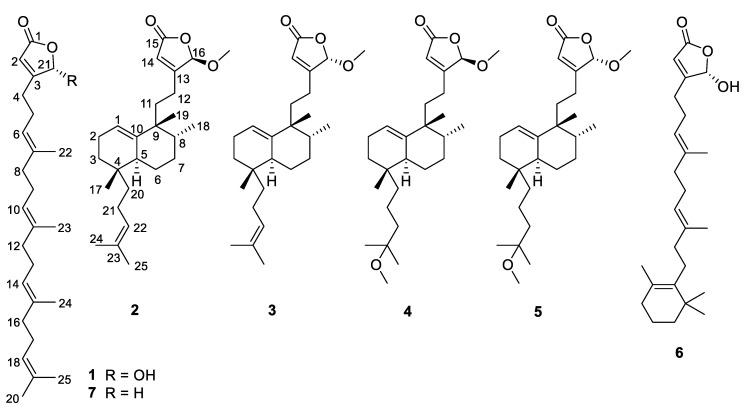
Chemical structures of compounds **1**–**7**.

As part of the ongoing bioactive natural product discovery from the organic extract of a marine sponge *D. elegans*, one fraction showed different LC-DAD-MS profiles to those of previously reported sesquiterpene quinones/hydroquinones based upon LC-MS analysis [6]. Further chemical investigation of this fraction led to the isolation of γ-oxygenated butenolide sesterterpene derivatives, dactylospenes A–E (**1**–**5**), and two known compounds (**6**–**7**), shown in Figure 1. Herein, we report the details of the isolation, structure elucidation, and biological activity evaluation of these metabolites.

## 2. Results

**Figure 2:**
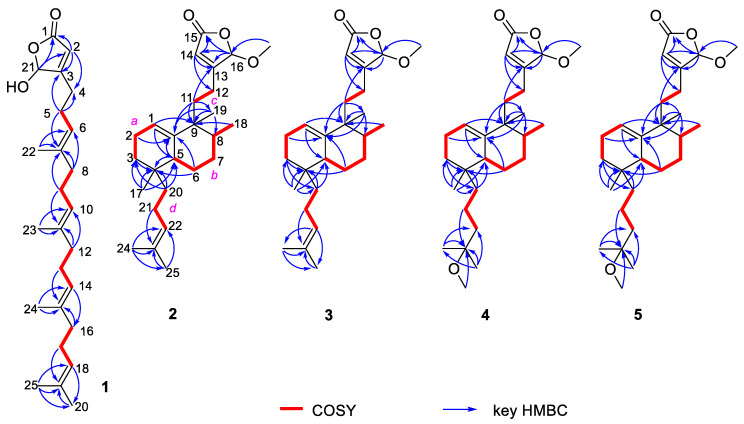
COSY and key HMBC correlations for compounds **1**–**5**.

**Figure 3:**
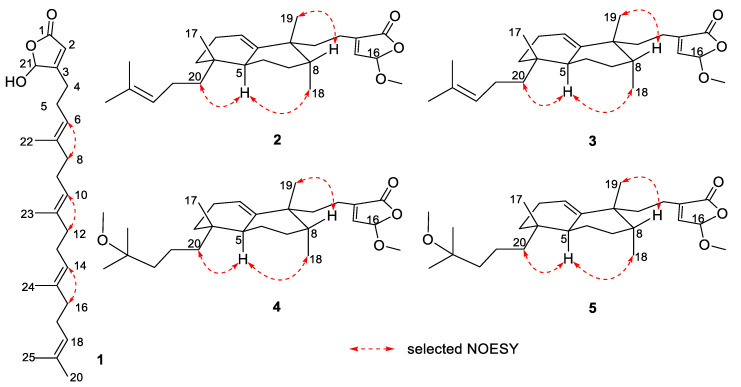
Selected NOESY correlations for compounds **1**–**5**.

**Figure 4:**
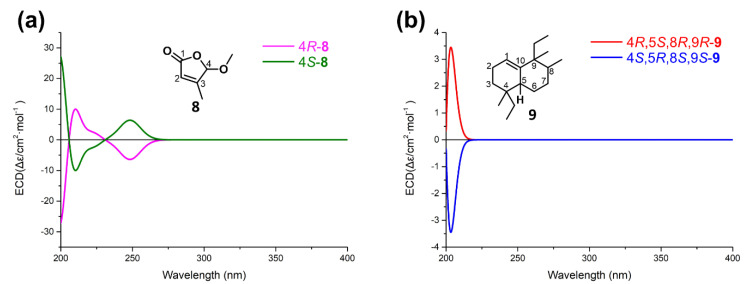
Calculated electronic circular dichroism (ECD) spectra of the simplified models **8** (**a**) and **9** (**b**).

Dactylospene A (**1**) was obtained as a light red oil, and its molecular formula was determined as C_25_H_38_O_3_ according to the HRESIMS peak at *m/z* 404.3166 [M + NH_4_]^+^, requiring seven degrees of unsaturation. The IR spectrum showed absorption bands for hydroxy (3342 cm^−1^) and ester carbonyl (1760 cm^−1^) groups. Comparison of the ^1^H and ^13^C NMR data of **1** (Table 1) with those of the known compound **7** showed that they shared a linear sesterterpene skeleton [12], which was further confirmed by the HMBC and COSY correlations shown in Figure 2. The observation of a downfield shift in the C-21, from δ_C_ 73.1 in **7** to δ_C_ 99.1 in **1**, confirmed that the hydroxyl group connected at C-21. The relative configuration of the double bonds in **1** was inferred to be the same as compound **7** and further established by the NOESY experiments (Figure 3). Strong NOESY correlations of overlapped proton signals at δ_H_ 2.00 and δ_H_ 5.11 suggested that these three groups of double-bonds Δ^6,7^, Δ^10,11^, and Δ^14,15^ were all *E*-geometry. Furthermore, the absolute stereochemistry of C-21 can be determined by following the determination method of *O*,*O*-dimethyllingshuiolide A [13]. The characteristic positive Cotton effect at 253 nm in the CD spectrum of **1** was virtually identical to that of the simplified models (detailed in the Supplementary Information S1), (*S*)-5-hydroxy-4-methylfuran-2(5*H*)-one (4*S*-**8**) (Figure 4). Consequently, absolute configuration of **1** was unassigned as 21*S*.

Dactylospene B (**2**) was also purified as a light red oil and exhibited a pseudomolecular ion [M + NH_4_]^+^ peak in the positive HRESIMS at *m/z* 418.3308, consistent with the molecular formula C_26_H_40_O_3_, which was supported by the ^1^H and ^13^C NMR data. The molecular formula indicated seven degrees of unsaturation. The IR spectrum of **2** showed strong absorption at 3465 and 1766 cm^−1^, assignable to hydroxy and ester carbonyl functionalities, respectively. The ^13^C NMR and DEPT spectra (Table 2) of **2** revealed 26 carbon signals, including one carbonyl carbon (δ_C_ 170.3), three sp^2^ quaternary carbons (δ_C_ 168.6, 144.4, and 130.9), three sp^2^ methine carbons (δ_C_ 124.6, 117.9, and 116.9), one sp^3^ oxymethine (δ_C_ 103.9), one methoxy (δ_C_ 56.1), two sp^3^ quaternary carbons (δ_C_ 42.0 and 33.2), two methine carbons (δ_C_ 43.9 and 41.8), eight methylene carbons (δ_C_ 38.6, 30.8, 29.4, 28.7, 27.5, 22.4, 21.9, and 21.7), and five methyl carbons (δ_C_ 25.2, 23.5, 22.4, 17.2, and 15.9), which accounted for four degrees of unsaturation. The remaining three degrees of unsaturation were caused by the presence of three rings in the molecule.

Interpretation of the 2D NMR data, including COSY, HMQC, and HMBC spectra, led to the construction of the planar structure of **2**. The COSY spectrum of **2** suggested the presence of four individual spin systems: C-1−C-2−C-3 (*a*), C-5−C-6−C-7−C-8−C-18 (*b*), C-11−C-12 (*c*), and C-20−C-21−C-22 (*d*), which were accomplished with the assistance of the HMBC experiment (Figure 1). HMBC correlations from H_3_-24 to C-22, C-23, and C-25, from H_3_-25 to C-22, C-23, and C-24, and from H_2_-22 to C-24 and C-25 determined the existence of dimethylallyl moiety (C20-C25). Moreover, HMBC correlations from H_2_-3 in fragment a, and H-5 and H_2_-6 in fragment b to the same carbon C-4, as well as HMBC correlations from H_3_-17 to C-3, C-4, and C-5, allowed the linkage of fragments *a* and *b* via C-4 and the assignment of the methyl group CH_3_-17 at C-4. The dimethylhomoallyl group was attached to C-4 based on the HMBC correlations from H_2_-20 to C-3, C-4, and C-5. Further HMBC correlations from H_3_-18 to C-9, and from H_3_-19 to C-8, C-9, and C-10, revealed the connectivity of C-8 and C-10 through C-9 and placed the methyl group H_3_-19 at C-9 as well. Another two groups of HMBC cross-peaks from H-1 to C-5 and C-9, from H_2_-2, H-5 and H-6b to C-10, supported the linkage of C-1 and C-5 via C-10. Moreover, a suite of resonances at δ_C_ 170.3 (C-15), 168.6 (C-13), 116.9 (CH-14), 103.9 (CH-16), and 56.1 (16-OCH_3_), could be assigned to an α,β-unsaturated-γ-methoxy-γ-lactone moiety, which was further supported by HMBC correlations from H-16 to C-13, C-14, and C-15, from H-14 to C-13, C-15, and C-16, and from 16-OCH_3_ to C-16. This moiety was further linked to C-9 through fragment C-11–C-13, based on the HMBC correlations from H-14 to C-12 and from H-11 to C-9, C-10, and C-13. Thus, the planar structure of **2** was determined as depicted.

A NOESY experiment was also performed to determine the relative configuration of **2**. The NOESY cross-peaks of H-5/H-20a and H-5/H_3_-18 indicated the cofacial orientation of these protons and methyl group, whereas the NOESY correlations of H-8/H_3_-19 indicated that these protons were oriented in the other direction. Therefore, we established the relative stereochemistry of **2** as 4R*,5S*,8R*,9R*.

**Figure 5:**
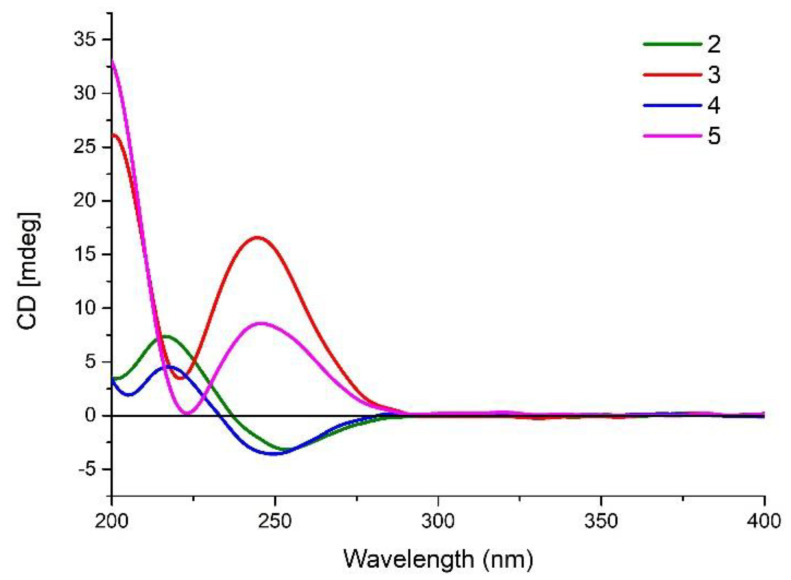
Experimental CD spectrum (MeCN) of **2**–**5**.

The molecular formula for dactylospene C (**3**) was also deduced as C_26_H_40_O_3_ by HRESIMS. Analysis of the 1D and 2D NMR data (Table 2) for **3** and **2** showed that they shared the same planar structure. The similar NOESY correlations of H-5/H-20a, H-5/H_3_-18, and H-8/H_3_-19 revealed the same relative configurations of **3** as those of **2** at C-4, C-5, C-8, and C-9. However, the different but not mirrored CD spectra (Figure 5) and specific rotation values ($[\alpha {]}_{D}^{25}$ 13.9 for **2**, $[\alpha {]}_{D}^{25}$ 56.2 for **3**) of **2** and **3** indicated that these two compounds are a pair of diastereomers.

To establish the absolute configuration of **2** and **3**, we compared the ECD spectra of **2** and **3** with calculated ECD spectra of simplified models, 4*R*-**8** and 4*R*,5*S*,8*R*,9*R*-**9** (Figure 4). The negative cotton effect around 250 nm in **2** clearly indicated that the absolute configuration at C-16 is *R*. Meanwhile, the positive cotton effect around 250 nm in **3** allowed us to conclude that the absolute configuration at C-16 in **3** is *S*. Then, we determined the absolute configuration of the decaline substructure. As described above, **2** and **3** are a pair of diastereomers, and the only difference is the absolute configuration at C-16. Therefore, **2** and **3** have the same absolute configuration in the decaline moiety. According to the calculated ECD spectra of **9**, if the compound has 4*R*,5*S*,8*R*,9*R* configuration, it shows a positive cotton effect around 205 nm. In the experimental ECD spectra, the positive cotton effect around 205 nm in **3** is more emphasized than the negative cotton effect around 205 nm in 2. This indicated that the decaline moiety in **2** and **3** has positive cotton effect around 205 nm. Therefore, we clarified the absolute configuration at C-4, C-5, C-8, and C-9 in **2** and **3** to be 4*R*,5*S*,8*R*,9*R*.

Dactylospene D (**4**) was isolated as a yellow oil and assigned the molecular formula of C_27_H_44_O_4_, based on HRESIMS data for the [M + NH_4_]^+^ ion at *m/z* 450.3581, which is consistent with six degrees of unsaturation. The ^1^H and ^13^C NMR signal patterns (Table 3) suggested the same structure core as compounds **2** and **3** with a α,β-unsaturated-γ-methoxy-γ-lactone moiety linked to the *epi*-halimane-type diterpenoid. Key HMBC and COSY correlations shown in Figure 2 confirmed this proposed structure. Through analysis of the remaining signals in the ^13^C NMR, two methyls (δ_C_ 24.9 and 25.0), three sp^3^ methylenes (δ_C_ 41.1, 39.8, and 17.8), one methoxy (δ_C_ 49.1), and one sp^3^ oxygenated quaternary carbon (δ_C_ 74.6) were deduced. The attachment at C-4 was a 2-methoxy-2-methylpentane unit, instead of the dimethylhomoallyl moiety in **2** and **3**, supported by the COSY correlations of H-20a/H-21a and H-21b/H_2_-22, in addition to the HMBC correlations from H-20a to C-3, from H_3_-24 and H_3_-25 to C-22 and C-23, and from 23-OCH_3_ to C-23. Moreover, NOESY correlations of H-5/H-20a, H-5/H_3_-18, and H-8/H_3_-19 revealed the relative configurations of **4**, which were the same as compounds **2** and **3**. Finally, the similarity of their CD spectra between **2** and **4** suggested that compound **4** shared the same absolute configurations as those of **2** (Figure 5).

Dactylospene E (**5**) was also obtained as a light yellow oil. The molecular formula of C_27_H_44_O_4_ was deduced from its HRESIMS data (*m/z* 450.3578 [M + NH_4_]^+^). Compound **5** showed nearly the same chemical shifts as those of compound **4**. Correlations from the 2D NMR spectra confirmed the same planar structure between **5** and **4** and the same configuration of *epi*-halimane core in **5** and **4**. A comparison with CD spectra and specific rotation values obtained for **5** and **3** unambiguously assigned absolute configuration as 4*R*,5*S*,8*R*,9*R*,16*R*.

In addition to the five new compounds **1**–**5**, two known compounds, identified as luffariellolide (**6**) and furospinosulin B (**7**), were also isolated as metabolites of *D. elegans*. These compounds were identified by comparing their spectral data with the spectroscopic data reported in the corresponding literature [12,14]. Moreover, the C-25 stereocenter in the γ-oxygenated butenolide unit could be assigned as an S configuration by comparison the specific rotation data of **6** ($[\alpha {]}_{D}^{25}$ − 35.3, MeCN, *c* 1.0) with that of **1**. Compounds **4** and **5** could possibly be formed by reaction with MeOH from compounds **2** and **3** during the isolation. However, when compounds **2** and **3** were stirred with silica and ODS in MeOH for 48 h, neither **4** nor **5** were detected by HPLC-UV analysis.

All the isolated compounds were tested for antimicrobial activity against two strains of hospital-acquired, methicillin-resistant *Staphylococcus aureus* (MRSA H0556 and MRSAH0117) and cytotoxic activity against DU145, SW1990, Huh7, and PANC-1 cancer cell lines. Unfortunately, the compounds tested exhibited no activity against the above strains. Only compounds **1**, **3**, and **6** exhibited moderate cytotoxicity against the above four cancer cell lines, with IC_50_ values in the range of 2.11–13.35 μM, while the other isolates were inactive (IC_50_ values > 50 μM) (Table 4). Moreover, compounds **1**–**5** were subjected to an evaluation of their anti-inflammatory activity. Compound **2** exhibited significantly greater inhibitory effects than **3** (inhibitory rate 77.5% for 2 and 30.4% for 3) on nitric oxide (NO) production induced by lipopolysaccharide (LPS) treatment of RAW 264.7 cells at 10 μM. In addition, the proliferation rate of RAW 264.7 cells was 151.2% with the treatment of **2** at 10 μM, which indicated that the anti-inflammatory effect of **2** was not achieved by its cytotoxicity. The biological evaluation indicated that *R*-γ-methoxy butenolide moiety positively affected the activity.

## 3. Experimental Section

### 3.1. General Experimental Procedures

UV, IR (KBr), and CD spectra were obtained on UV-8000 spectrophotometer (Shanghai Metash instruments Co., Shanghai, China), Jasco FTIR-400 spectrometer (Jasco Inc., Tokyo, Japan), and Jasco J-715 spectropolarimeter (Jasco Inc., Tokyo, Japan) in MeCN, respectively. Optical rotations were recorded on a Perkin-Elmer model 341 polarimeter (Perkin-Elmer Inc., Waltham, MA, USA). 1D NMR and 2D NMR spectra were acquired at room temperature (rt) using Bruker AMX-400 and Bruker Avance III DRX-600 instruments (Bruker Biospin Corp., Billerica, MA, USA) with TMS as the internal standard. HRESIMS data were obtained with the positive ion mode on an Agilent 6210 LC/MSD TOF mass spectrometer (Agilent Technologies Inc. Lake Forest, CA, USA). Reversed-phase HPLC was performed on a YMC-Pack Pro C_18_ RS (5 μm) column (YMC Co. Ltd., Kyoto, Japan) using the Waters 1525 separation module (Waters Corp., Milford, MA, USA) with a Waters 2998 photodiode array (PDA) detector (Waters Corp., Milford, MA, USA). Silica gel (200–300 mesh, Qingdao Ocean Chemical Co., Qingdao, China), Sephadex LH-20 (18–110 μm, Pharmacia Co., Piscataway, NJ, USA), and ODS (50 μm, YMC Co. Ltd., Kyoto, Japan) were used for column chromatography.

### 3.2. Animal Material

The marine sponge was collected off Yongxing Island in the South China Sea in March 2018 and identified as *D. elegans* by Professor Hou-Wen Lin. The sample of *D. elegans* (YC-3-2018) is deposited at the Department of Biochemistry and Molecular Biology, College of Basic Medical Sciences, Second Military Medical University.

### 3.3. Fermentation, Extraction, and Isolation

The air-dried sponge (0.3 kg, dry weight) was powdered and extracted by 95% aqueous EtOH at rt. The combined extracts were concentrated under vacuum to give the crude extract (9.3 g), which was subjected to vacuum liquid chromatography on silica gel by gradient elution using CH_2_Cl_2_/MeOH (100:0 to 0:100, *v:v*) as solvents to give seven fractions (A–G). Fraction D (1.16 g) was further separated on an ODS (50 μm) column by stepwise gradient elution with MeOH/H_2_O (1:4, 2:3, 3:2, 4:1, 1:0) to afford 11 subfractions (D1–D11), and then subfractions D10 was subjected to column chromatography (CC) on Sephadex LH-20 with CH_2_Cl_2_/MeOH (1:1) as the eluting solvent to afford three subfractions (D10a–D10c). Subfraction D10b was purified by CC on Silica gel with *n*-hexane/acetone (15:1) as the eluting solvent to afford **2** (1.8 mg), **3** (1.6 mg), and subfraction D10b4, which was further purified by reversed-phase HPLC, eluting with 90% MeCN (2.0 mL/min), detected at 254 nm, to give **4** (t_R_ = 23.3 min, 1.4 mg) and **5** (t_R_ = 24.1 min, 1.6 mg). Fraction E (1.02 g) was further separated on an ODS (50 μm) column followed by stepwise gradient elution with MeOH/H_2_O (3:2, 4:1, 1:0) to afford ten subfractions (E1–E10), and then subfractions E7 and E9 were both purified by reversed-phase HPLC, eluting with 70% MeCN (2.0 mL/min), detected at 220 nm, to give **6** (1.2 mg, t_R_ = 13.3 min), **1** (13.1 mg, t_R_ = 15.2 min), and **7** (t_R_ = 20.5 min, 1.7 mg).

Dactylospene A (**1**): light red oil; $[\alpha {]}_{D}^{25}$ − 12.5 (*c* 0.13, MeOH); UV (MeOH) (log *ε*) *λ*_max_ 220 (4.29), 334 (2.13); IR (KBr) *ν*_max_ 3342, 2962, 2923, 2855, 2729, 1760, 1649, 1603, 1535, 1447, 1381, 1335, 1267, 1180, 1133, 1027, 952, 889, 805, 739, 599 cm^−1^; CD (MeCN) (∆ε) 216 (−0.3), 253 (+0.2); ^1^H and ^13^C NMR data, see Table 1; HRESIMS *m/z* 404.3166 [M + NH_4_]^+^ (calcd for C_25_H_42_NO_3_, 404.3159).

Dactylospene B (**2**): light red oil; $[\alpha {]}_{D}^{25}$ + 13.9 (*c* 0.20, MeOH); UV (MeOH) (log *ε*) *λ*_max_ 201 (4.47), 258 (4.02); IR (KBr) *ν*_max_ 3465,2959, 2925, 2858, 1795, 1766, 1652, 1454, 1375, 1309, 1120, 959, 897, 860 cm^−1^; CD (MeCN) (∆ε) 202 (+4.1), 216 (+8.9), 254 (−3.8); ^1^H and ^13^C NMR data, see Table 2; HRESIMS *m/z* 418.3308 [M + NH_4_]^+^ (calcd for C_26_H_44_NO_3_, 418.3316).

Dactylospene C (**3**): light red oil; $[\alpha {]}_{D}^{25}$ + 56.2 (c 0.27, MeOH); UV (MeOH) (log *ε*) *λ*_max_ 213 (4.64, 257 (4.36); IR (KBr) *ν*_max_ 3466, 2959, 2926, 2858, 1795, 1765, 1651, 1454, 1376, 1310, 1203, 1119, 955, 897, 863, 804, 736, 647 cm^−1^; CD (MeCN) (∆ε) 201 (+31.6), 221 (+4.2), 245 (+20.1); ^1^H and ^13^C NMR data, see Table 2; HRESIMS *m/z* 418.3314 [M + NH_4_]^+^ (calcd for C_26_H_44_NO_3_, 418.3316).

Dactylospene E (**4**): light yellow oil; $[\alpha {]}_{D}^{25}$ + 2.3 (*c* 0.12, MeOH); UV (MeOH) (log *ε*) *λ*_max_ 198 (4.36); IR (KBr) *ν*_max_ 3360, 2960, 2924, 2853, 1795, 1766, 1738, 1462, 1374, 1261, 1093, 1021, 800, 700 cm^–1^; CD (MeCN) (∆ε) 206 (+2.5), 217 (+5.9), 251 (−3.9); ^1^H and ^13^C NMR data, see Table 3; HRESIMS *m/z* 450.3581 [M + NH_4_]^+^ (calcd for C_27_H_48_NO_4_, 450.3578).

Dactylospene D (**5**): light yellow oil; $[\alpha {]}_{D}^{25}$ + 111.7 (*c* 0.10, MeOH); UV (MeOH) (log *ε*) *λ*_max_ 206 (4.58); IR (KBr) *ν*_max_ 2926, 2860, 1795, 1767, 1650, 1460, 1373, 1309, 1260, 1202, 1118, 955, 896, 861, 804, 735 cm^−1^; CD (MeCN) (∆ε) 200 (+43.1), 223 (+0.3), 246 (+11.2); ^1^H and ^13^C NMR data, see Table 3; HRESIMS *m/z* 450.3595 [M + NH_4_]^+^ (calcd for C_27_H_48_NO_4_, 450.3578).

### 3.4. ECD Calculations

Conformational searches for simplified models **8** and **9** were carried out via Macromodel 9.9.223 software (Schrödinger, LLC, Portland, OR, USA) using Merck Molecular Force Field (MMFF) applying a 21 kJ/mol energy window. Subsequently, the conformers with a Boltzmann population of over 1% were re-optimized at the B3LYP/6-31G(d) level with Gaussian 09 by employing the polarizable continuum model (PCM) in MeCN, which generated two conformers for model **8** and four conformers for model **9**. The theoretical calculations of ECD for simplified models **8** and **9** were calculated at the CAM-B3LYP/TZVP (PCM/MeCN) level. The ECD spectra were generated by the program SpecDis 1.6 applying a Gaussian band shape with the width of 0.35 eV, from dipole-length rotational strengths [15].

### 3.5. Biological Assays

The antimicrobial activities of compounds **1**–**7** against two strains of hospital-acquired, methicillin-resistant Staphylococcus aureus (MRSA H0556 and MRSAH0117) were evaluated according to Clinical and Laboratory Standards Institute (CLSI) guidelines [16,17], and chloromycetin was used as the positive control (MIC_90_ 2 μg/mL), while methicillin was used as the negative control (MIC_90_ 128 μg/mL). The cytotoxic activity of compounds **1**–**7** against DU145, SW1990, Huh7, and PANC-1 cell lines was performed by the Cell Counting Kit-8 (CCK-8) assay, as described before [18]. Each cancer cell line was treated with the indicated test compound at various concentrations, in triplicate, and cisplatin was used as a positive control. The anti-inflammatory assay of compounds **1**–**5** was measured using the Griess reagent following the reported method [19]. The cell viability assay of compounds **1**–**5** were evaluated by the CCK-8 assay, as above.

## 4. Conclusions

Investigation on the secondary metabolites from the marine sponge, *D. elegans*, led to the isolation and structure elucidation of a series of γ-hydroxybutenolide sesterterpene derivatives, dactylospenes A–E (**1**–**5**), together with two known biosynthetically related compounds **6**–**7**. From a biosynthetic perspective, compounds **2**–**5** may be generated from the possible precursor **1** by cyclization and methoxy-substitution reactions. These compounds were evaluated in antibacterial and cytotoxic activities. Only compounds **1**, **4**, and **6** exhibited moderate cytotoxicity against DU145, SW1990, Huh7, and PANC-1 cancer cell lines with IC_50_ values ranging from 2.11 to 13.35 μm. Compound **2** exhibited potent anti-inflammatory activity by inhibiting the production of NO in RAW264.7 cells activated by lipopolysaccharide, with an inhibitory rate of 77.5%. Interestingly, the anti-inflammatory activity of compound **2** was not achieved through cytotoxic activity, indicating that compound **2** deserves further study for its therapeutic potential to develop new anti-inflammatory drugs.

## Figures and Tables

**Table 1:** ^1^H (400 MHz) and ^13^C NMR (100 MHz) spectroscopic data of **1**.

Position	δ_C_	δ_H_, Mult. (*J* in Hz)	Position	δ_C_	δ_H_, Mult. (*J* in Hz)
**1**	171.6, C		**14**	124.2, CH	5.11, q (6.4) ^c^
**2**	117.6, CH	5.85, s	**15**	135.0, C	
**3**	169.5, C		**16**	39.6, CH_2_^a^	2.00, m ^c^
**4**	27.8, CH_2_	2.46, brd (30.8)	**17**	26.5, CH_2_^b^	2.06, m ^c^
**5**	25.2, CH_2_	2.32, m	**18**	123.9, CH	5.11, q (6.4) ^c^
**6**	121.9, CH	5.11, q (6.4) ^c^	**19**	131.3, C	
**7**	137.4, C		**20**	25.7, CH_3_	1.60, s ^c^
**8**	39.7, CH_2_^a^	2.00, m ^c^	**21**	99.1, CH	6.00, s
**9**	26.8, CH_2_^b^	2.06, m ^c^	**22**	17.7, CH_3_	1.63, s ^c^
**10**	124.4, CH	5.11, q (6.4) ^c^	**23**	16.0, CH_3_	1.60, s ^c^
**11**	135.3, C		**24**	16.3, CH_3_	1.60, s ^c^
**12**	39.7, CH_2_^a^	2.00, m ^c^	**25**	16.2, CH_3_	1.68, s ^c^
**13**	26.6, CH_2_^b^	2.06, m ^c^			

^a,b^ Values with identical superscript within each column may be interchanged; ^c^ Values with identical superscript within each column are mutually overlapped.

**Table 2:** ^1^H (600 MHz) and ^13^C NMR (150 MHz) spectroscopic data of **2** and **3**.

Position	2		3	
	δ_C_	δ_H_, Mult. (*J* in Hz)	δ_C_	δ_H_, Mult. (*J* in Hz)
**1**	117.9, CH	5.43, dd (4.2, 2.4)	118.4, CH	5.43, dd (4.2, 2.4)
**2**	22.4, CH_2_	2.03, m	22.9, CH_2_	2.03, m
**3a**	28.7, CH_2_	1.23, m	28.5, CH_2_	1.23, m
**3b**		1.41, m		1.41, m
**4**	33.2, C		33.9, C	
**5**	41.8, CH	1.59, m	42.5, CH	1.59, m
**6a**	29.4, CH_2_	1.13, m	29.9, CH_2_	1.13, m
**6b**		1.82, m		1.82, m
**7a**	30.8, CH_2_	1.44, m	31.3, CH_2_	1.44, m
**7b**		1.61, m		1.61, m
**8**	43.9, CH	1.34, m	44.4, CH	1.34, m
**9**	42.0, C		43.1, C	
**10**	144.4, C		145.0, C	
**11a**	27.5, CH_2_	1.35, m	28.2, CH_2_	1.35, m
**11b**		1.91, m		1.91, m
**12a**	21.7, CH_2_	2.00, m	22.3, CH_2_	2.00, m
**12b**		2.18, m		2.18, m
**13**	168.6, C		169.1, C	
**14**	116.9, CH	5.86, s	117.5, CH	5.86, s
**15**	170.3, C		170.8, C	
**16**	103.9, CH	5.56, s	104.4, CH	5.61, s
**16-OCH_3_**	56.1, CH_3_	3.53, s	56.5, CH_3_	3.50, s
**17**	23.5, CH_3_	0.86, s	23.9, CH_3_	0.84, s
**18**	15.9, CH_3_	0.88, d (7.2)	16.4, CH_3_	0.84, d (6.6)
**19**	22.4, CH_3_	1.06, s	23.0, CH_3_	1.04, s
**20a**	38.6, CH_2_	1.12, m	39.1, CH_2_	1.02, m
**20b**		1.33, m		1.39, m
**21**	21.9, CH_2_	1.86, m	22.4, CH_2_	1.90, m
**22**	124.6, CH	5.01, t (7.2)	125.2, CH	5.04, t (7.2)
**23**	130.9, C		130.9, C	
**24**	17.2, CH_3_	1.58, s	17.6, CH_3_	1.59, s
**25**	25.2, CH_3_	1.68, s	25.7, CH_3_	1.66, s

**Table 3:** ^1^H (600 MHz) and ^13^C NMR (150 MHz) spectroscopic data of **4** and **5**.

Position	4		5	
	δ_C_	δ_H_, Mult. (*J* in Hz)	δ_C_	δ_H_, Mult. (*J* in Hz)
**1**	118.0, CH	5.44, t (3.6)	118.5, CH	5.41, t (3.6)
**2**	22.4, CH_2_	1.99, m	23.0, CH_2_	1.98, m
**3a**	28.6, CH_2_	1.27, m	28.6, CH_2_	1.27, m
**3b**		1.38, m		1.38, m
**4**	33.4, C		34.0, C	
**5**	42.3, CH	1.55, m	43.4, CH	1.53, m
**6a**	29.3, CH_2_	1.13, m	29.9, CH_2_	1.11, m
**6b**		1.83, m		1.81, m
**7a**	30.8, CH_2_	1.44, m	31.3, CH_2_	1.42, m
**7b**		1.60, m		1.59, m
**8**	43.9, CH	1.37, m	44.4, CH	1.36, m
**9**	41.9, C		42.5, C	
**10**	144.4, C		145.1, C	
**11a**	27.6, CH_2_	1.33, m	28.1, CH_2_	1.32, m
**11b**		1.93, m		1.91, m
**12a**	21.9, CH_2_	1.95, m	22.5, CH_2_	2.01, m
**12b**		2.17, m		2.11, m
**13**	168.5, C		169.0, C	
**14**	117.0, CH	5.87, s	117.5, CH	5.85, s
**15**	170.2, C		170.8, C	
**16**	103.9, CH	5.65, s	104.5, CH	5.61, s
**16-OCH_3_**	56.0, CH_3_	3.55, s	56.8, CH_3_	3.54, s
**17**	23.5, CH_3_	0.85, s	24.0, CH_3_	0.83, s
**18**	15.8, CH_3_	0.88, d (7.2)	16.4, CH_3_	0.87, d (7.2)
**19**	22.5, CH_3_	1.06, s	23.0, CH_3_	1.04, s
**20a**	39.2, CH_2_	1.02, m	39.8, CH_2_	0.95, m
**20b**		1.34, m		1.33, m
**21**	17.2, CH_2_	1.23, m	17.8, CH_2_	1.23, m
		1.31, m		1.29, m
**22**	41.2, CH_2_	1.36, m	41.1, CH_2_	1.35, m
**23**	73.9, C		74.6, C	
**24**	24.1, CH_3_	1.12, s	24.9, CH_3_	1.11, s
**25**	24.6, CH_3_	1.13, s	25.0, CH_3_	1.12, s
**23-OCH_3_**	48.6, CH_3_	3.16, s	49.1, CH_3_	3.16, s

**Table 4:** Cytotoxic activities of compounds **1**–**7** (IC_50_ in μM).

Compound	DU145	SW1990	Huh7	PANC-1
**1**	2.87 ± 0.63	2.11 ± 0.21	2.87 ± 0.23	7.59 ± 0.62
**2**	>50	>50	>50	>50
**3**	13.35 ± 1.41	7.40 ± 0.59	2.37 ± 0.23	>50
**4**	>50	>50	>50	>50
**5**	>50	>50	>50	>50
**6**	3.21 ± 0.22	3.55 ± 0.31	3.61 ± 0.17	5.21 ± 0.55
**7**	>50	>50	>50	>50
Cisplatin	2.90 ± 0.39	5.09 ± 0.18	1.11 ± 0.11	4.59 ± 0.13

## Supplementary Materials

The following are available online at https://www.mdpi.com/1660-3397/18/10/491/s1, 1D and 2D NMR, UV, IR, and HRESMS data of **1**–**5**.
