# Supplementary material for: Dactylospenes A–E, Sesterterpenes from the Marine Sponge Dactylospongia elegans
Source: Mar Drugs. 2020 Sep 25;18(10):491. doi: 10.3390/md18100491 (PMC7600696; doi:10.3390/md18100491)

Supplementary data

Dactylospenes A–E, Sesterterpenes from the Marine Sponge Dactylospongia elegans

Hao-Bing Yu [1,†], Bin-Bin Gu [2,†], Arihiro Iwasaki [3,†], Wen-Li Jiang [4], Andrew Ecker [5], Shu-Ping Wang [2], Fan Yang [2,*] and Hou-Wen Lin [2,*]

Hao-Bing Yu [1,†], Bin-Bin Gu [2,†], Arihiro Iwasaki [3,†], Wen-Li Jiang [4], Andrew Ecker [5], Shu-Ping Wang [2], Fan Yang [2,*] and Hou-Wen Lin [2,*]

1 Department of Marine Biomedicine and Polar Medicine, Naval Medical Center of PLA, Second Military Medical University, Shanghai 200433, China; yuhaobing1986@126.com
2 Research Center for Marine Drugs, State Key Laboratory of Oncogenes and Related Genes, School of Medicine, Shanghai Jiao Tong University, Shanghai 200127, China; 18521018251@163.com (B.-B.G.); shupingwang2007@163.com (S.-P.W.)
3 Department of Chemistry, Faculty of Science and Technology, Keio University, 3-14-1 Hiyoshi, Kohoku-ku, Yokohama, Kanagawa 223-8522, Japan; a.iwasaki@chem.keio.ac.jp
4 Department of Biochemistry and Molecular Biology, College of Basic Medical Sciences, Second Military Medical University, Shanghai 200433, China; jwlsally@163.com
5 Center for Marine Biotechnology and Biomedicine, Scripps Institution of Oceanography, University of California, San Diego, CA 92093, USA; aecker@ucsd.edu
* Correspondence: Correspondence: yang-fan@sjtu.edu.cn (F.Y.); franklin67@126.com (H.-W.L.); Tel.: +86-21-6838-3346 (H.-W.L.)
† These authors contributed equally to this work.

S1 Quantum chemical CD calculation of models **8** and **9**.
S2 ^{1}H NMR spectrum of dactylospene A (**1**) in $CDCl_3$.
S3 ^{13}C NMR spectrum of dactylospene A (**1**) in $CDCl_3$.
S4 DEPT135 spectrum of dactylospene A (**1**) in $CDCl_3$.
S5 HSQC spectrum of dactylospene A (**1**) in $CDCl_3$.
S6 COSY spectrum of dactylospene A (**1**) in $CDCl_3$.
S7 HMBC spectrum of dactylospene A (**1**) in $CDCl_3$.
S8 NOESY spectrum of dactylospene A (**1**) in $CDCl_3$.
S9 HRESIMS of dactylospene A (**1**).
S10 UV spectrum of dactylospene A (**1**).
S11 IR spectrum of dactylospene A (**1**).
S12 ^{1}H NMR spectrum of dactylospene B (**2**) in $CDCl_3$.
S13 ^{13}C NMR spectrum of dactylospene B (**2**) in $CDCl_3$.
S14 DEPT135 spectrum of dactylospene B (**2**) in $CDCl_3$.
S15 HSQC spectrum of dactylospene B (**2**) in $CDCl_3$.
S16 COSY spectrum of dactylospene B (**2**) in $CDCl_3$.
S17 HMBC spectrum of dactylospene B (**2**) in $CDCl_3$.
S18 NOESY spectrum of dactylospene B (**2**) in $CDCl_3$.
S19 HRESIMS of dactylospene B (**2**).
S20 UV spectrum of dactylospene B (**2**).
S21 IR spectrum of dactylospene B (**2**).
S22 ^{1}H NMR spectrum of dactylospene C (**3**) in $CDCl_3$.

S23 ^{13}C NMR spectrum of dactylospene C (**3**) in $CDCl_3$.
S24 DEPT135 spectrum of dactylospene C (**3**) in $CDCl_3$.
S25 HSQC spectrum of dactylospene C (**3**) in $CDCl_3$.
S26 COSY spectrum of dactylospene C (**3**) in $CDCl_3$.
S27 HMBC spectrum of dactylospene C (**3**) in $CDCl_3$.
S28 NOESY spectrum of dactylospene C (**3**) in $CDCl_3$.
S29 HRESIMS of dactylospene C (**3**).
S30 UV spectrum of dactylospene C (**3**).
S31 IR spectrum of dactylospene C (**3**).
S32 ^{1}H NMR spectrum of dactylospene D (**4**) in $CDCl_3$.
S33 ^{13}C NMR spectrum of dactylospene D (**4**) in $CDCl_3$.
S34 DEPT135 spectrum of dactylospene D (**4**) in $CDCl_3$.
S35 HSQC spectrum of dactylospene D (**4**) in $CDCl_3$.
S36 COSY spectrum of dactylospene D (**4**) in $CDCl_3$.
S37 HMBC spectrum of dactylospene D (**4**) in $CDCl_3$.
S38 NOESY spectrum of dactylospene D (**4**) in $CDCl_3$.
S39 HRESIMS of dactylospene D (**4**).
S40 UV spectrum of dactylospene D (**4**).
S41 IR spectrum of dactylospene D (**4**).
S42 ^{1}H NMR spectrum of dactylospene E (**5**) in $CDCl_3$.
S43 ^{13}C NMR spectrum of dactylospene E (**5**) in $CDCl_3$.
S44 DEPT135 spectrum of dactylospene E (**5**) in $CDCl_3$.
S45 HSQC spectrum of dactylospene E (**5**) in $CDCl_3$.
S46 COSY spectrum of dactylospene E (**5**) in $CDCl_3$.
S47 HMBC spectrum of dactylospene E (**5**) in $CDCl_3$.
S48 NOESY spectrum of dactylospene E (**5**) in $CDCl_3$.
S49 HRESIMS of dactylospene E (**5**).
S50 UV spectrum of dactylospene E (**5**).
S51 IR spectrum of dactylospene E (**5**).
S52 CD spectra of dactylospenes A (**1**).

S1 Quantum chemical CD calculation of models **8** and **9**.

Table S1. Gibbs Free Energies and Equilibrium Populations of Low-energy Conformers (≥ 1%) of model **8** in MeCN (PCM).

Conformers	Δ*G*	P (%)
**A**	0.00	87.2%
**B**	1.13	12.8%

[a] B3LYP/6-31G(d), in kcal/mol. [b]From Δ*G* values at 298.15K.

Figure S1. Conformations of Low-energy Conformers (≥ 1%) of model **8** Calculated at B3LYP/6-31G(d) Level of Theory in MeCN (PCM).

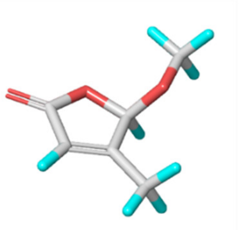

A 87.2%

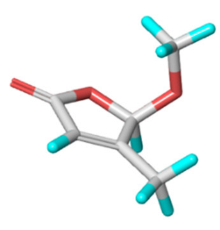

B 12.8%

Table S2. Cartesian Coordinates, Relative Thermal Free Energies (B3LYP/6-31G(d) PCM/MeOH), and Equilibrium Populations of Low-energy Conformers of model **8** in MeCN (PCM).

Conformation **A**

Δ*G* = 0.00 kcal/mol   P (%) = 87.2%

1	C	-1.61669	-0.56594	-0.02554
2	C	-1.42922	0.89437	-0.18842
3	C	-0.15868	1.21506	0.07411
4	C	0.57701	-0.05771	0.44794
5	O	-0.40277	-1.10155	0.36627
6	O	-2.60236	-1.24002	-0.17207
7	O	1.63042	-0.25472	-0.43712
8	C	2.43433	-1.39333	-0.13428
9	C	0.54493	2.52798	0.01614
10	H	-2.23784	1.54538	-0.4954
11	H	0.94169	-0.05182	1.48853
12	H	3.26158	-1.38475	-0.84664
13	H	2.83365	-1.33232	0.88894
14	H	1.86226	-2.32085	-0.24351
15	H	-0.12668	3.33232	-0.29521
16	H	0.96921	2.78903	0.99468
17	H	1.38373	2.47083	-0.68773

Conformation **B**

Δ*G*= 1.13 kcal/mol   P (%) = 12.8%

1	C	-1.64795	0.03627	-0.03851
2	C	-0.82779	1.20816	-0.42614
3	C	0.4114	1.0589	0.05237

4	C	0.47104	-0.25584	0.82174
5	O	-0.84223	-0.81665	0.69359
6	O	-2.80368	-0.20674	-0.26957
7	O	1.46615	-1.14026	0.40833
8	C	1.35015	-1.62587	-0.93015
9	C	1.60883	1.94133	-0.06047
10	H	-1.23381	2.02273	-1.01297
11	H	0.66619	-0.09428	1.8881
12	H	2.20966	-2.28129	-1.08174
13	H	0.42621	-2.19758	-1.06359
14	H	1.38208	-0.81196	-1.66677
15	H	1.41465	2.80856	-0.69708
16	H	1.9182	2.30194	0.92958
17	H	2.46077	1.38334	-0.46738

Table S3. Gibbs Free Energies and Equilibrium Populations of Low-energy Conformers (≥ 1%) of model **9** in MeCN (PCM).

Conformers	ΔG	P (%)
**A**	0.00	67.2%
**B**	0.80	17.4%
**C**	0.95	13.5%
**D**	2.50	1.0%

[a] B3LYP/6-31G(d), in kcal/mol. [b]From ΔG values at 298.15K.

Figure S2. Conformations of Low-energy Conformers (≥ 1%) of model **9** Calculated at B3LYP/6-31G(d) Level of Theory in MeCN (PCM).

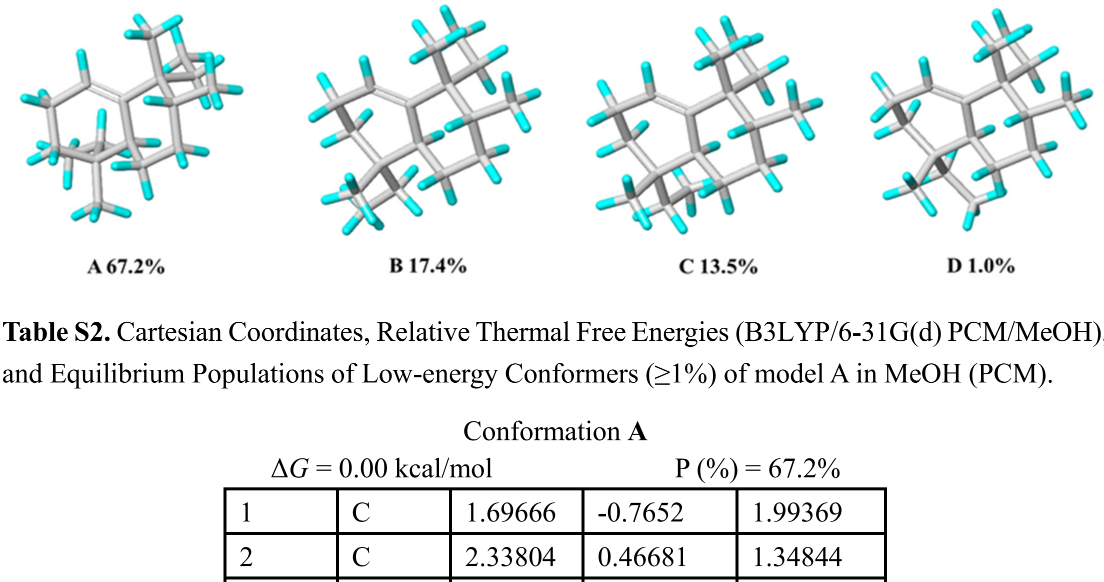

Table S2. Cartesian Coordinates, Relative Thermal Free Energies (B3LYP/6-31G(d) PCM/MeOH), and Equilibrium Populations of Low-energy Conformers (≥1%) of model A in MeOH (PCM).

Conformation **A**

ΔG = 0.00 kcal/mol P (%) = 67.2%

1	C	1.69666	-0.7652	1.99369
2	C	2.33804	0.46681	1.34844
3	C	2.02563	0.56486	-0.15987
4	C	0.47208	0.64548	-0.35168
5	C	-0.30372	-0.35769	0.50452
6	C	0.26857	-0.96353	1.55642

7	C	-0.16277	2.03826	-0.1237
8	C	-1.66498	2.01299	-0.43023
9	C	-2.43815	0.92269	0.33455
10	C	-1.79916	-0.5106	0.1605
11	C	-3.93499	1.01523	-0.00732
12	C	-1.30487	-2.36521	-1.62247
13	C	-1.99425	-1.03392	-1.29699
14	C	2.7194	1.8116	-0.74064
15	C	2.52641	-0.69458	-0.92926
16	C	4.00693	-1.06813	-0.77262
17	C	-2.51323	-1.48282	1.1215
18	H	0.28931	0.40419	-1.41011
19	H	2.28113	-1.66969	1.76115
20	H	1.73295	-0.67585	3.08867
21	H	3.42222	0.46554	1.51606
22	H	1.95497	1.36949	1.84315
23	H	-0.3092	-1.65388	2.16472
24	H	0.31575	2.79182	-0.75862
25	H	-0.0048	2.34926	0.91835
26	H	-2.10486	2.99044	-0.19
27	H	-1.81054	1.87908	-1.51192
28	H	-2.33299	1.15082	1.40659
29	H	-4.12113	0.84905	-1.07469
30	H	-4.30855	2.01787	0.23338
31	H	-4.54138	0.29924	0.55592
32	H	-1.49817	-2.64837	-2.6638
33	H	-0.2199	-2.30271	-1.48926
34	H	-1.6687	-3.1817	-0.98951
35	H	-3.07082	-1.14554	-1.47606
36	H	-1.65203	-0.28161	-2.01653
37	H	2.46421	2.71827	-0.18253
38	H	3.80881	1.70643	-0.69823
39	H	2.44256	1.97296	-1.7906
40	H	1.91711	-1.55618	-0.63207
41	H	2.31786	-0.53527	-1.99692
42	H	4.23541	-1.95516	-1.37489
43	H	4.67712	-0.2688	-1.10731
44	H	4.26581	-1.306	0.26508
45	H	-2.03634	-2.46741	1.13566
46	H	-2.51808	-1.10077	2.1486
47	H	-3.55351	-1.63296	0.81659

Conformation **B**

ΔG = 0.80 kcal/mol P (%) = 17.4%

1	C	1.44303	2.11102	-1.09724
2	C	2.29212	1.39971	-0.04252
3	C	2.08369	-0.12922	-0.06972
4	C	0.56519	-0.43607	0.20759
5	C	-0.42424	0.58098	-0.37324
6	C	0.00232	1.68336	-1.00972
7	C	0.16139	-1.85994	-0.24047
8	C	-1.2902	-2.18548	0.10192
9	C	-2.26586	-1.17307	-0.51897
10	C	-1.9255	0.30975	-0.11239
11	C	-3.71334	-1.6179	-0.24473
12	C	-1.84821	1.90448	1.98312
13	C	-2.23175	0.5357	1.40688
14	C	2.536	-0.6741	-1.44397
15	C	2.9161	-0.78818	1.06595
16	C	4.44391	-0.71097	0.94081
17	C	-2.8258	1.25337	-0.93617
18	H	0.44449	-0.41234	1.30372
19	H	1.50131	3.19862	-0.95083
20	H	1.84676	1.93448	-2.10606
21	H	2.01356	1.78017	0.95088
22	H	3.35202	1.64093	-0.1883
23	H	-0.71575	2.37008	-1.44797
24	H	0.82836	-2.60246	0.2146
25	H	0.28682	-1.94788	-1.32674
26	H	-1.53815	-3.19058	-0.26543
27	H	-1.4238	-2.22253	1.19224
28	H	-2.11455	-1.21729	-1.6094
29	H	-3.93147	-1.66311	0.82832
30	H	-3.86871	-2.62604	-0.64738
31	H	-4.45696	-0.96564	-0.71226
32	H	-2.12025	1.95457	3.04402
33	H	-0.7731	2.09286	1.9021
34	H	-2.36539	2.72581	1.47521
35	H	-3.30769	0.38436	1.55891
36	H	-1.73471	-0.23822	2.00314
37	H	1.83874	-0.39488	-2.24049
38	H	3.52034	-0.27476	-1.7132
39	H	2.61546	-1.76644	-1.44311
40	H	2.61784	-0.32508	2.01752

41	H	2.63363	-1.84657	1.14471
42	H	4.91684	-1.18169	1.8105
43	H	4.80953	-1.23237	0.04957
44	H	4.80477	0.32246	0.89493
45	H	-2.64313	2.30671	-0.70773
46	H	-2.66824	1.11311	-2.01182
47	H	-3.88157	1.06309	-0.72245

Conformation **C**

ΔG = 0.95 kcal/mol P (%) = 13.5%

1	C	1.57563	2.06371	-1.19139
2	C	2.4429	1.27009	-0.21284
3	C	2.15467	-0.24329	-0.29392
4	C	0.64873	-0.49493	0.07231
5	C	-0.32078	0.58417	-0.42706
6	C	0.12374	1.69047	-1.04513
7	C	0.15794	-1.88812	-0.38406
8	C	-1.28875	-2.15519	0.02306
9	C	-2.24464	-1.0843	-0.52651
10	C	-1.81893	0.37099	-0.10134
11	C	-3.69657	-1.47181	-0.19475
12	C	-1.57155	1.90528	2.02673
13	C	-2.04254	0.57023	1.43627
14	C	2.48243	-0.7303	-1.72562
15	C	3.09925	-1.01664	0.67099
16	C	3.12784	-0.58598	2.14399
17	C	-2.71433	1.37423	-0.85749
18	H	0.58438	-0.49302	1.17154
19	H	1.68656	3.14019	-0.99989
20	H	1.91768	1.91844	-2.22735
21	H	2.23252	1.63062	0.80217
22	H	3.50962	1.45476	-0.4013
23	H	-0.5843	2.42213	-1.42272
24	H	0.81207	-2.66867	0.02397
25	H	0.2263	-1.95906	-1.47654
26	H	-1.60121	-3.13866	-0.3535
27	H	-1.37152	-2.21261	1.11753
28	H	-2.14623	-1.1069	-1.62365
29	H	-3.86702	-1.53458	0.88605
30	H	-3.91541	-2.46169	-0.6129
31	H	-4.43098	-0.77618	-0.61147
32	H	-1.79034	1.93843	3.10054

33	H	-0.49426	2.04932	1.89808
34	H	-2.07547	2.76157	1.56535
35	H	-3.11569	0.46198	1.63721
36	H	-1.55266	-0.23987	1.98883
37	H	1.75633	-0.37302	-2.46277
38	H	3.47338	-0.36885	-2.02748
39	H	2.50387	-1.82345	-1.7854
40	H	2.85433	-2.08613	0.62147
41	H	4.1192	-0.93112	0.26927
42	H	3.80412	-1.23661	2.71058
43	H	3.48696	0.44133	2.26634
44	H	2.14267	-0.65225	2.61955
45	H	-2.47779	2.41228	-0.60936
46	H	-2.61062	1.25861	-1.94256
47	H	-3.76663	1.22141	-0.60108

Conformation **D**

ΔG = 2.50 kcal/mol P (%) = 1.0%

1	C	1.36403	2.39747	-0.94418
2	C	2.26332	1.6599	0.04634
3	C	2.1736	0.12208	-0.09128
4	C	0.67341	-0.33316	0.07866
5	C	-0.38609	0.70448	-0.31575
6	C	-0.04957	1.89134	-0.84338
7	C	0.34547	-1.64982	-0.66914
8	C	-1.07379	-2.13583	-0.38018
9	C	-2.13592	-1.08491	-0.7401
10	C	-1.85693	0.30449	-0.05323
11	C	-3.54045	-1.6668	-0.50361
12	C	-1.73693	1.45212	2.31
13	C	-2.10496	0.2095	1.48926
14	C	2.7655	-0.27451	-1.46195
15	C	3.05994	-0.46095	1.04773
16	C	3.14973	-1.98337	1.21503
17	C	-2.8422	1.33752	-0.63741
18	H	0.53646	-0.54354	1.15162
19	H	1.37663	3.47447	-0.72712
20	H	1.74911	2.30538	-1.97117
21	H	1.961	1.945	1.06397
22	H	3.30906	1.97451	-0.07126
23	H	-0.82213	2.58093	-1.16976
24	H	1.06456	-2.43158	-0.40555

25	H	0.44551	-1.48194	-1.74906
26	H	-1.27208	-3.05192	-0.95308
27	H	-1.1652	-2.41882	0.67834
28	H	-2.03977	-0.89687	-1.82123
29	H	-3.70052	-1.93471	0.54702
30	H	-3.66162	-2.58425	-1.09211
31	H	-4.34074	-0.98316	-0.80251
32	H	-1.96447	1.2851	3.3694
33	H	-0.67197	1.69208	2.22851
34	H	-2.29805	2.33728	1.99135
35	H	-3.16849	-0.00896	1.64668
36	H	-1.56154	-0.64837	1.90174
37	H	2.16123	0.10042	-2.29451
38	H	3.7732	0.14679	-1.56717
39	H	2.84968	-1.35778	-1.58539
40	H	4.07754	-0.07083	0.90042
41	H	2.71159	-0.03144	1.99811
42	H	3.87087	-2.22836	2.0038
43	H	2.1909	-2.4246	1.50734
44	H	3.48695	-2.48457	0.30089
45	H	-2.68488	2.33804	-0.22517
46	H	-2.74735	1.40698	-1.72719
47	H	-3.87449	1.05746	-0.40781

S2 ^{1}H NMR spectrum of dactylospene A (**1**) in $CDCl_3$.

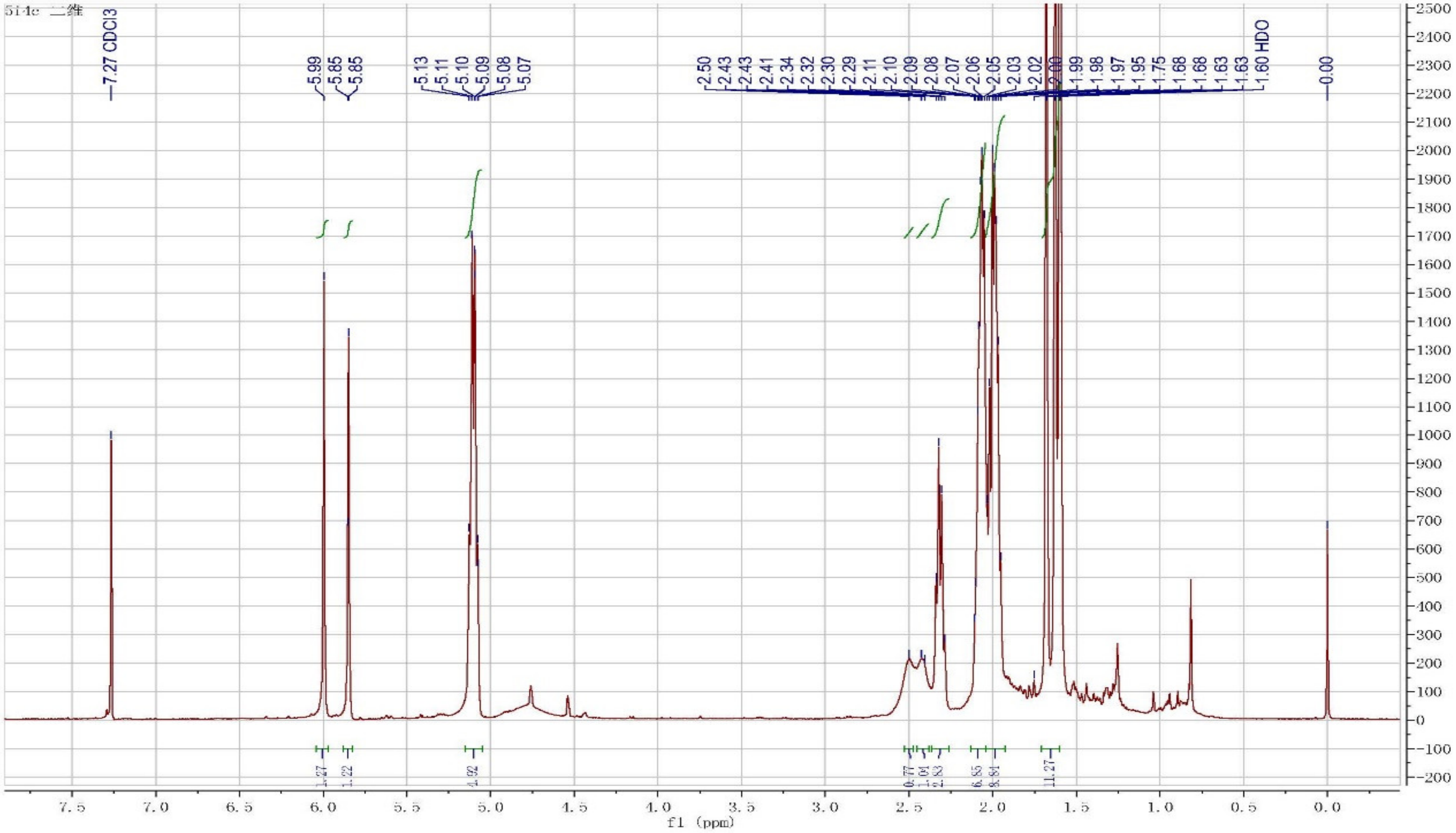

S3 ^{13}C NMR spectrum of dactylospene A (**1**) in $CDCl_3$.

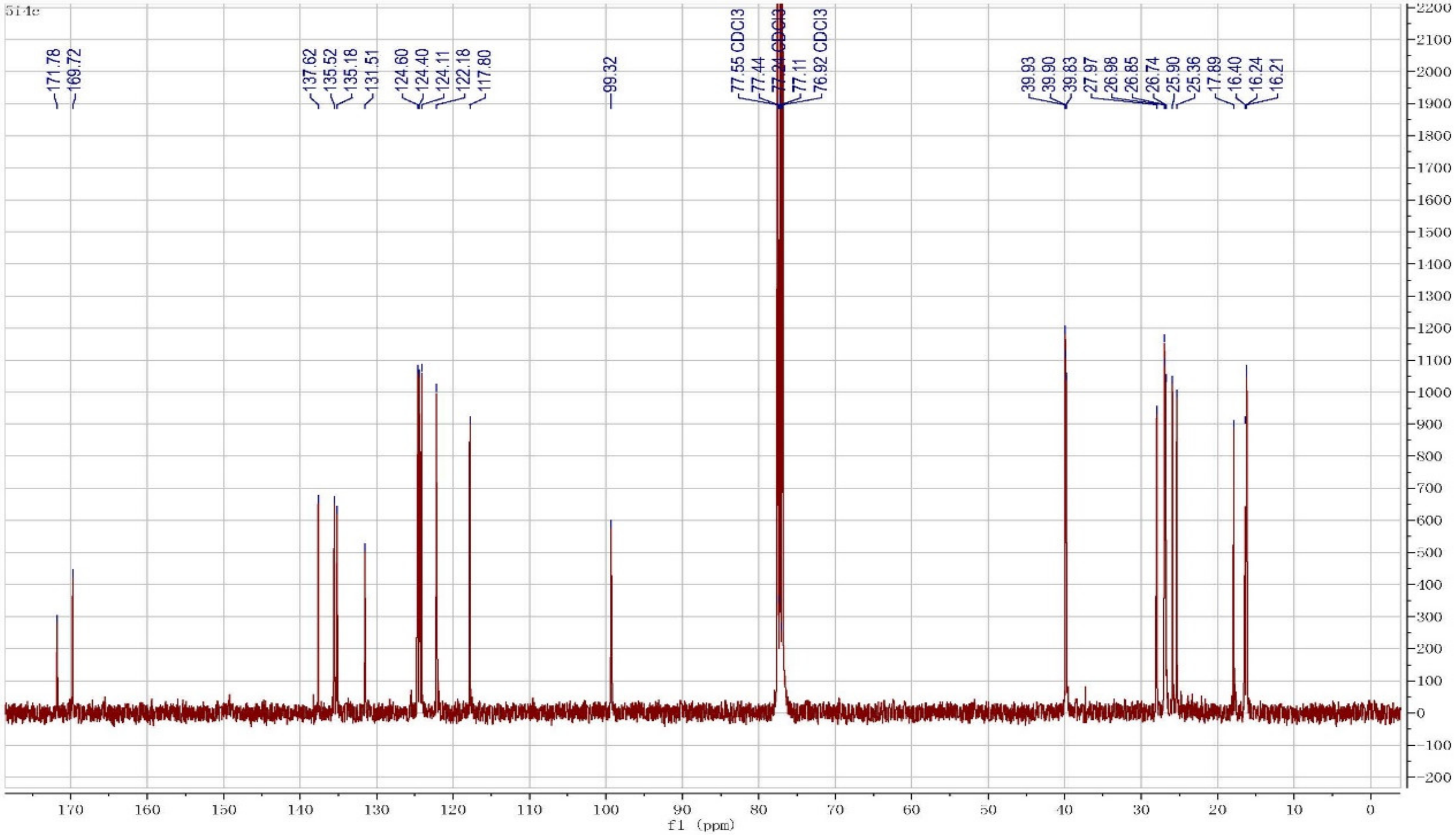

S4 DEPT135 spectrum of dactylospene A (**1**) in $CDCl_3$.

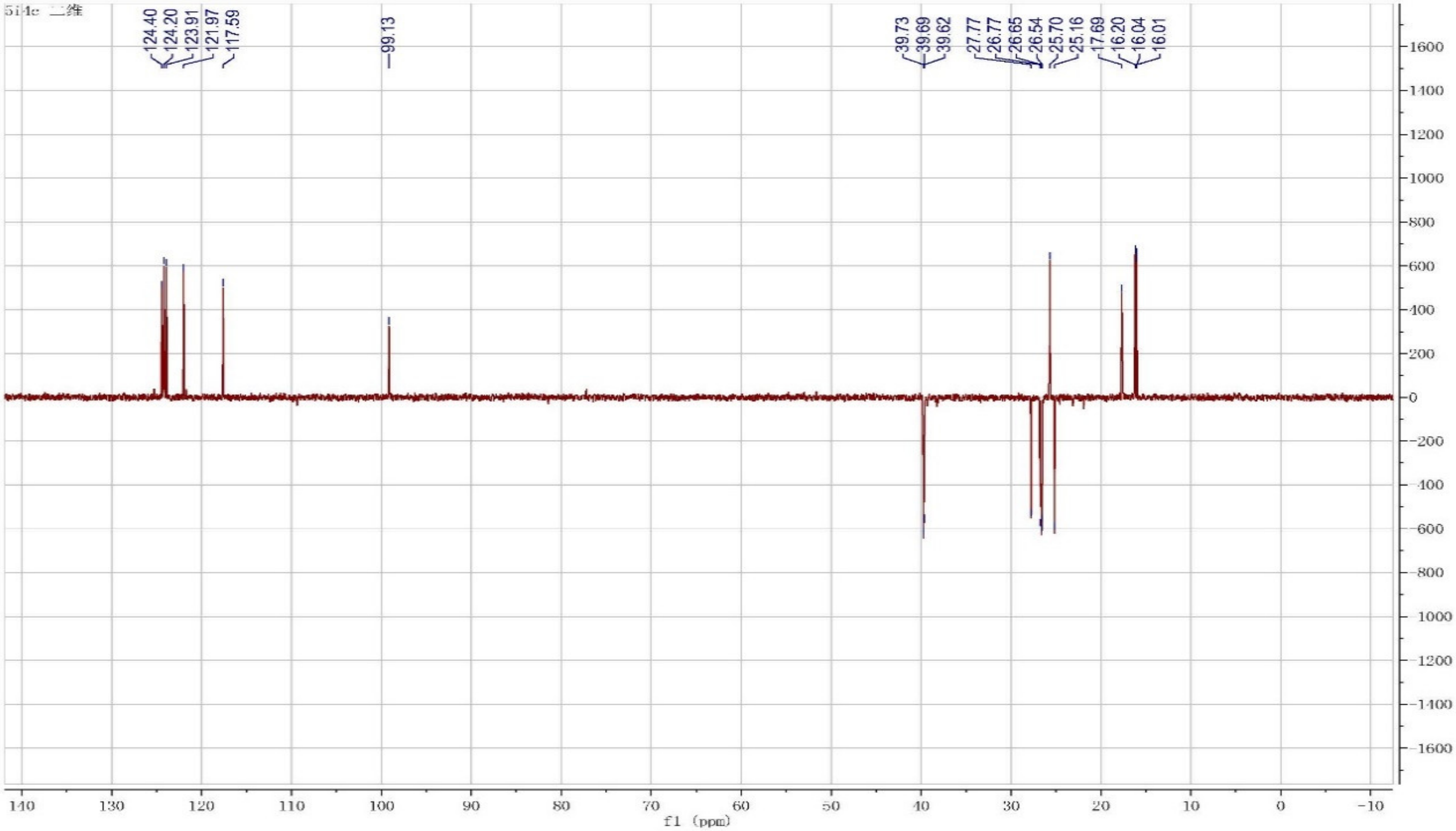

S5 HSQC spectrum of dactylospene A (**1**) in $CDCl_3$.

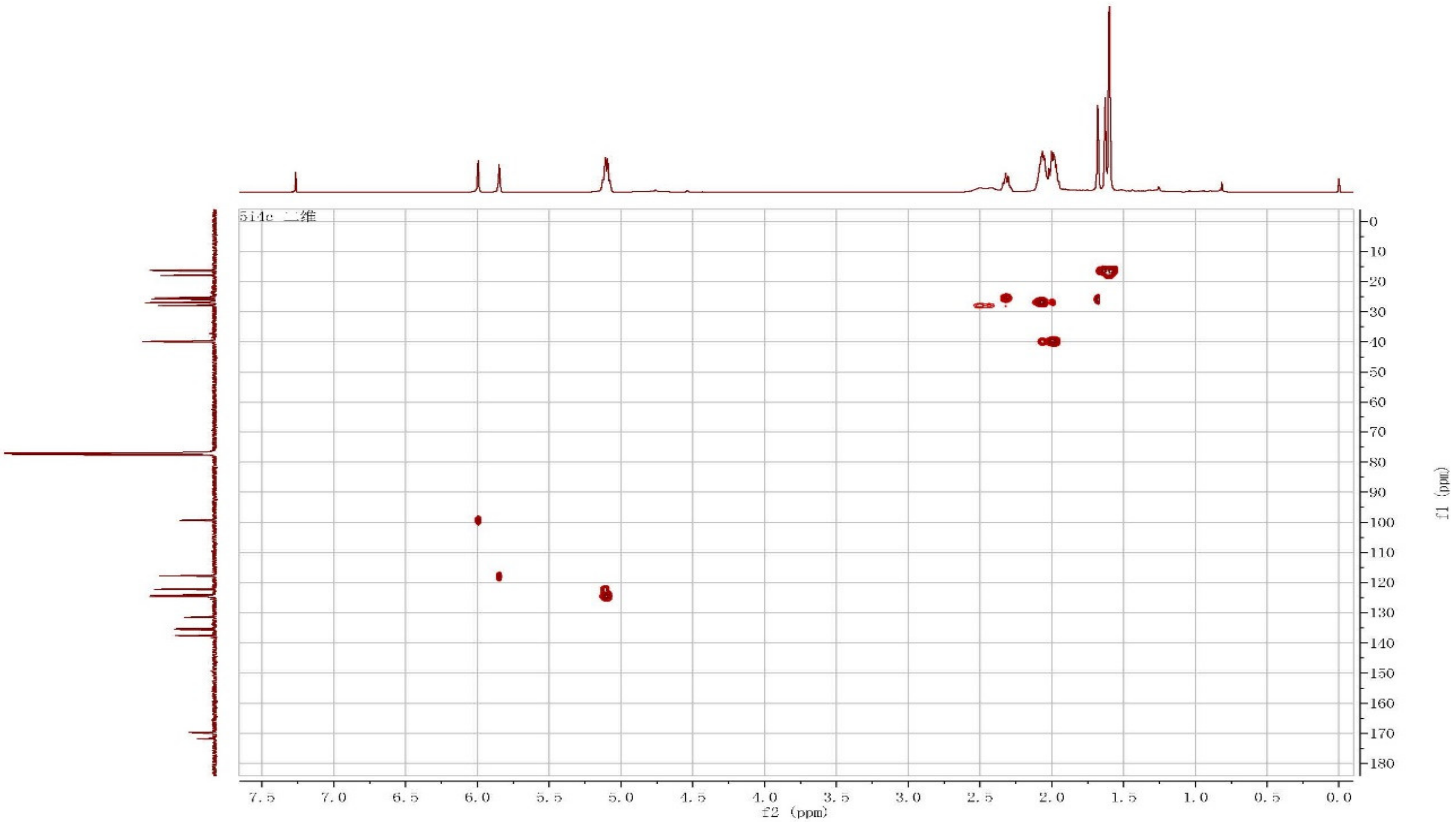

S6 COSY spectrum of dactylospene A (**1**) in $CDCl_3$.

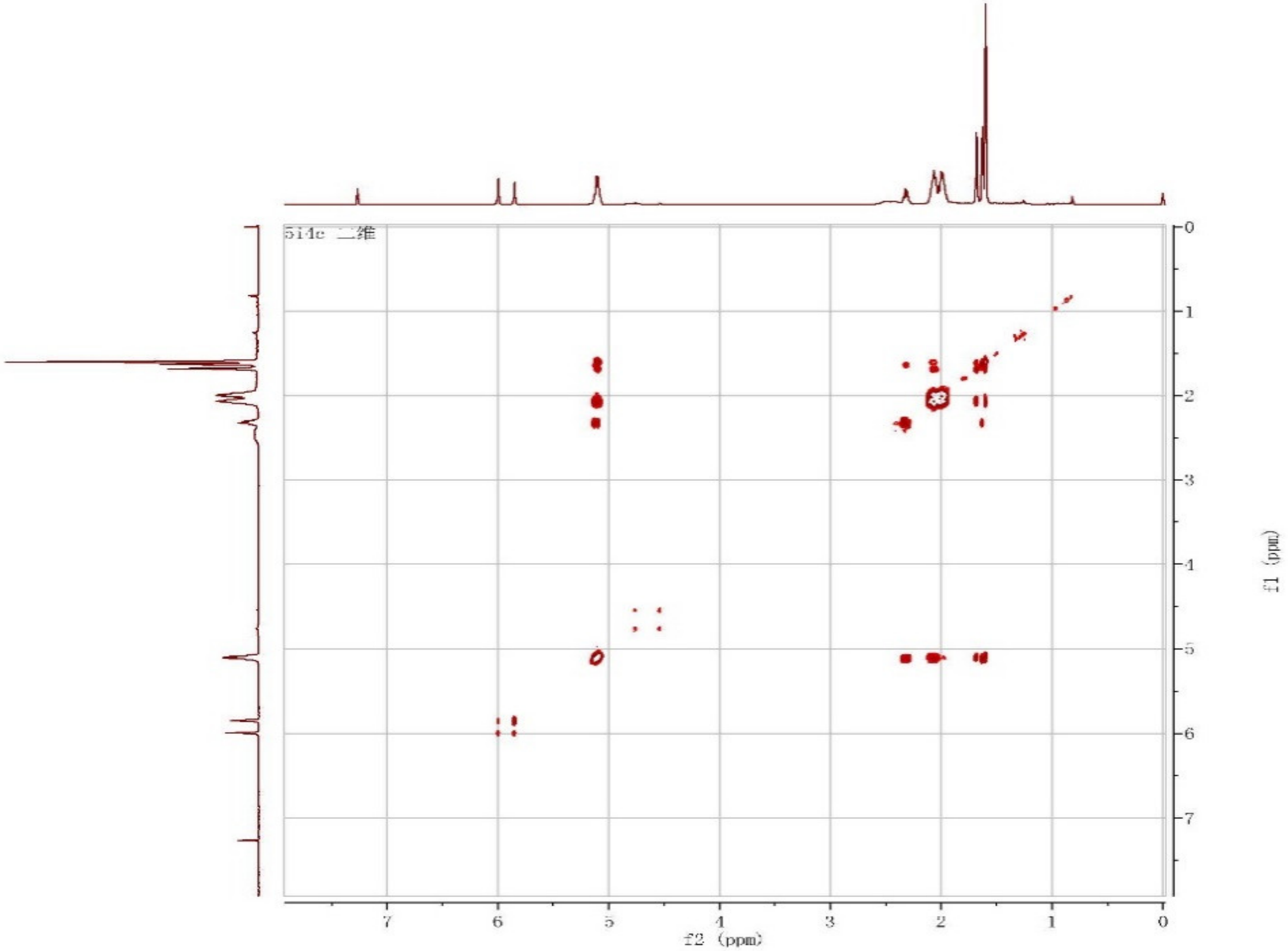

S7 HMBC spectrum of dactylospene A (**1**) in $CDCl_3$.

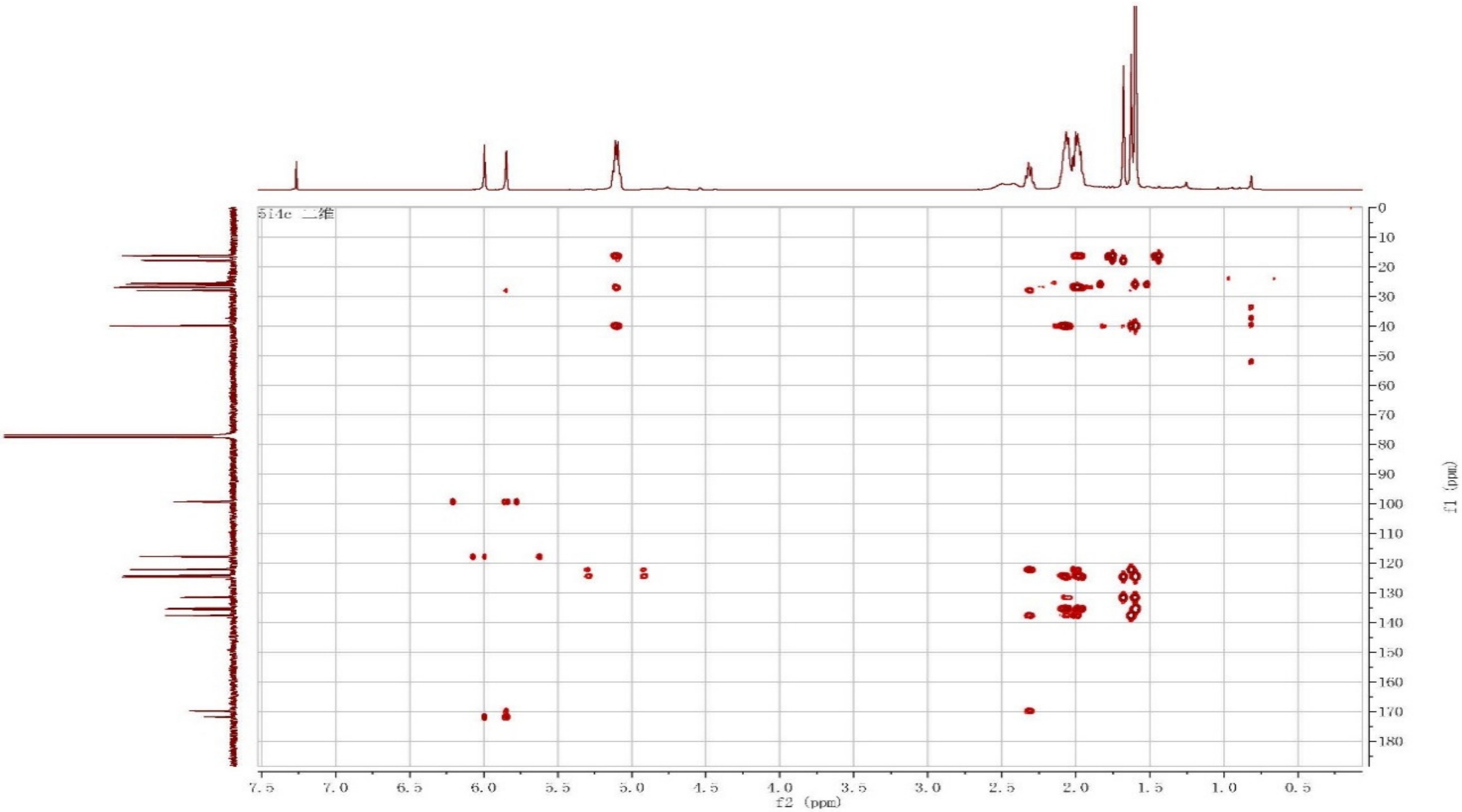

S8 NOESY spectrum of dactylospene A (**1**) in $CDCl_3$.

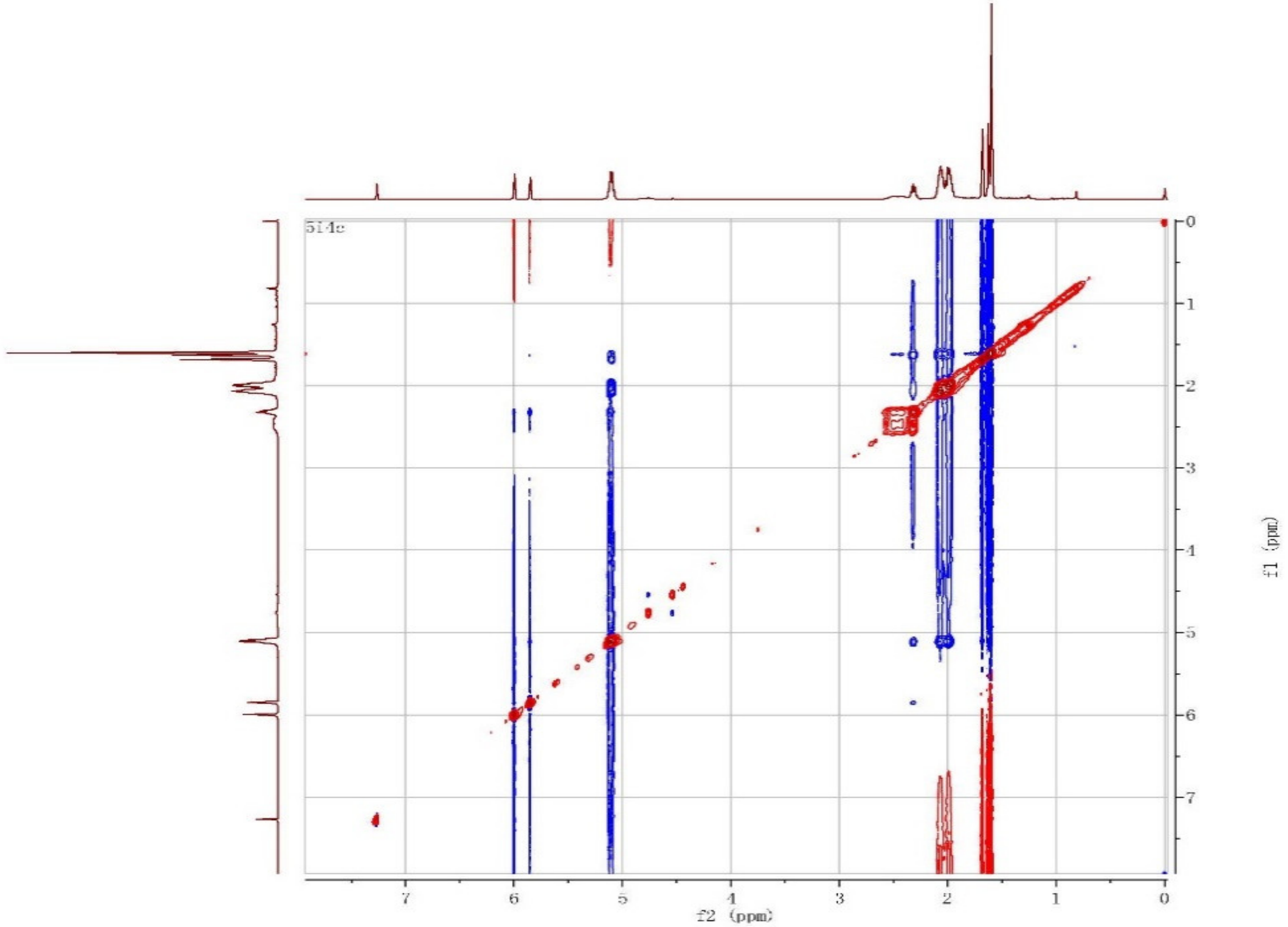

S9 HRESIMS of dactylospene A (**1**).

User Spectra

Fragmentor Voltage	Collision Energy	Ionization Mode
150	0	ESI

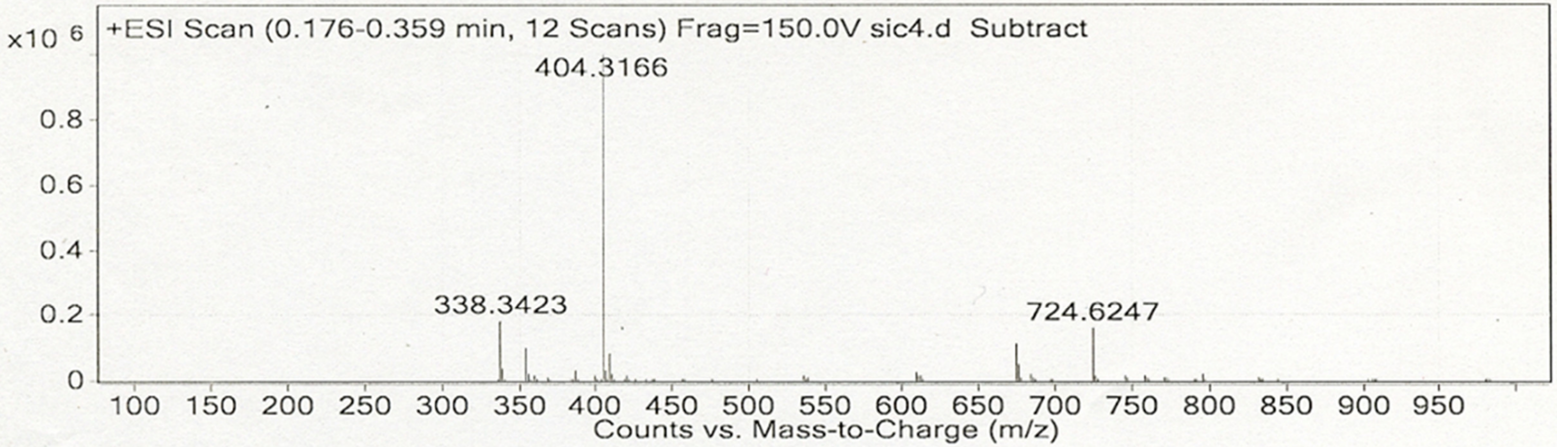

Peak List

m/z	z	Abund	Formula	Ion
338.3423		180165.3		
355.3689		98033.5		
404.3166	1	1002450.3	C25 H42 N O3	(M+NH4)+
405.3199	1	235555.9	C25 H42 N O3	(M+NH4)+
409.2719		85217.7		
675.6769		112809.9		
724.6247	1	159267.5		
725.6281	1	74348.1		

Formula Calculator Results

IonFormula	Measured Mass	Tgt Mass	Diff (ppm)	Score
C25 H42 N O3	404.3166	404.3159	-1.65	95.4

S10 UV spectrum of dactylospene A (**1**).

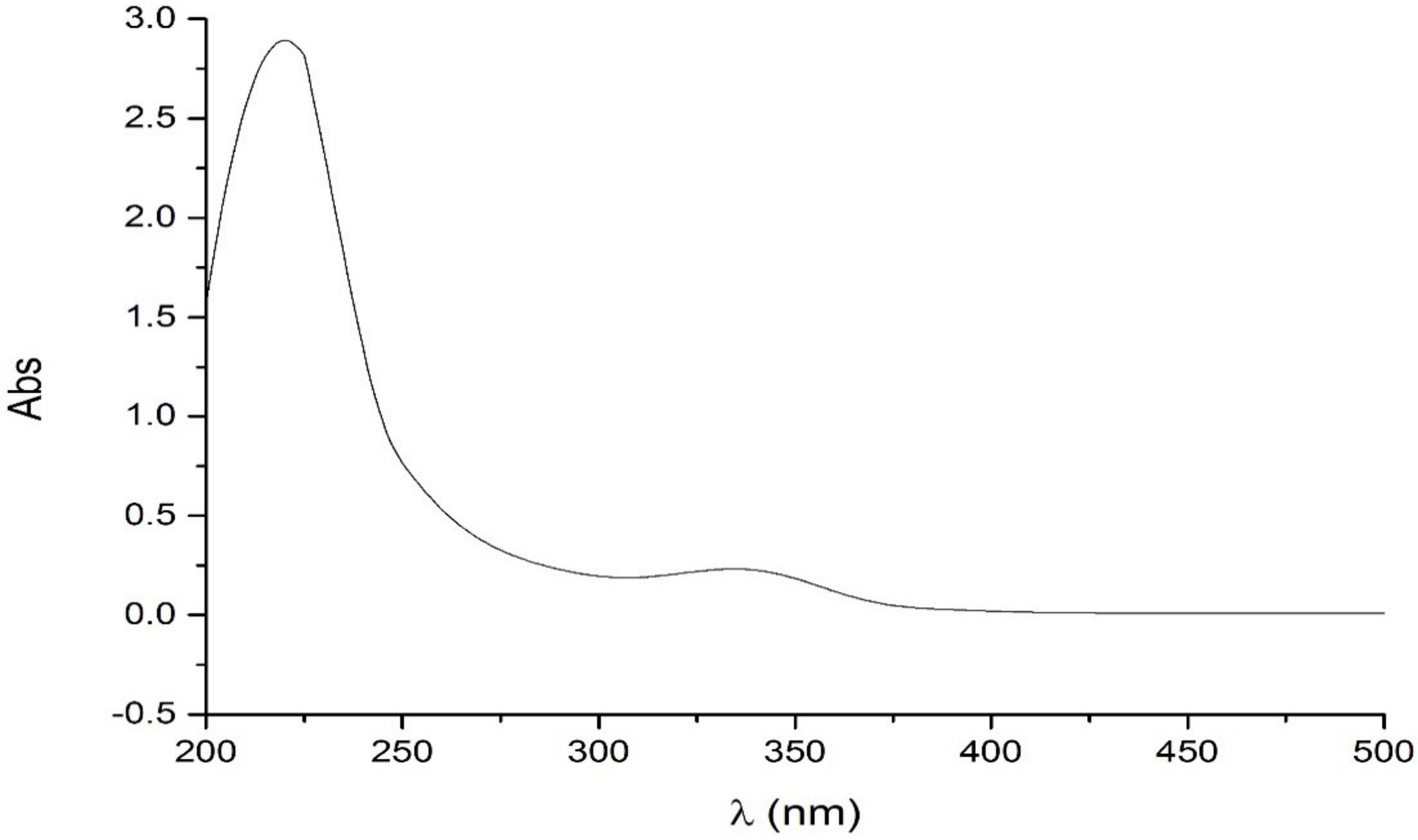

S11 IR spectrum of dactylospene A (**1**).

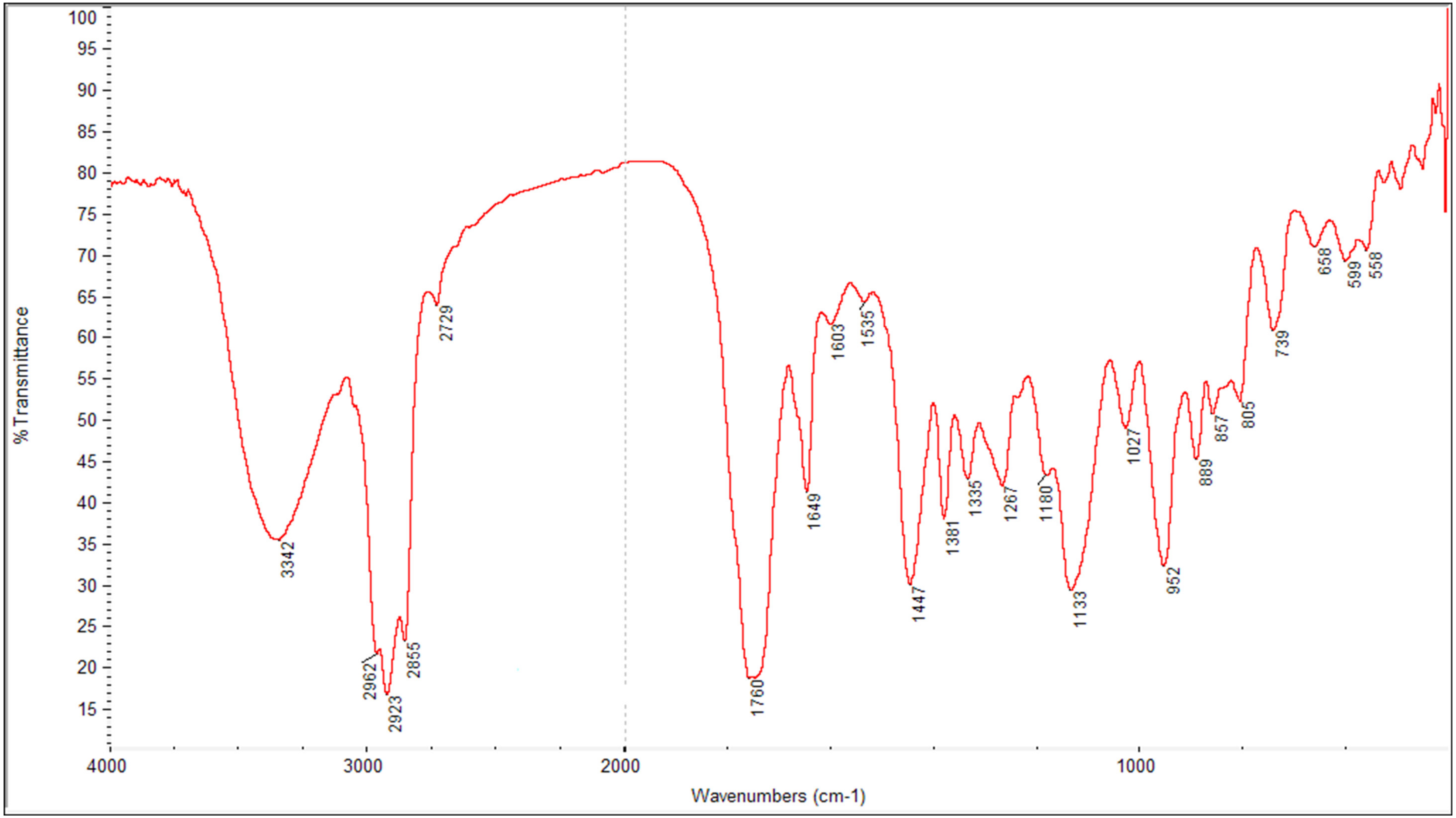

S12 ^{1}H NMR spectrum of dactylospene B (**2**) in $CDCl_3$.

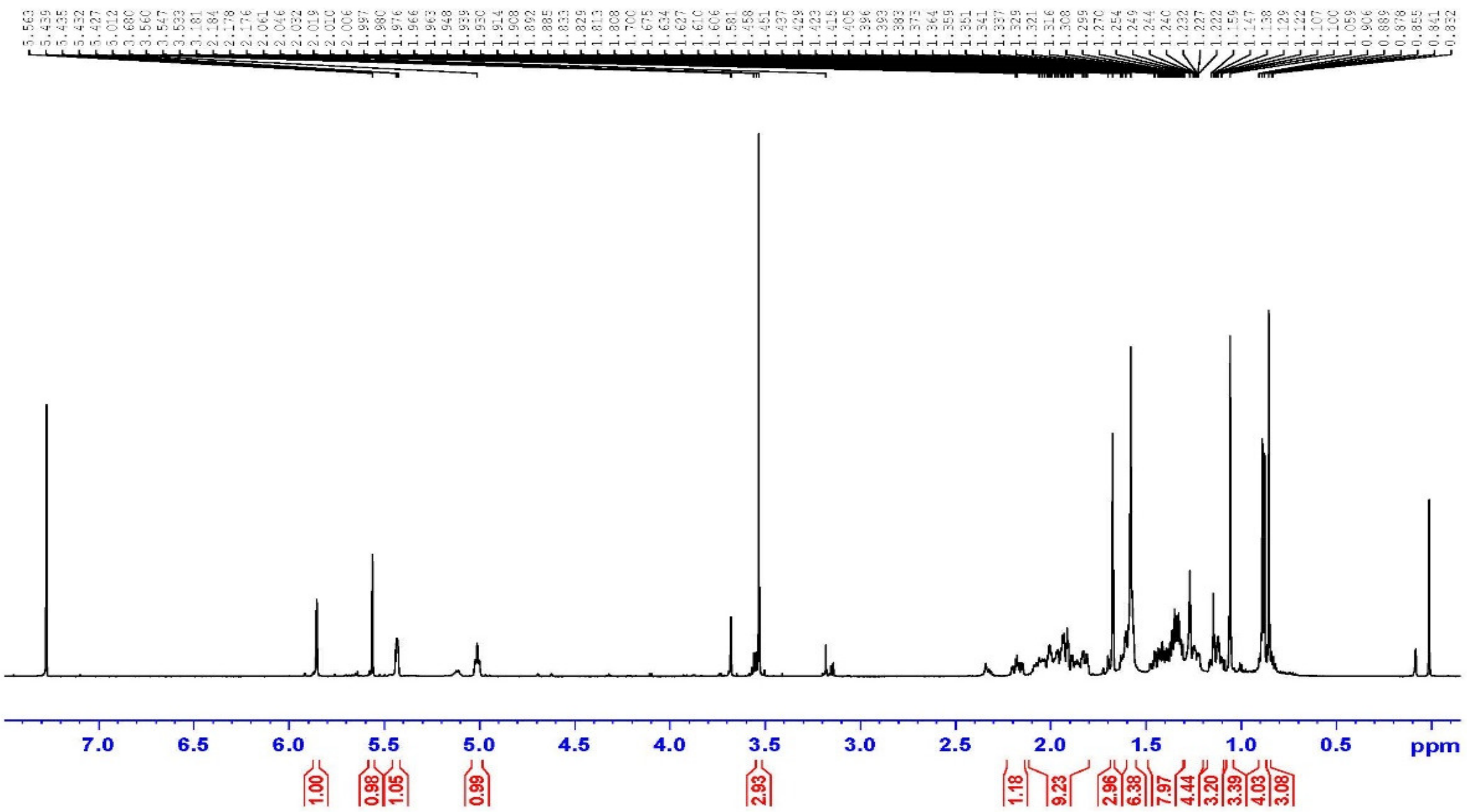

S13 ^{13}C NMR spectrum of dactylospene B (**2**) in $CDCl_3$.

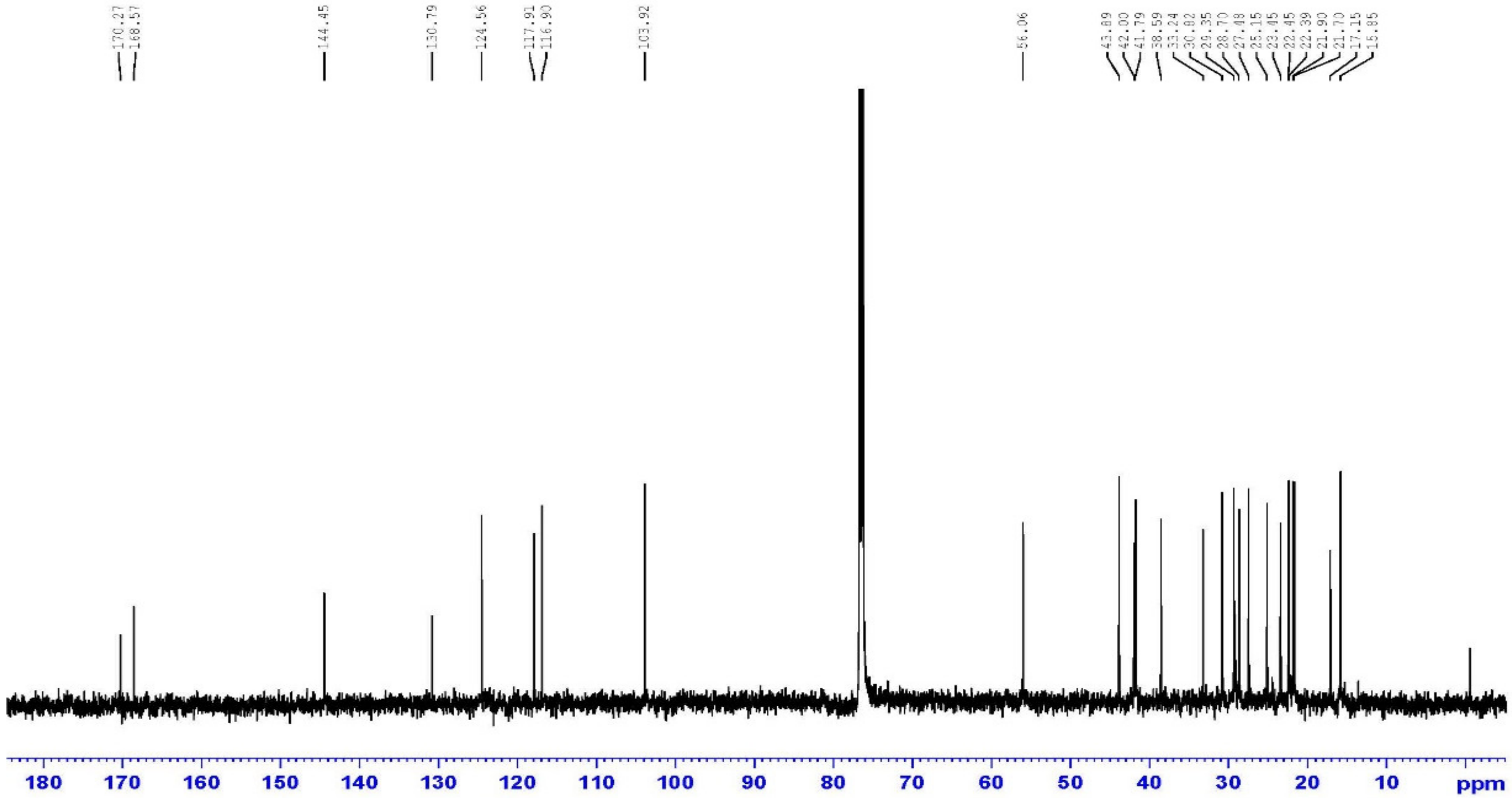

S14 DEPT135 spectrum of dactylospene B (**2**) in $CDCl_3$.

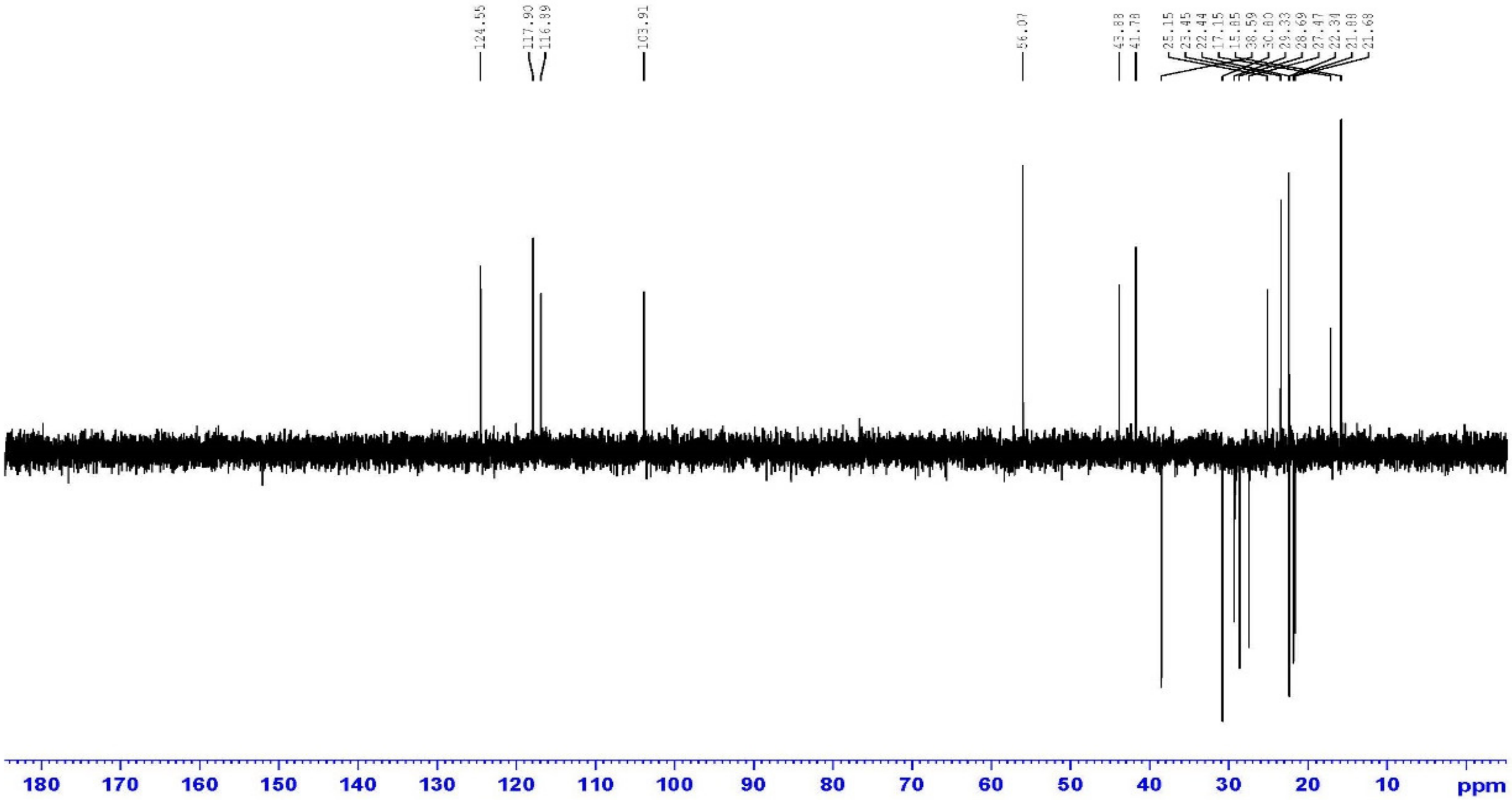

S15 HSQC spectrum of dactylospene B (**2**) in $CDCl_3$.

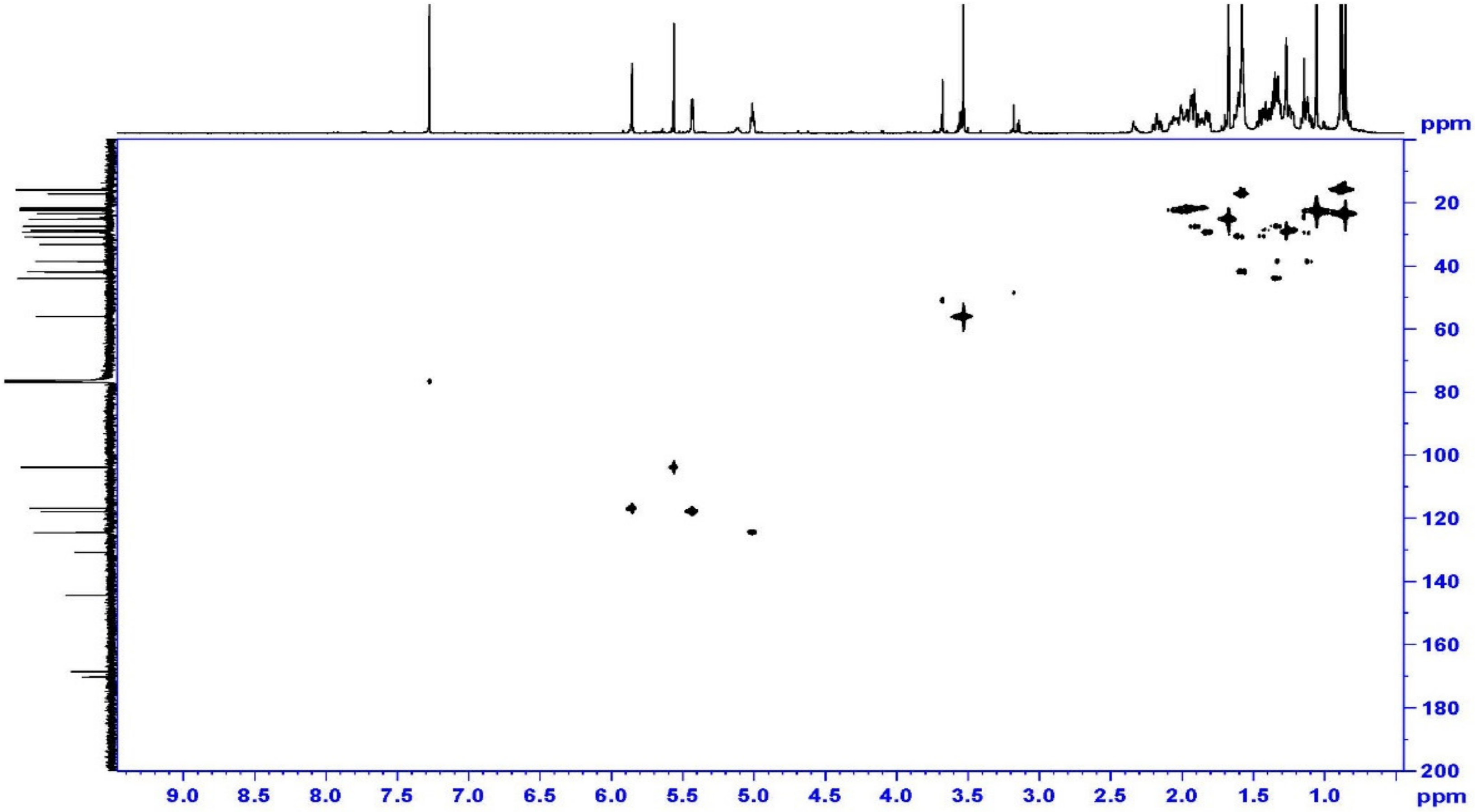

S16 COSY spectrum of dactylospene B (**2**) in $CDCl_3$.

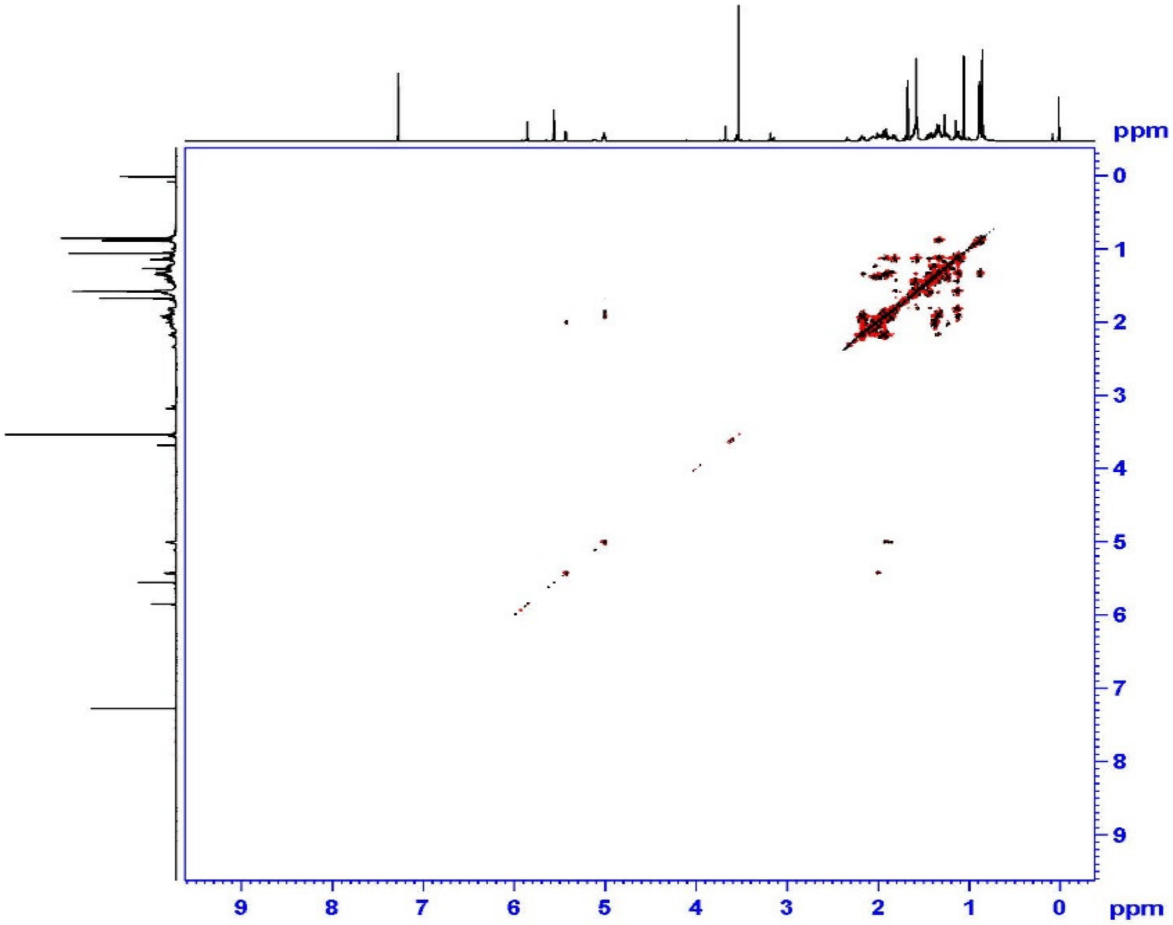

S17 HMBC spectrum of dactylospene B (**2**) in $CDCl_3$.

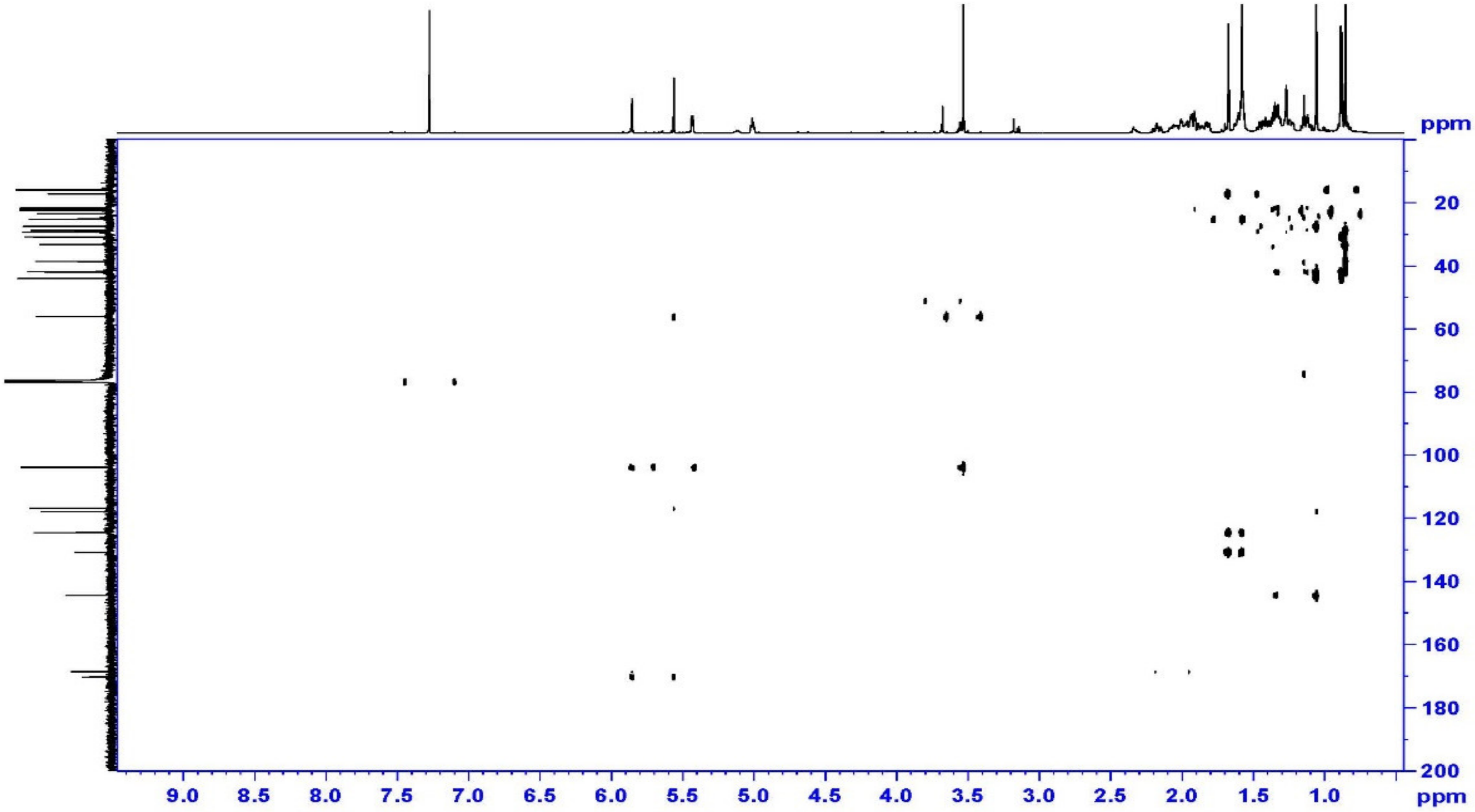

S18 NOESY spectrum of dactylospene B (**2**) in $CDCl_3$.

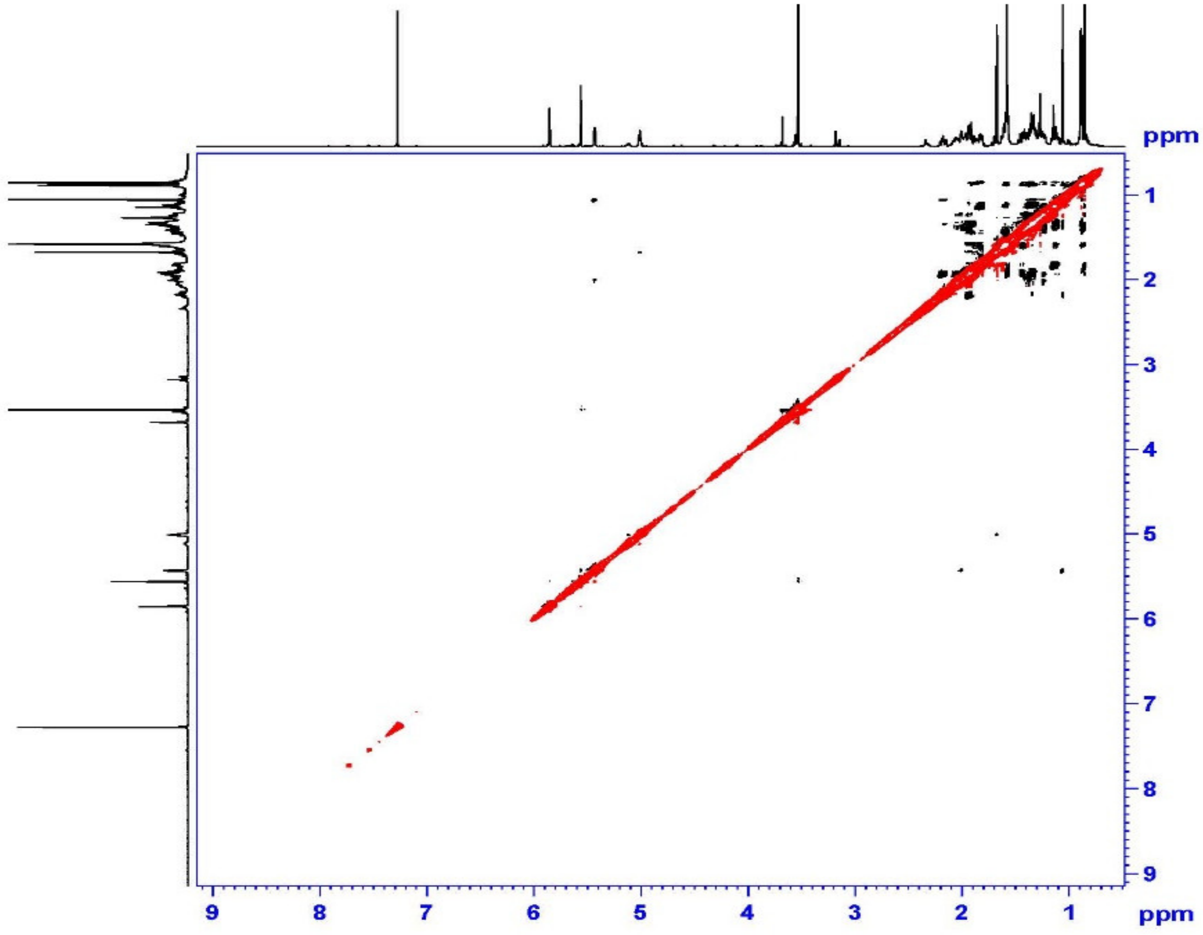

S19 HRESIMS of dactylospene B (**2**).

User Spectra

Fragmentor Voltage	Collision Energy	Ionization Mode
150	0	ESI

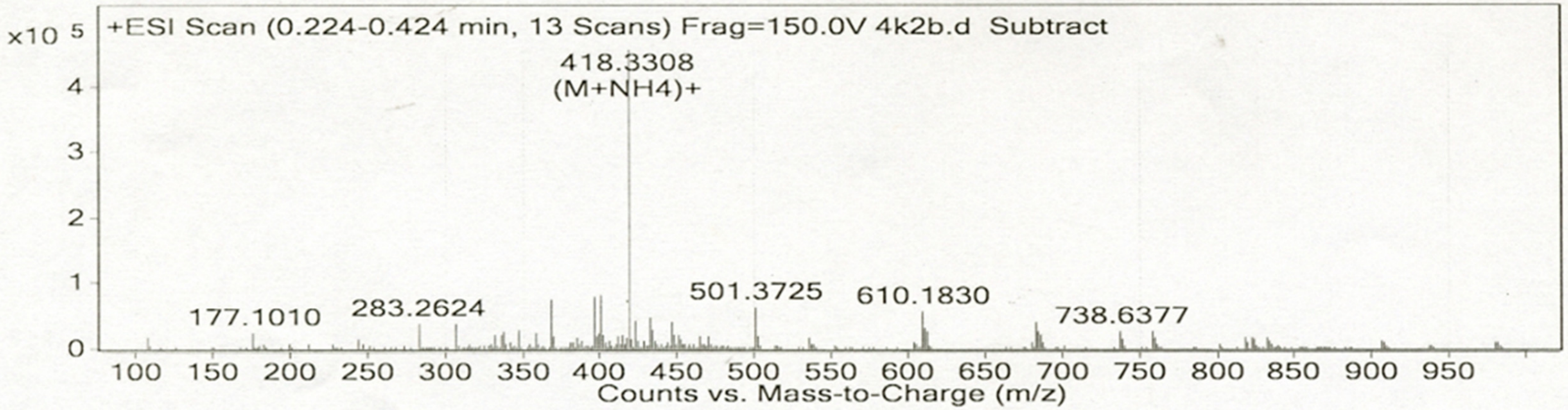

Peak List

m/z	z	Abund	Formula	Ion
369.278		74074.3		
396.3462		80368.6		
401.3037		82179.9		
418.3308	1	455820	C26 H44 N O3	(M+NH4)+
419.334	1	108900.7	C26 H44 N O3	(M+NH4)+
423.2862		42859.7		
432.31		46144.4		
446.2902		40435.3		
501.3725		64291		
610.183	1	57328		

Formula Calculator Results

IonFormula	Measured Mass	Tgt Mass	Diff (ppm)	Score
C26 H44 N O3	418.3308	418.3316	2.03	93.91

S20 UV spectrum of dactylospene B (**2**).

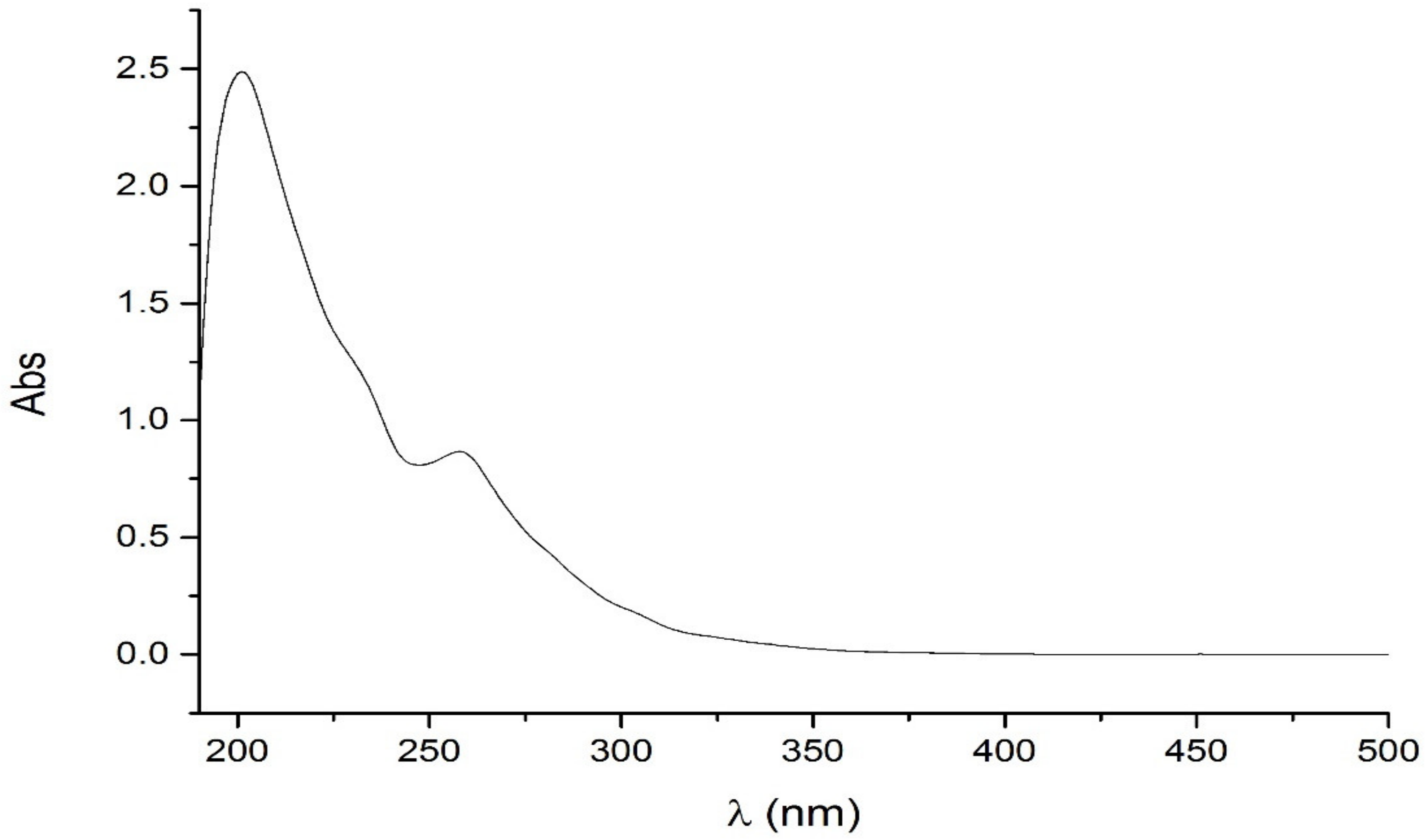

S21 IR spectrum of dactylospene B (**2**).

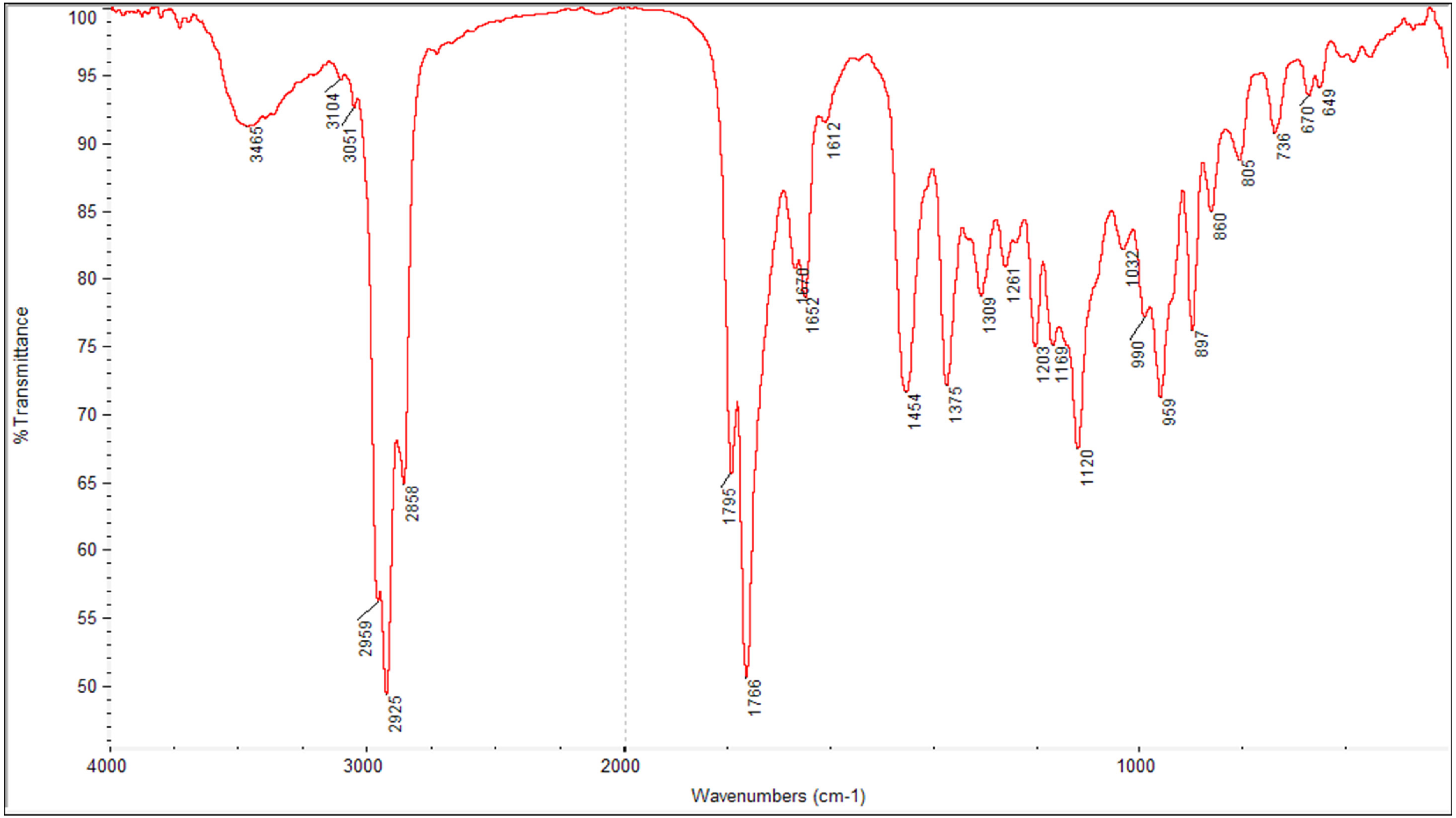

S22 ^{1}H NMR spectrum of dactylospene C (**3**) in $CDCl_3$.

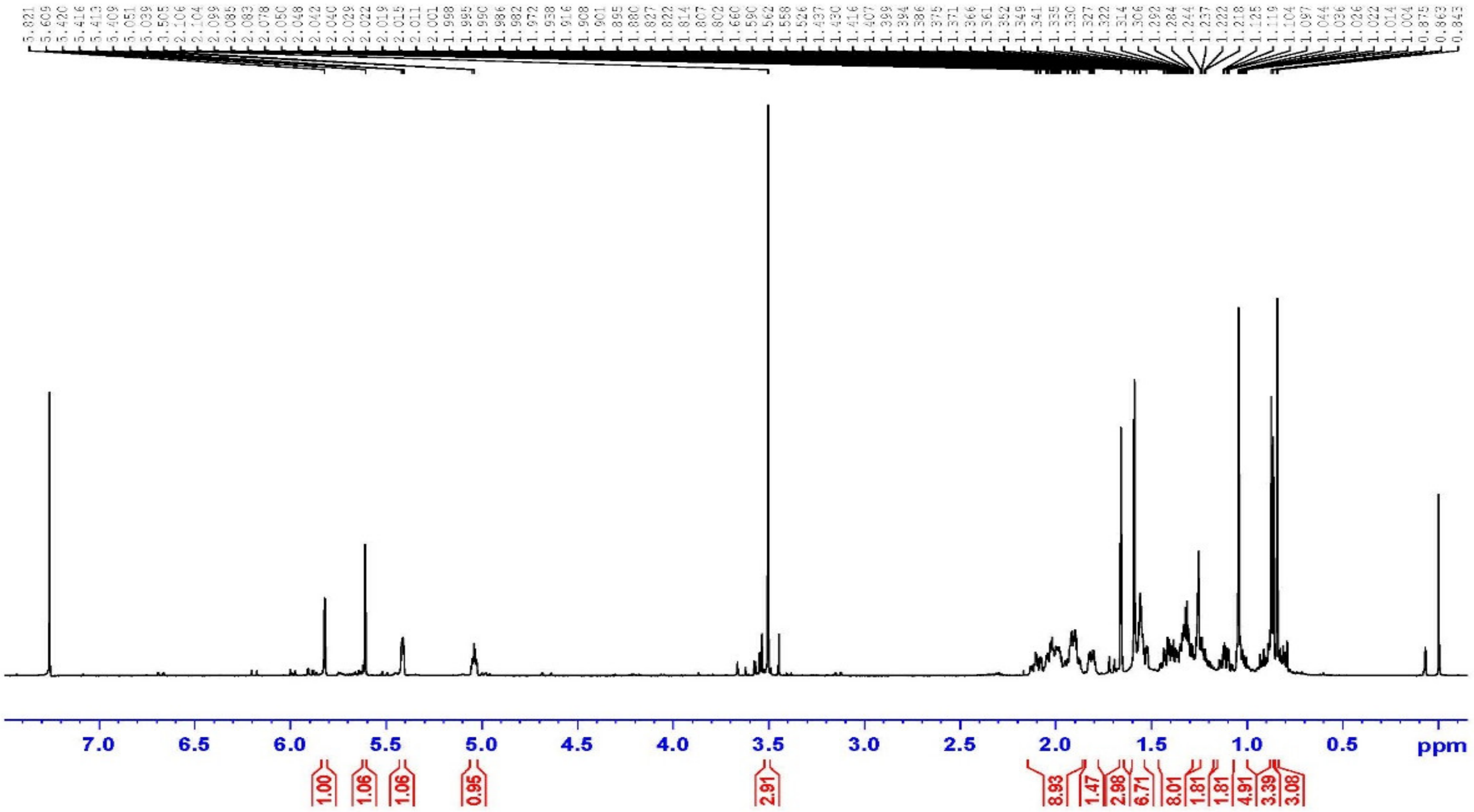

S23 ^{13}C NMR spectrum of dactylospene C (**3**) in $CDCl_3$.

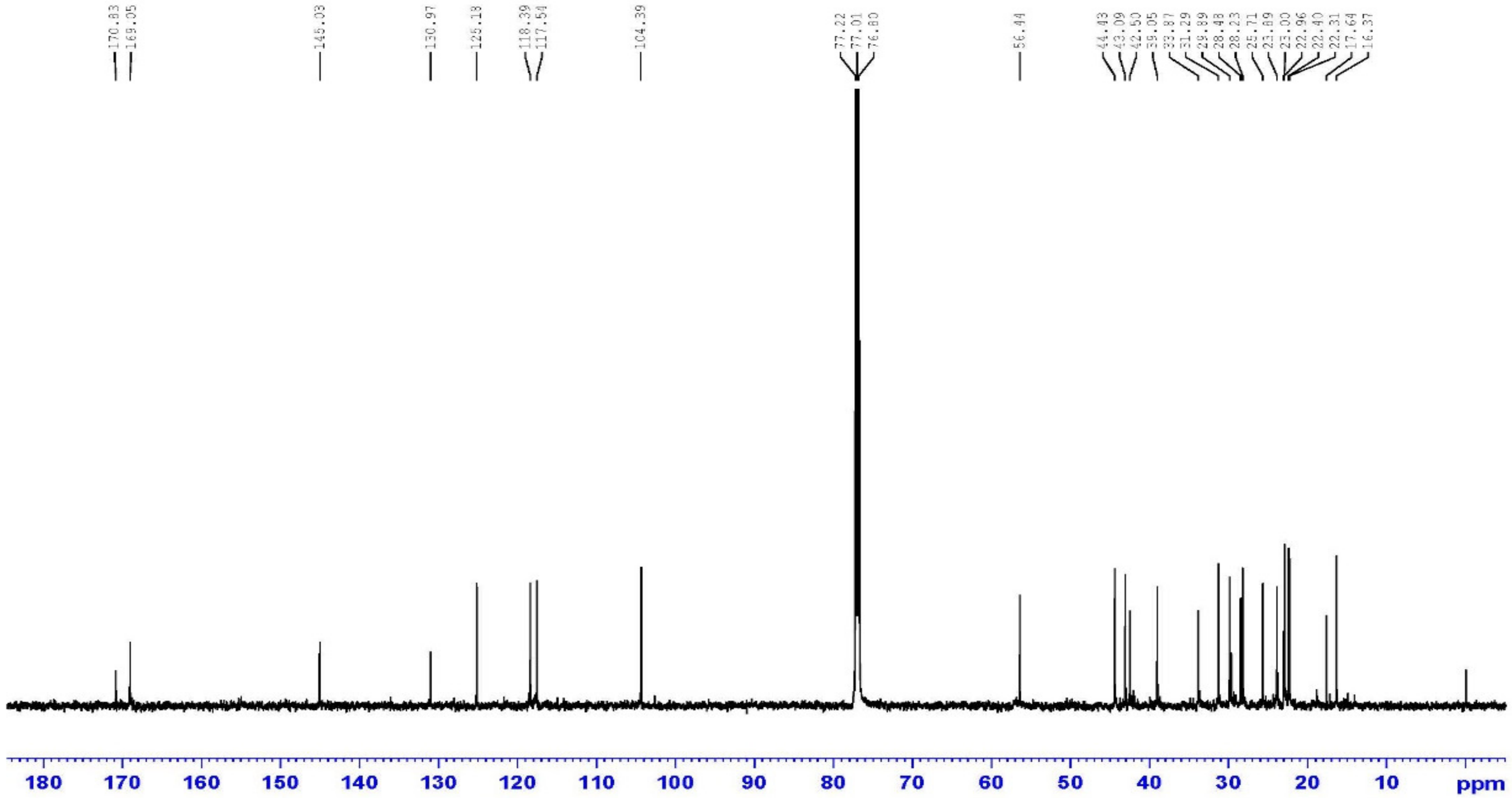

S24 DEPT135 spectrum of dactylospene C (**3**) in $CDCl_3$.

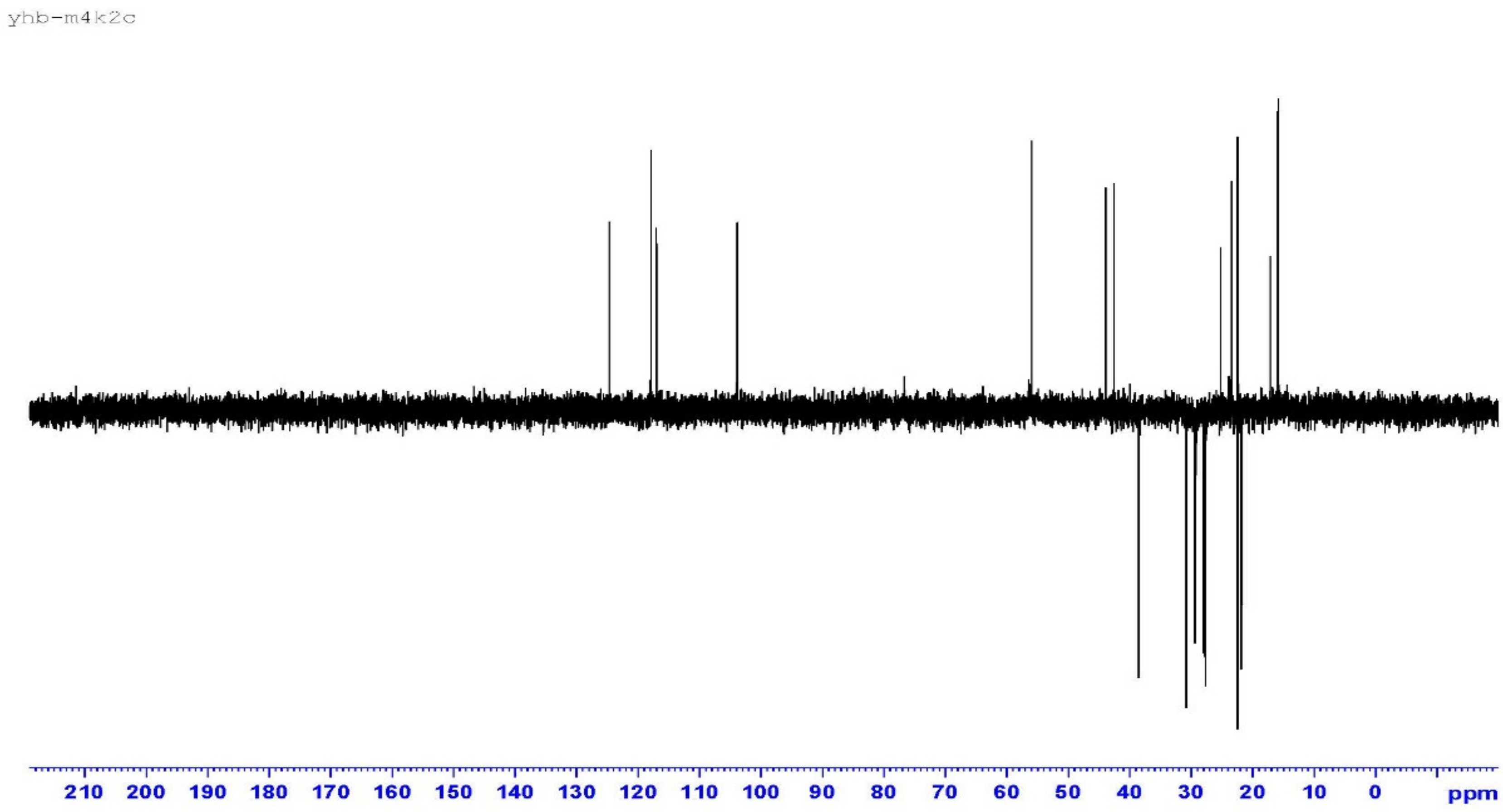

S25 HSQC spectrum of dactylospene C (**3**) in $CDCl_3$.

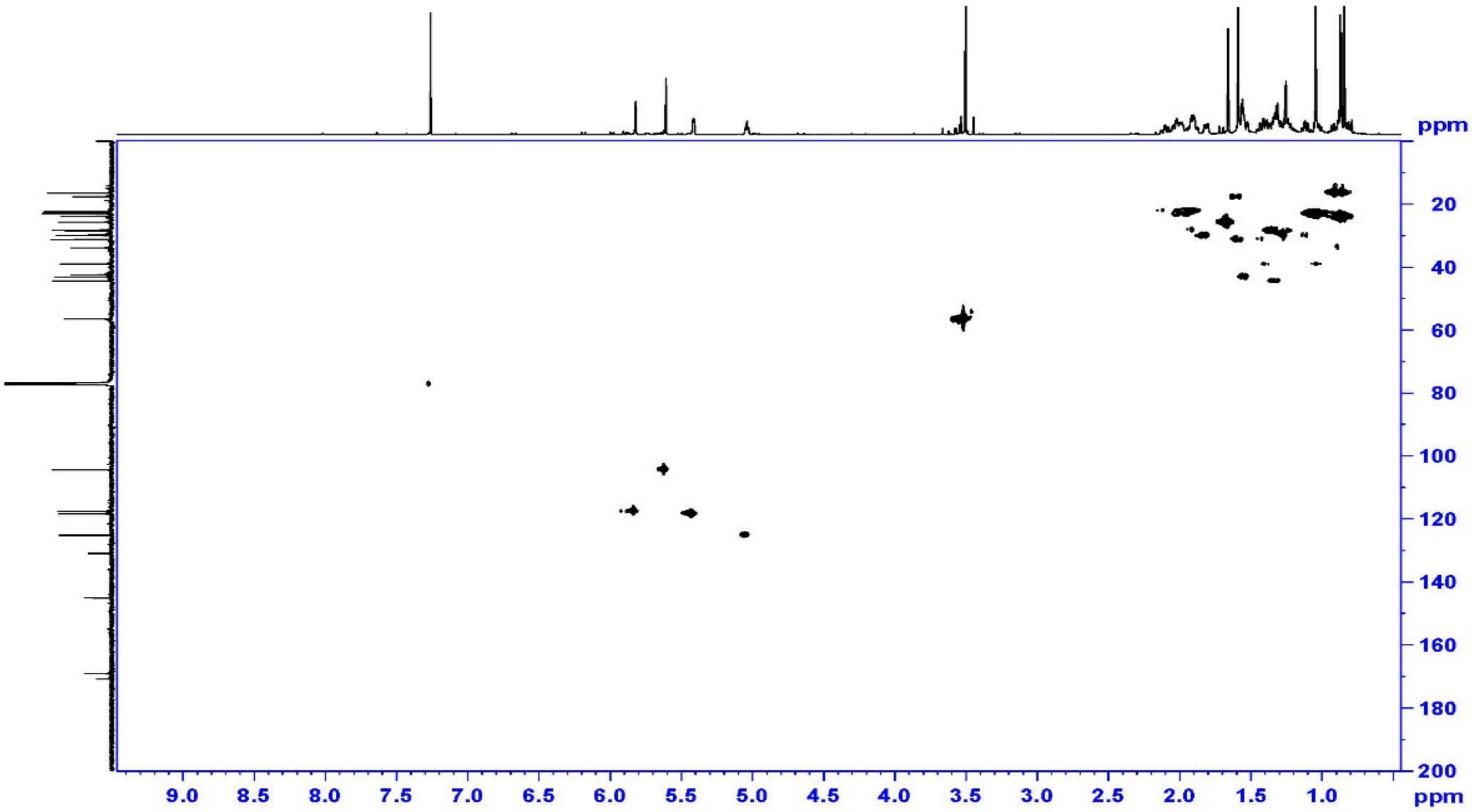

S26 COSY spectrum of dactylospene C (**3**) in $CDCl_3$.

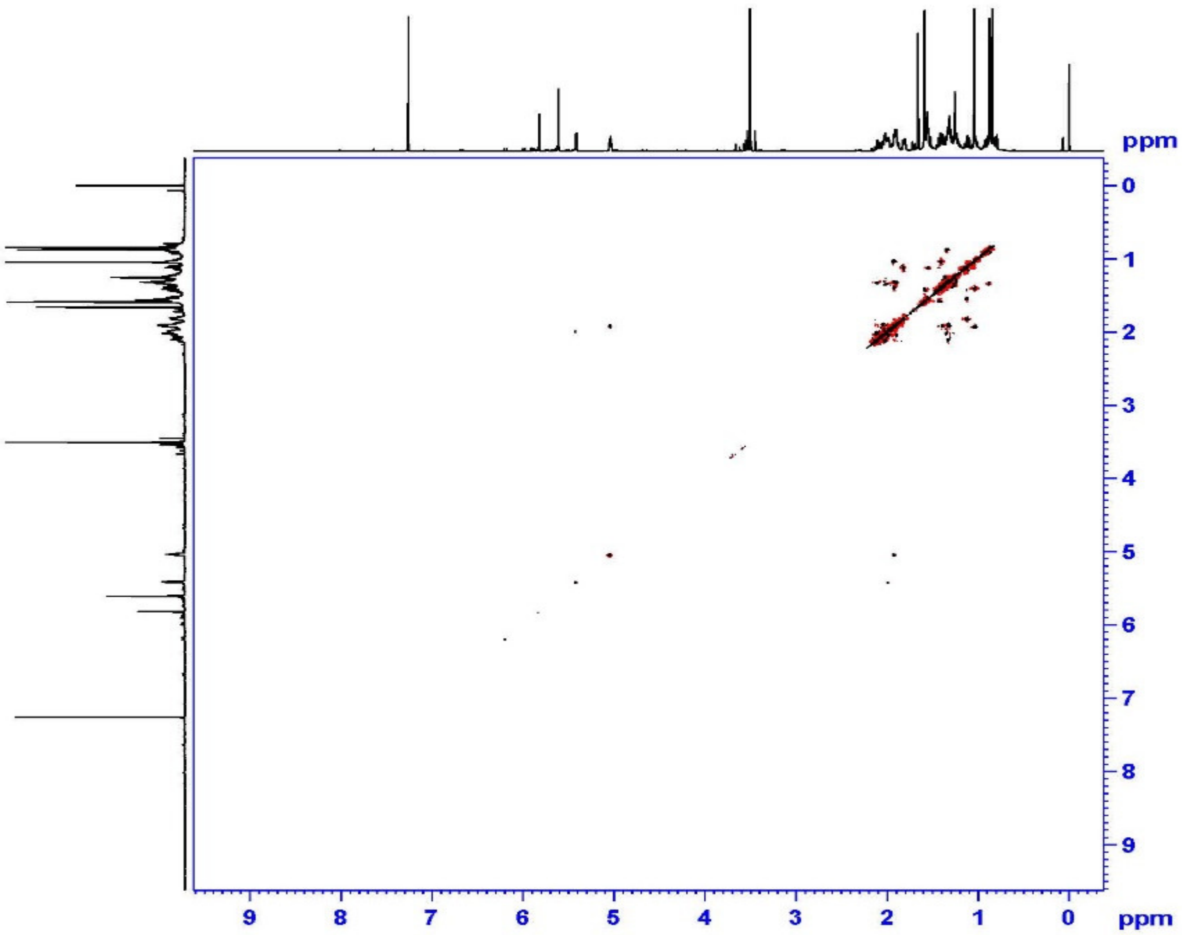

S27 HMBC spectrum of dactylospene C (**3**) in $CDCl_3$.

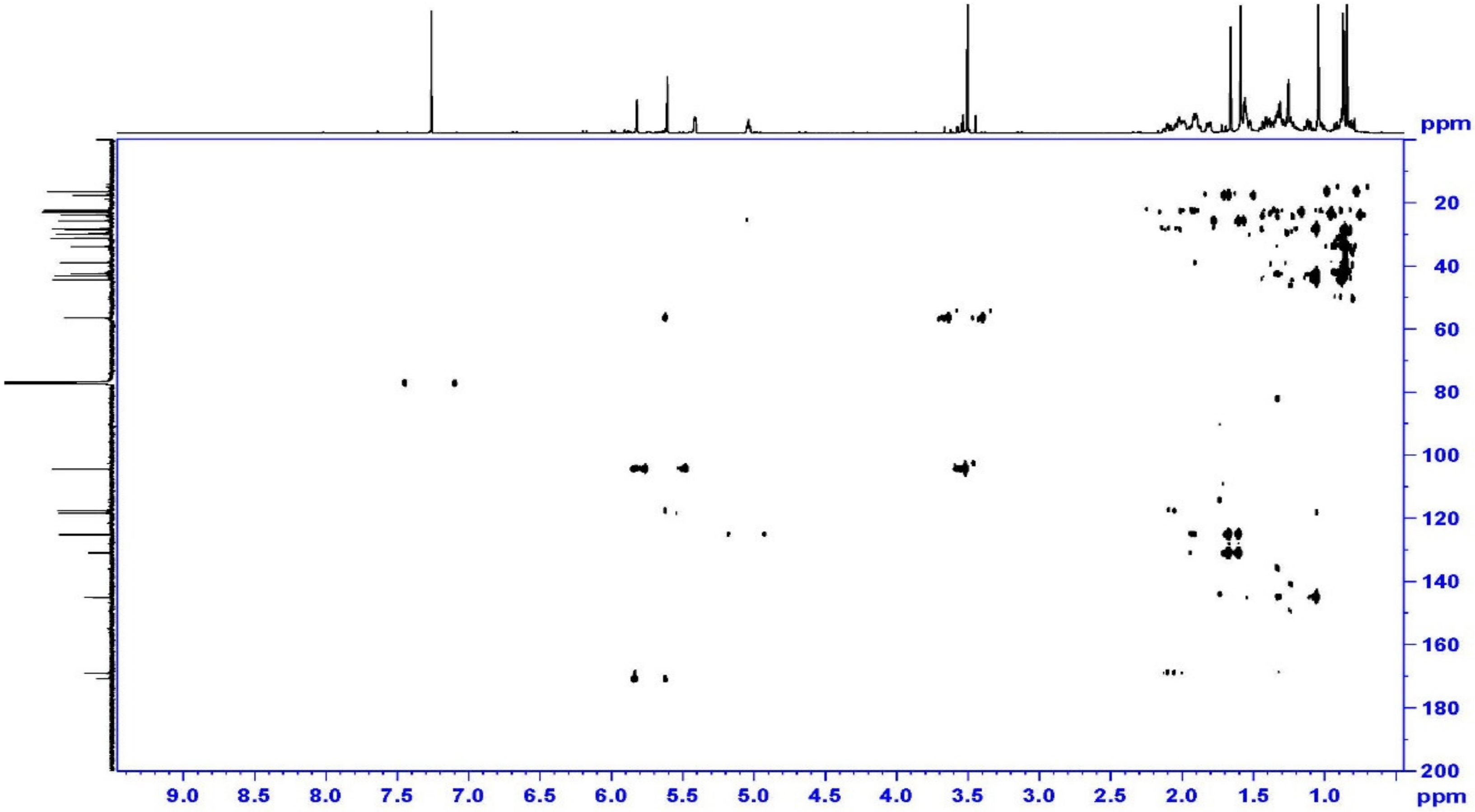

S28 NOESY spectrum of dactylospene C (**3**) in $CDCl_3$.

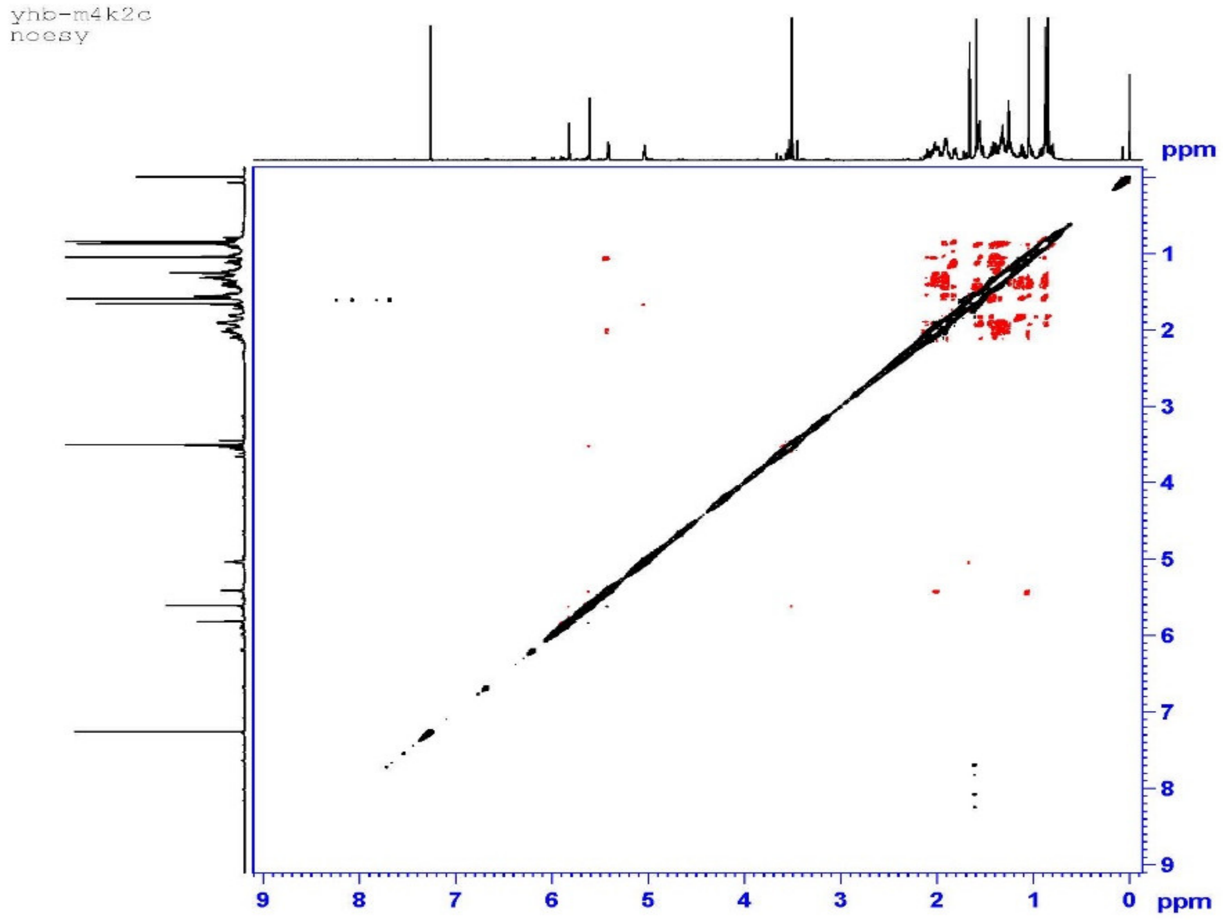

S29 HRESIMS of dactylospene C (**3**).

User Spectra

Fragmentor Voltage	Collision Energy	Ionization Mode
150	0	ESI

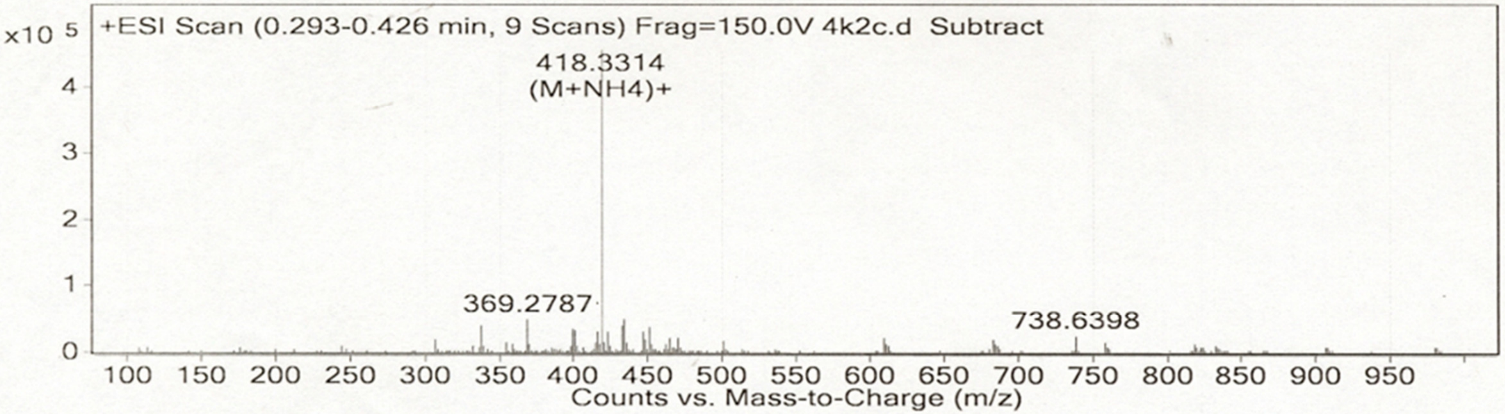

Peak List

m/z	z	Abund	Formula	Ion
338.3416		39652		
369.2787		49758.4		
399.2894		34535.5		
401.3047		31960.8		
418.3314	1	455710.8	C26 H44 N O3	(M+NH4)+
419.3348	1	107031.8	C26 H44 N O3	(M+NH4)+
423.2869		30984.6		
432.3111		40071.7		
434.3262		49656.7		
450.3211		38520.8		

Formula Calculator Results

IonFormula	Measured Mass	Tgt Mass	Diff (ppm)	Score
C26 H44 N O3	418.3314	418.3316	0.5	94.68

S30 UV spectrum of dactylospene C (**3**).

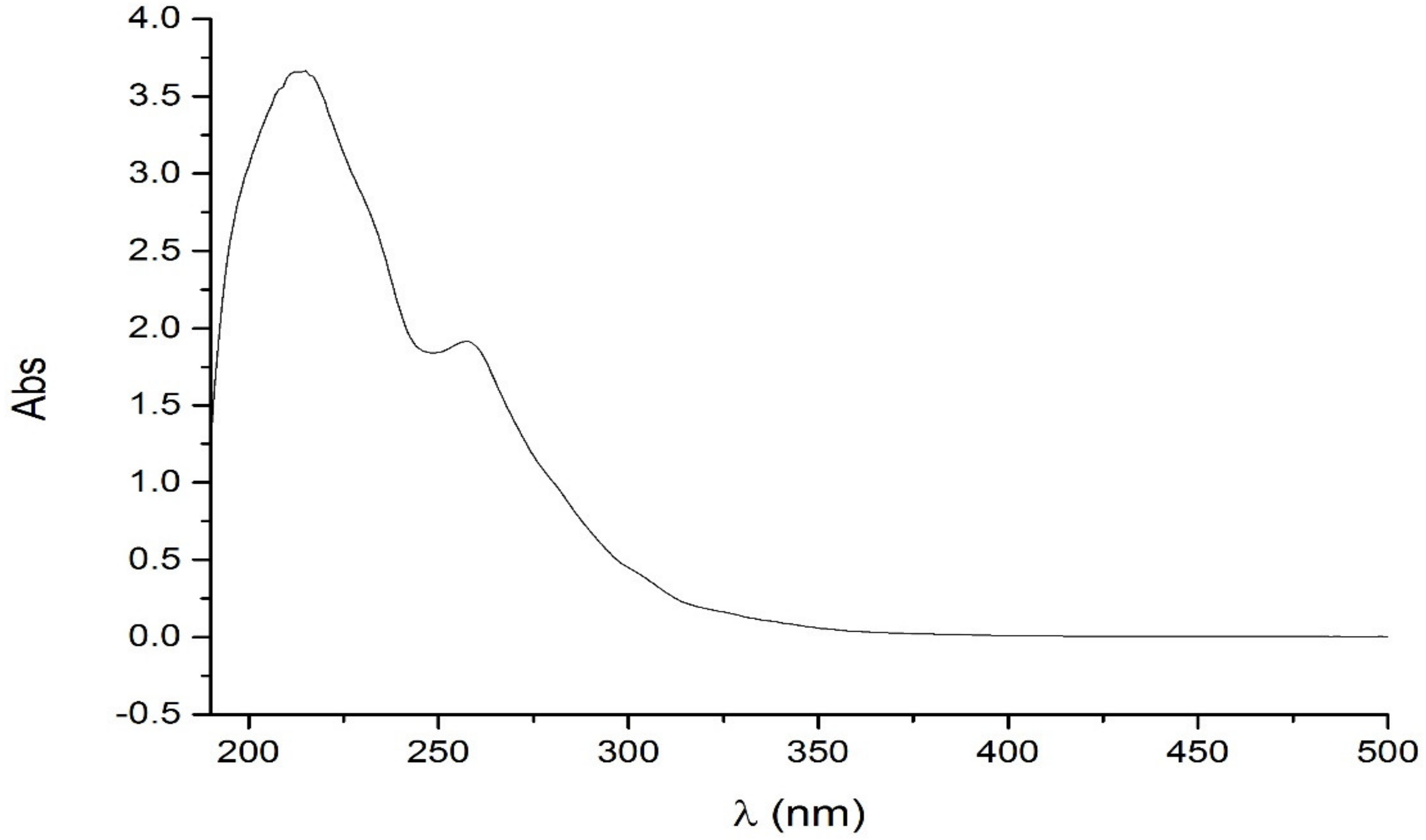

S31 IR spectrum of dactylospene C (**3**).

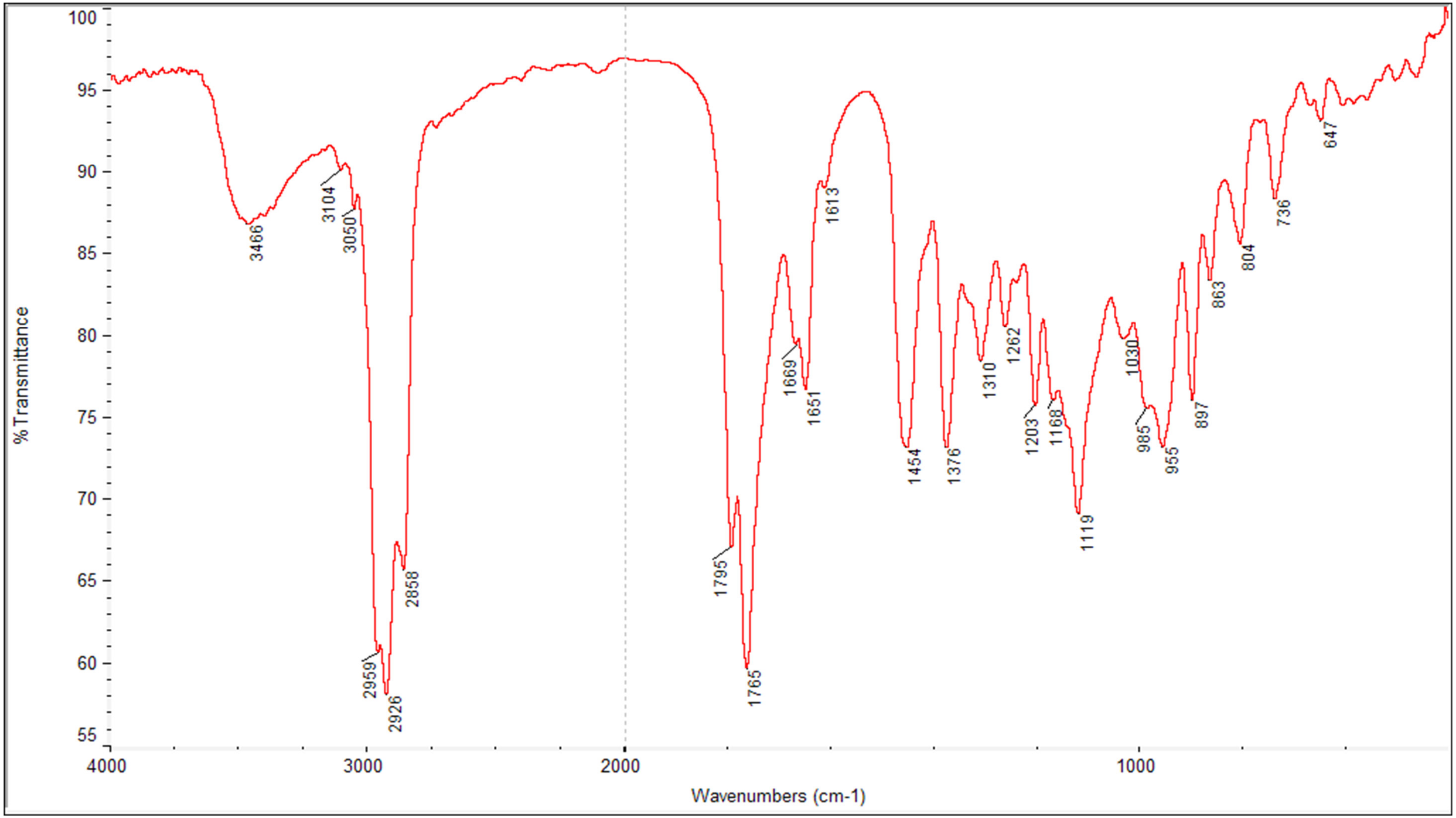

S32 ^{1}H NMR spectrum of dactylospene D (**4**) in $CDCl_3$.

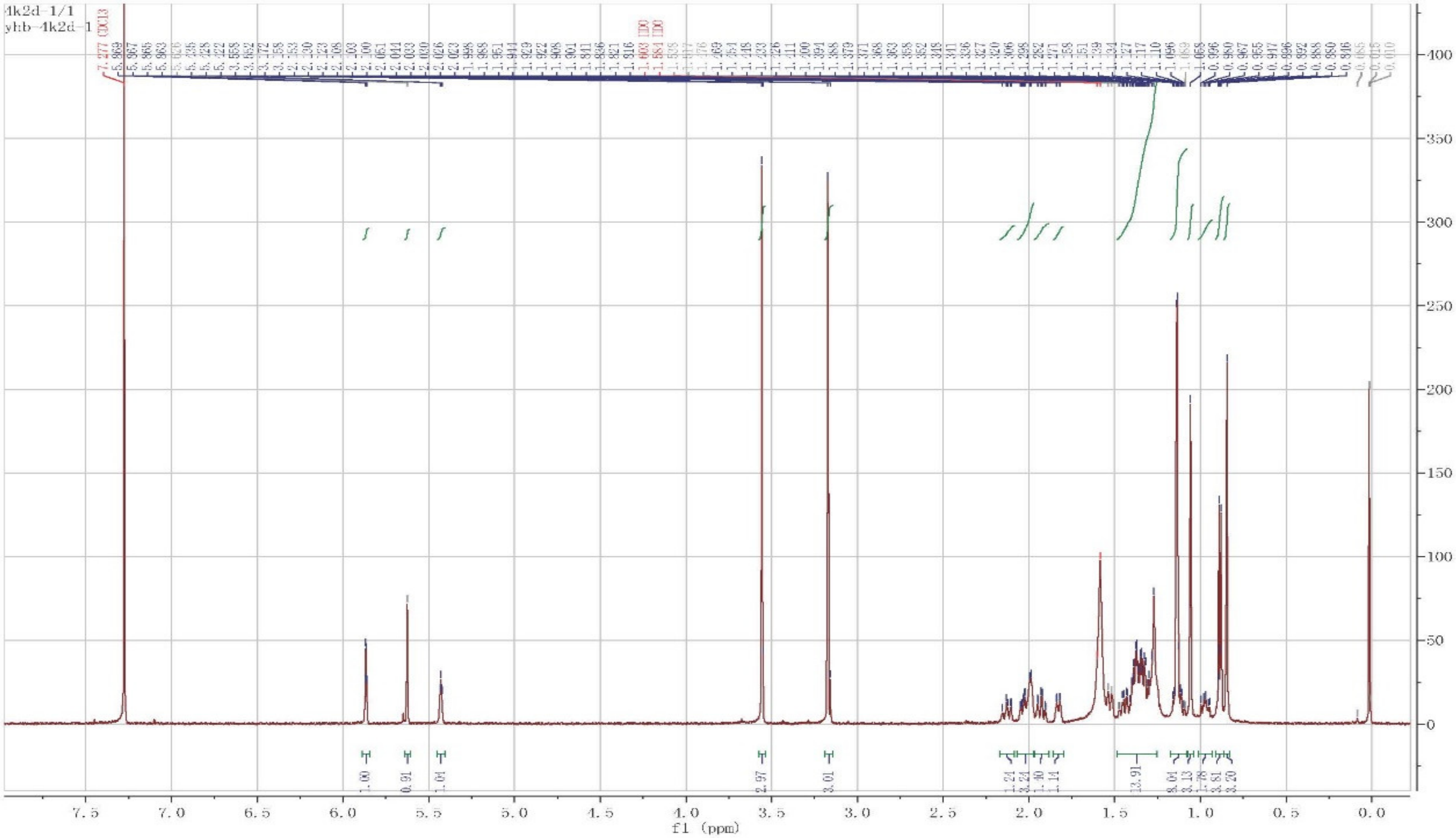

S33 ^{13}C NMR spectrum of dactylospene D (**4**) in $CDCl_3$.

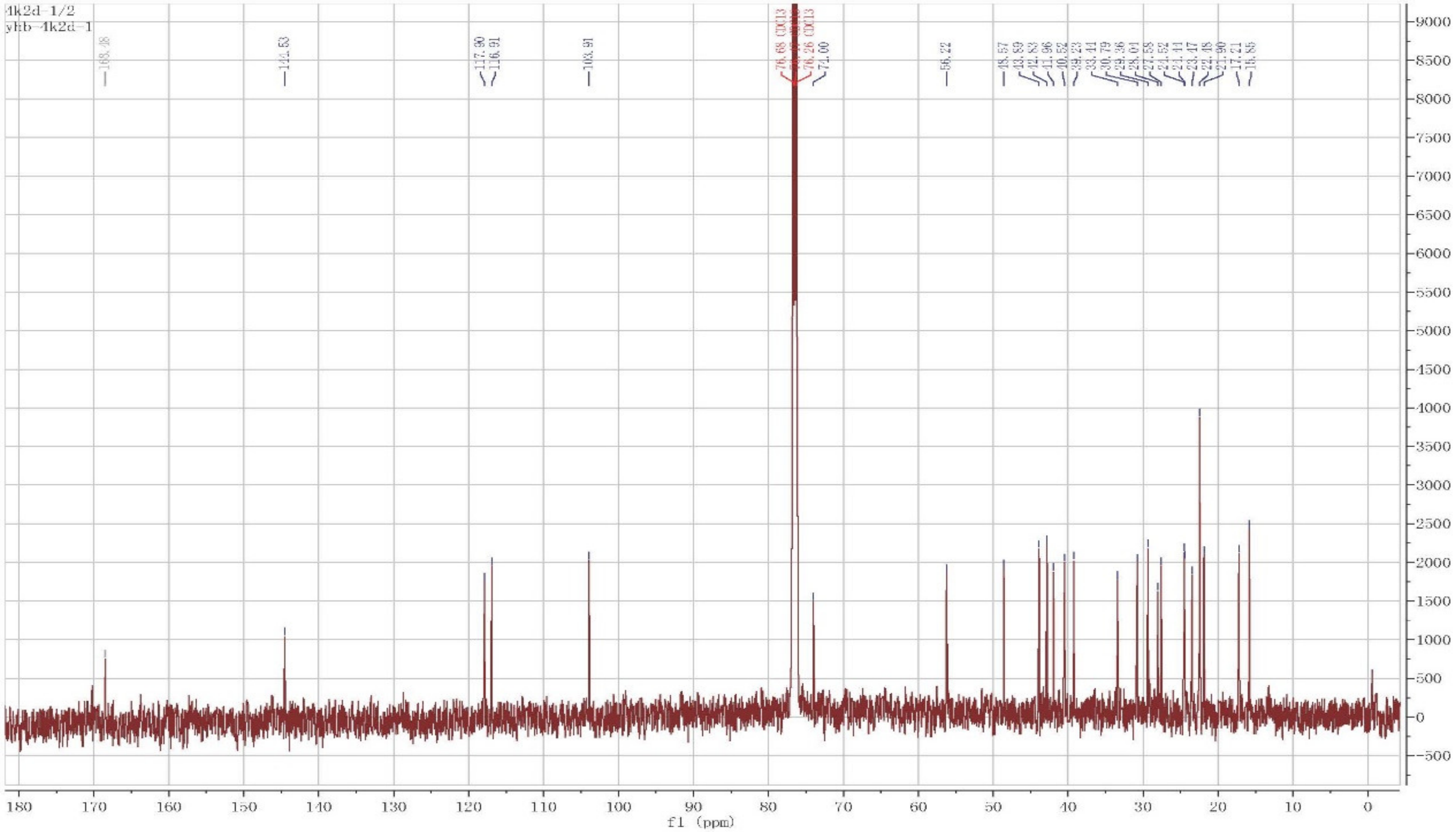

S34 DEPT135 spectrum of dactylospene D (**4**) in $CDCl_3$.

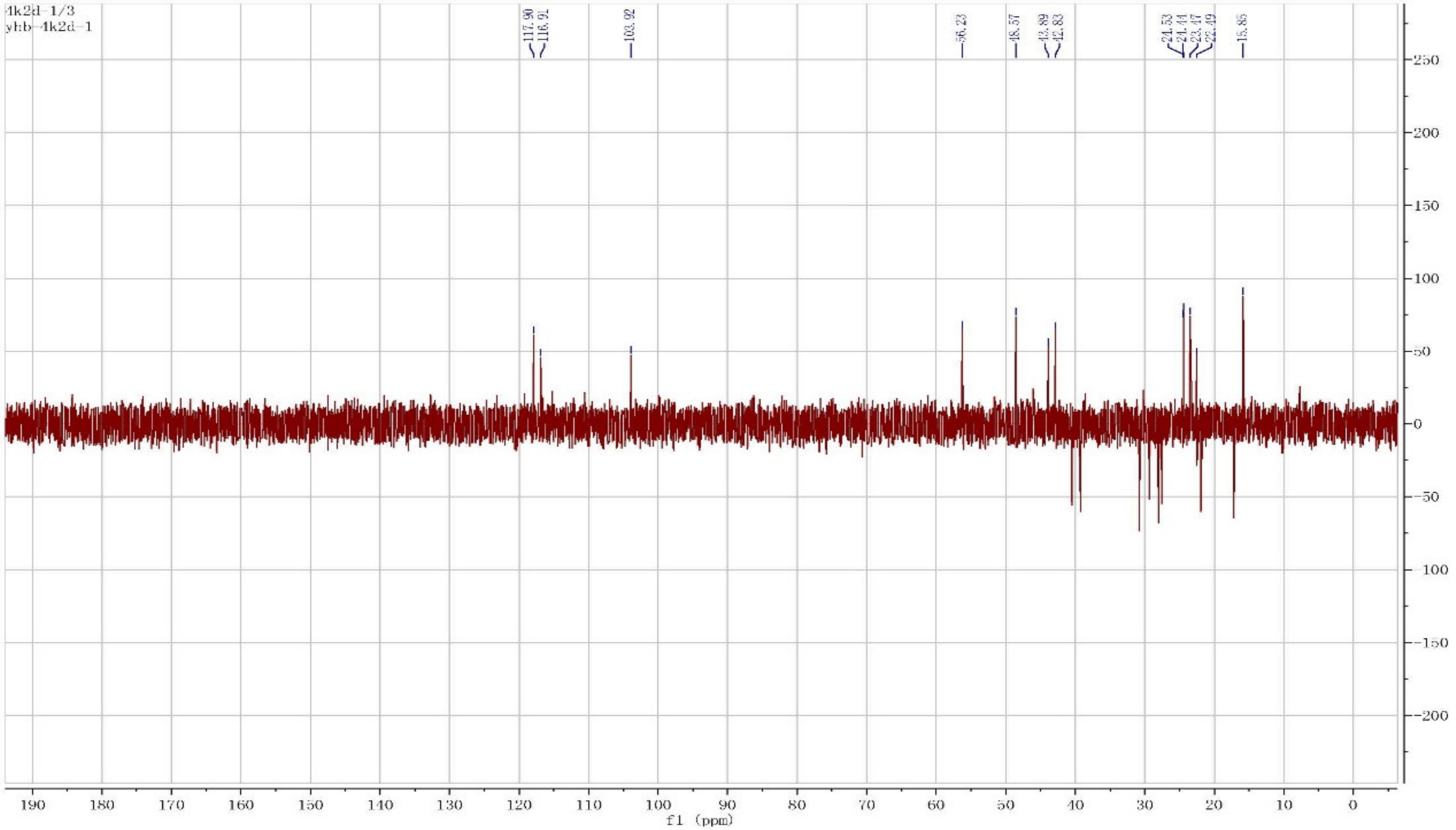

S35 HSQC spectrum of dactylospene D (**4**) in $CDCl_3$.

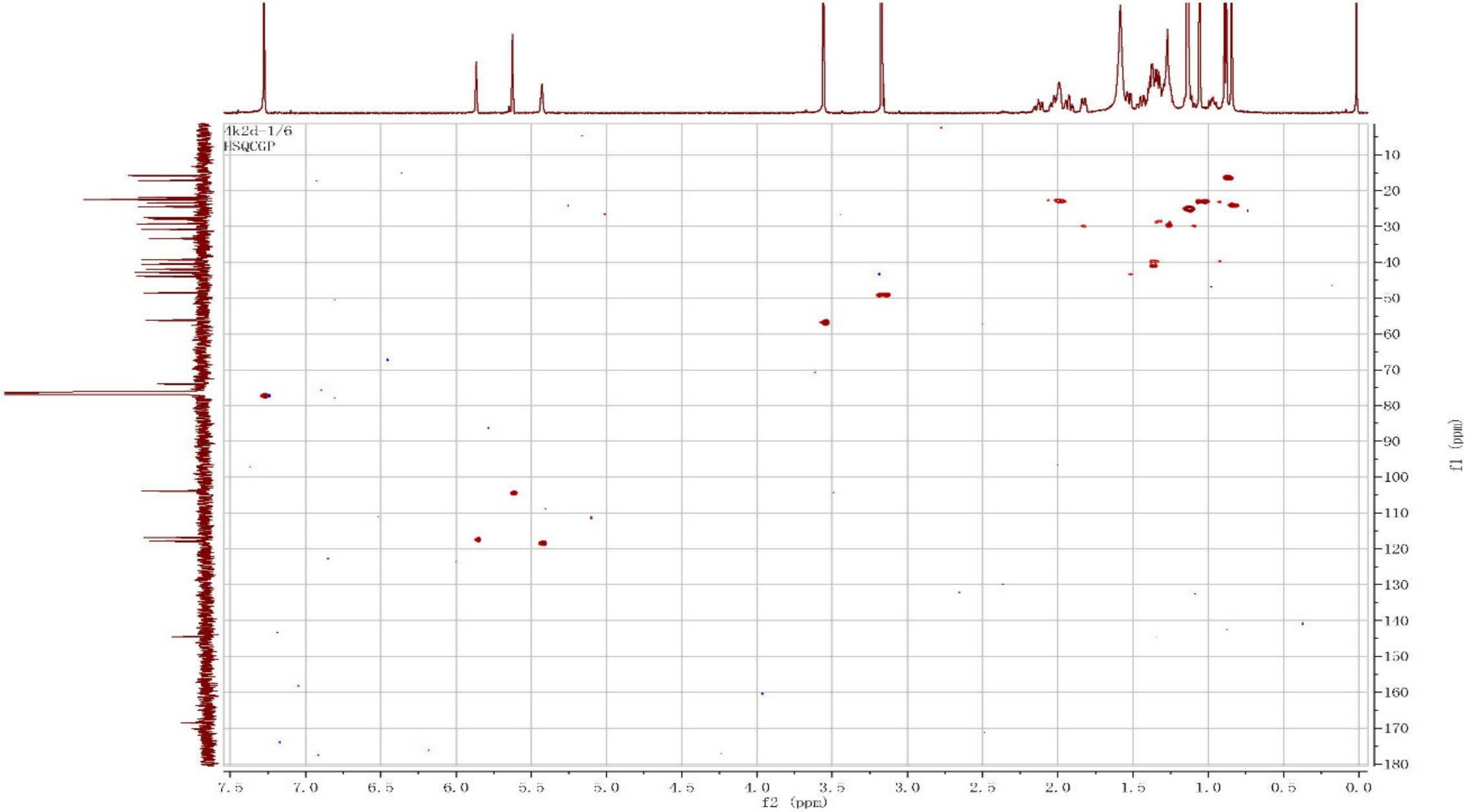

S36 COSY spectrum of dactylospene D (**4**) in $CDCl_3$.

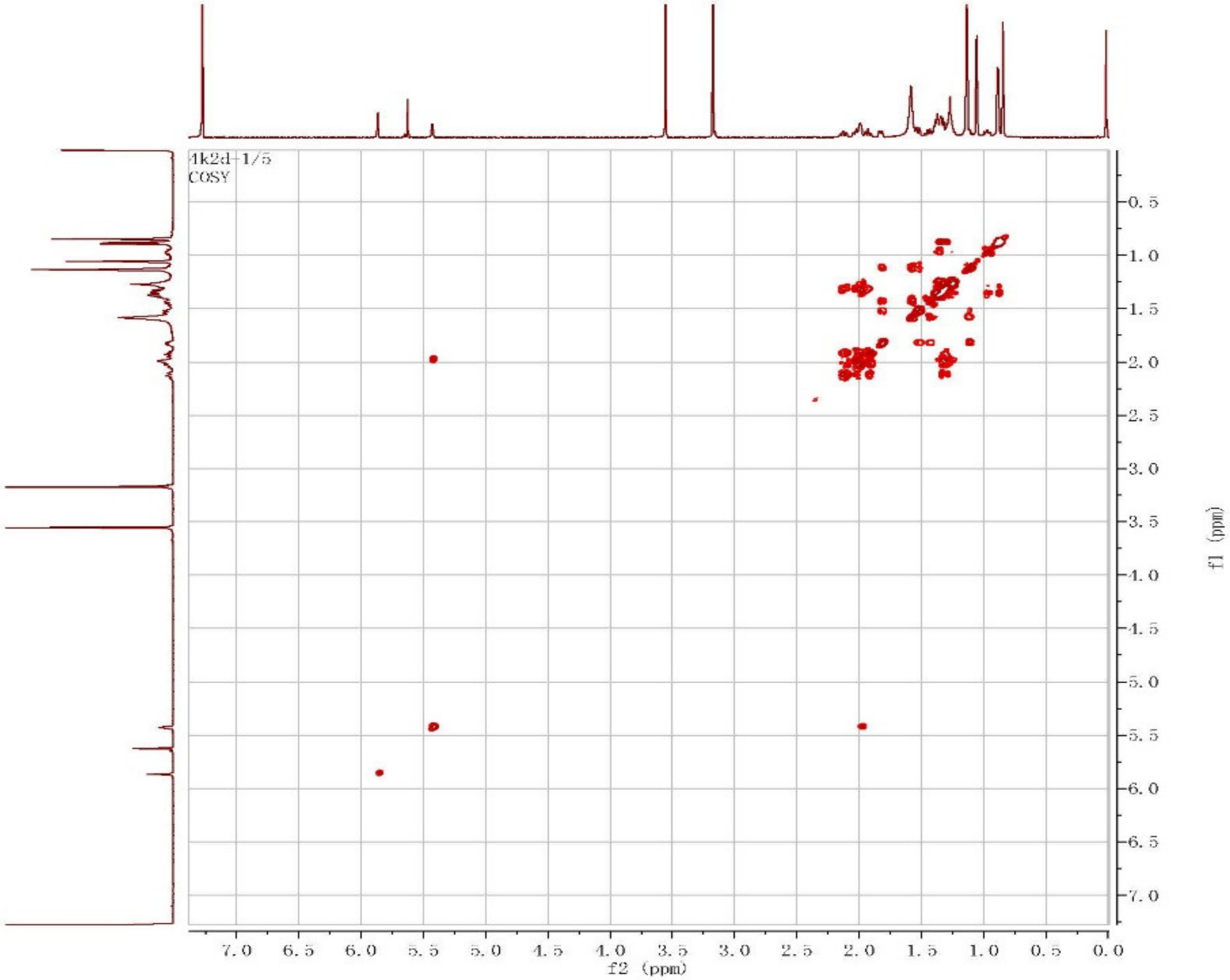

S37 HMBC spectrum of dactylospene D (**4**) in $CDCl_3$.

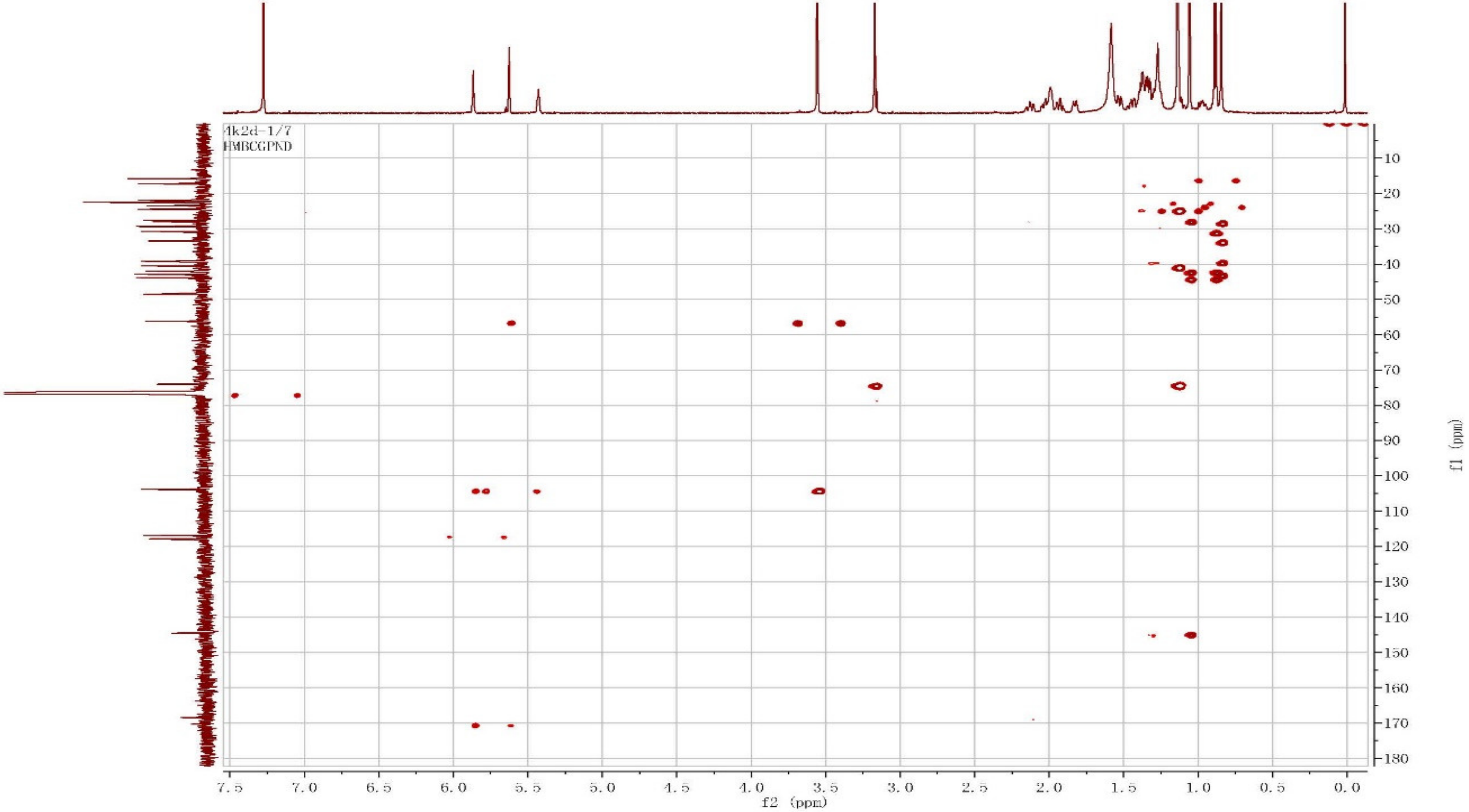

S38 NOESY spectrum of dactylospene D (**4**) in $CDCl_3$.

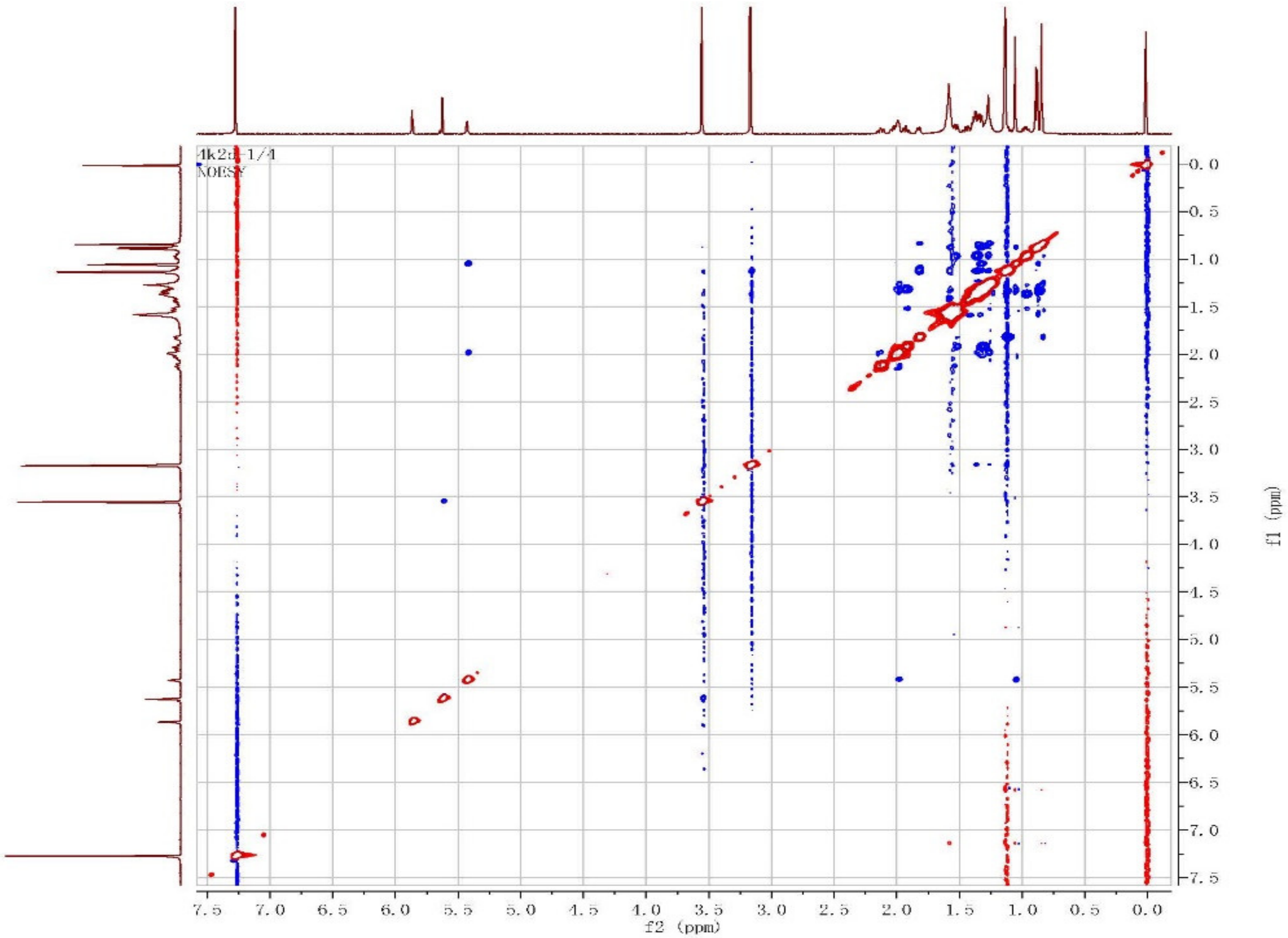

S39 HRESIMS of dactylospene D (**4**).

User Spectra

Fragmentor Voltage	Collision Energy	Ionization Mode
150	0	ESI

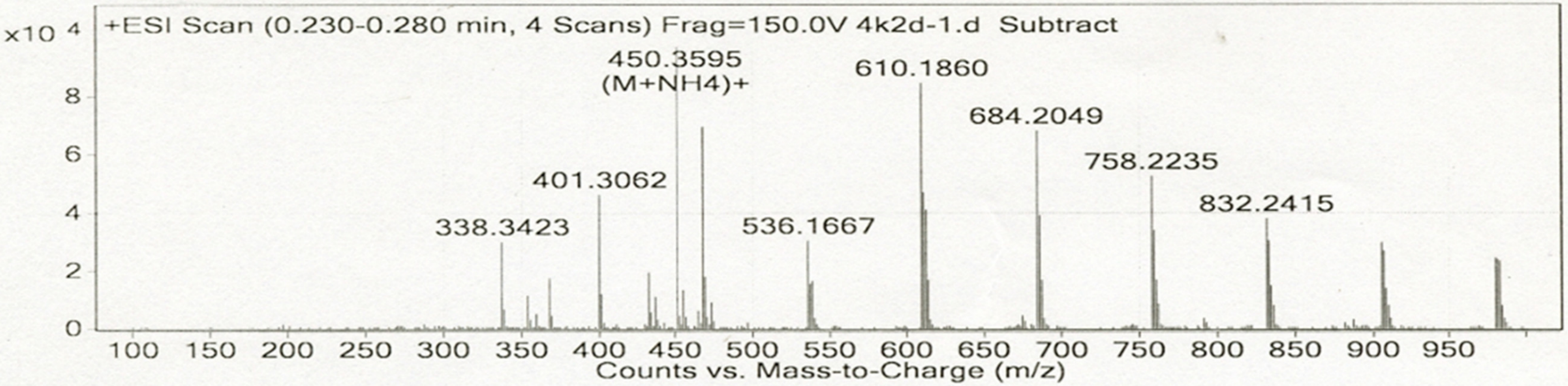

Peak List

m/z	z	Abund	Formula	Ion
401.3062	1	46204.3		
450.3595	1	97112.7	C27 H48 N O4	(M+NH4)+
468.2031	1	69678.9		
610.186	1	84722.9		
611.1865	1	46847.1		
612.1813	1	41176.7		
684.2049	1	68083.2		
685.205	1	44673.6		
686.2011	1	39216.8		
758.2235	1	52492		

Formula Calculator Results

IonFormula	Measured Mass	Tgt Mass	Diff (ppm)	Score
C27 H48 N O4	450.3595	450.3578	-3.99	90.05

S40 UV spectrum of dactylospene D (**4**).

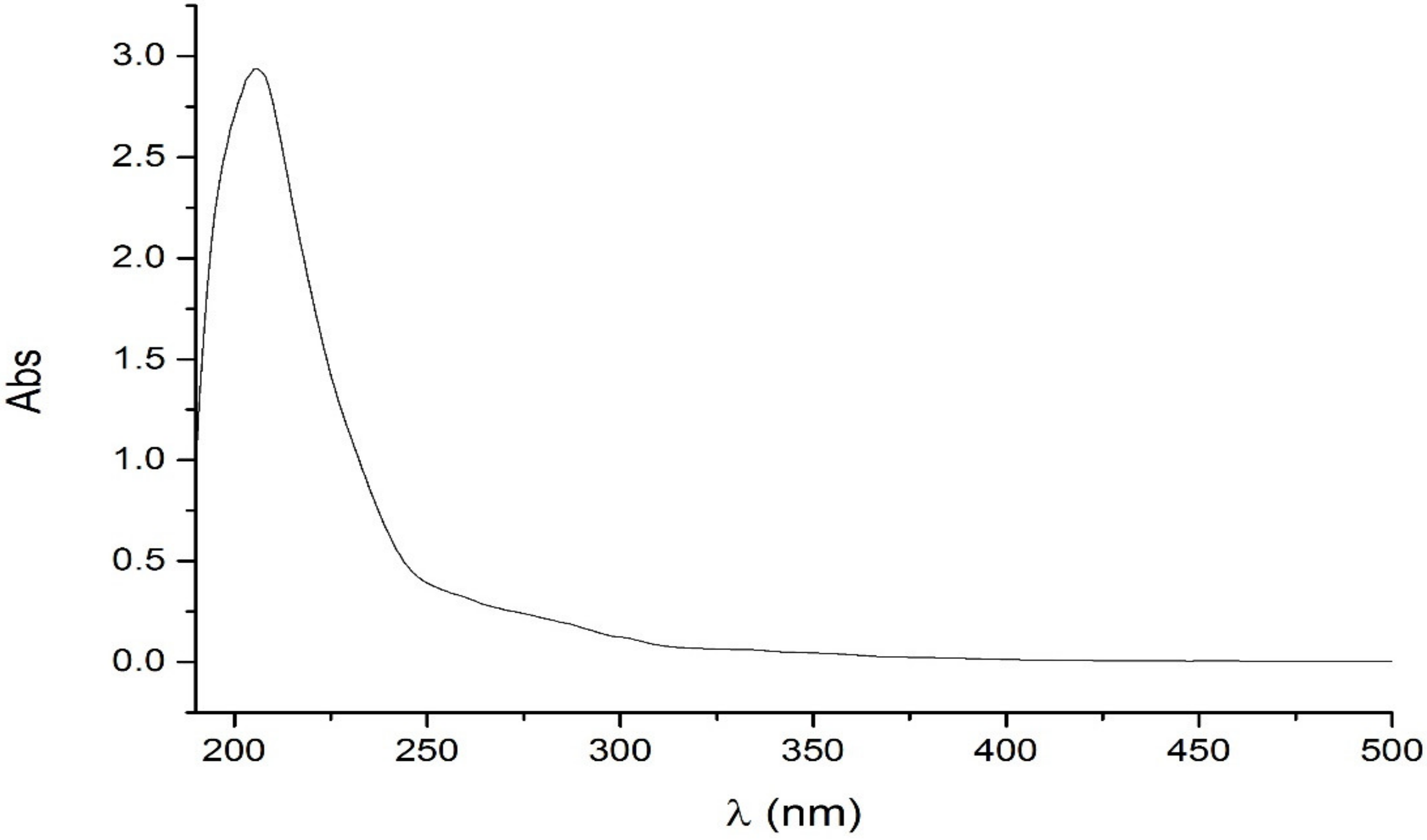

S41 IR spectrum of dactylospene D (**4**).

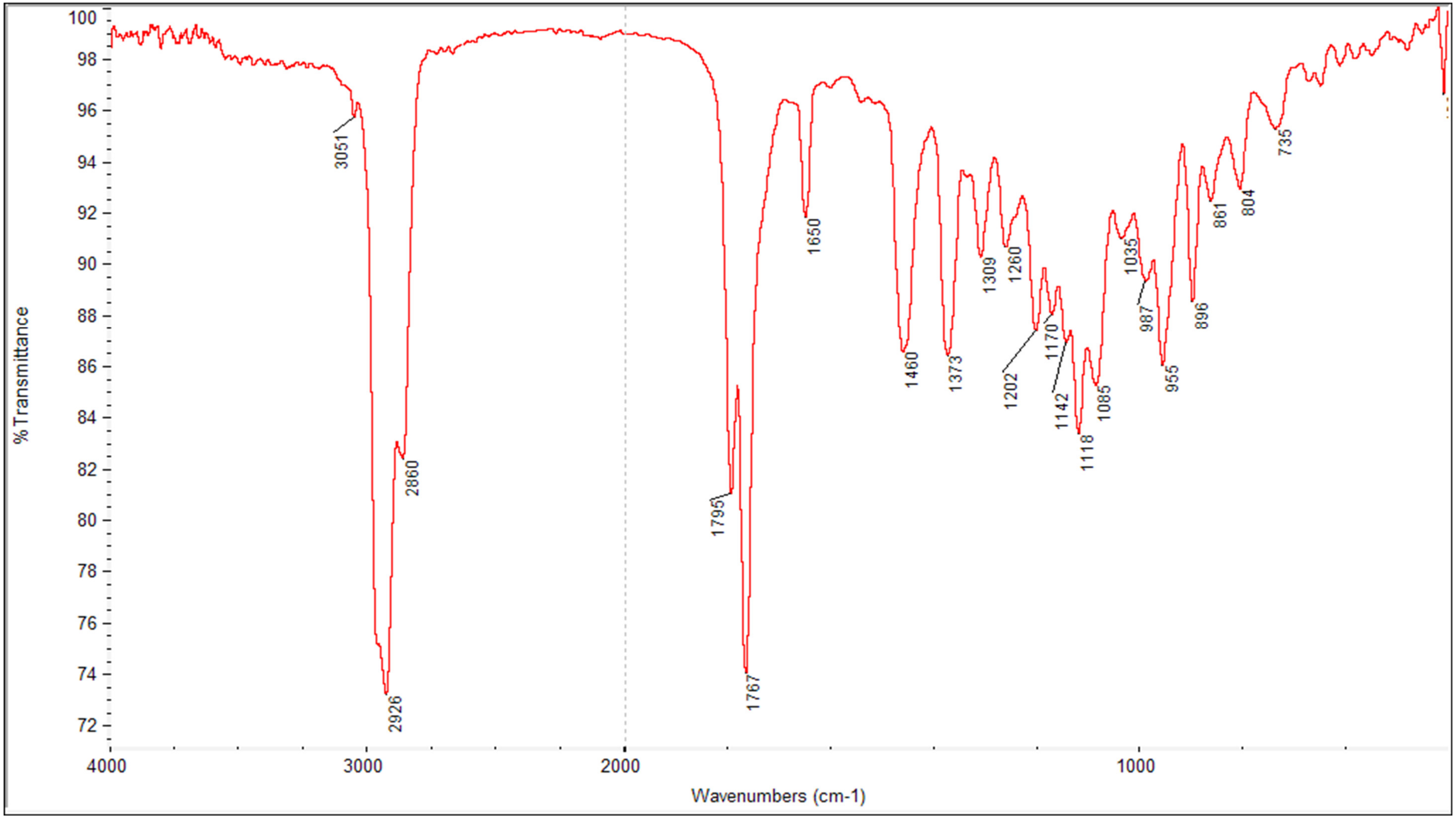

S42 ^{1}H NMR spectrum of dactylospene E (**5**) in $CDCl_3$.

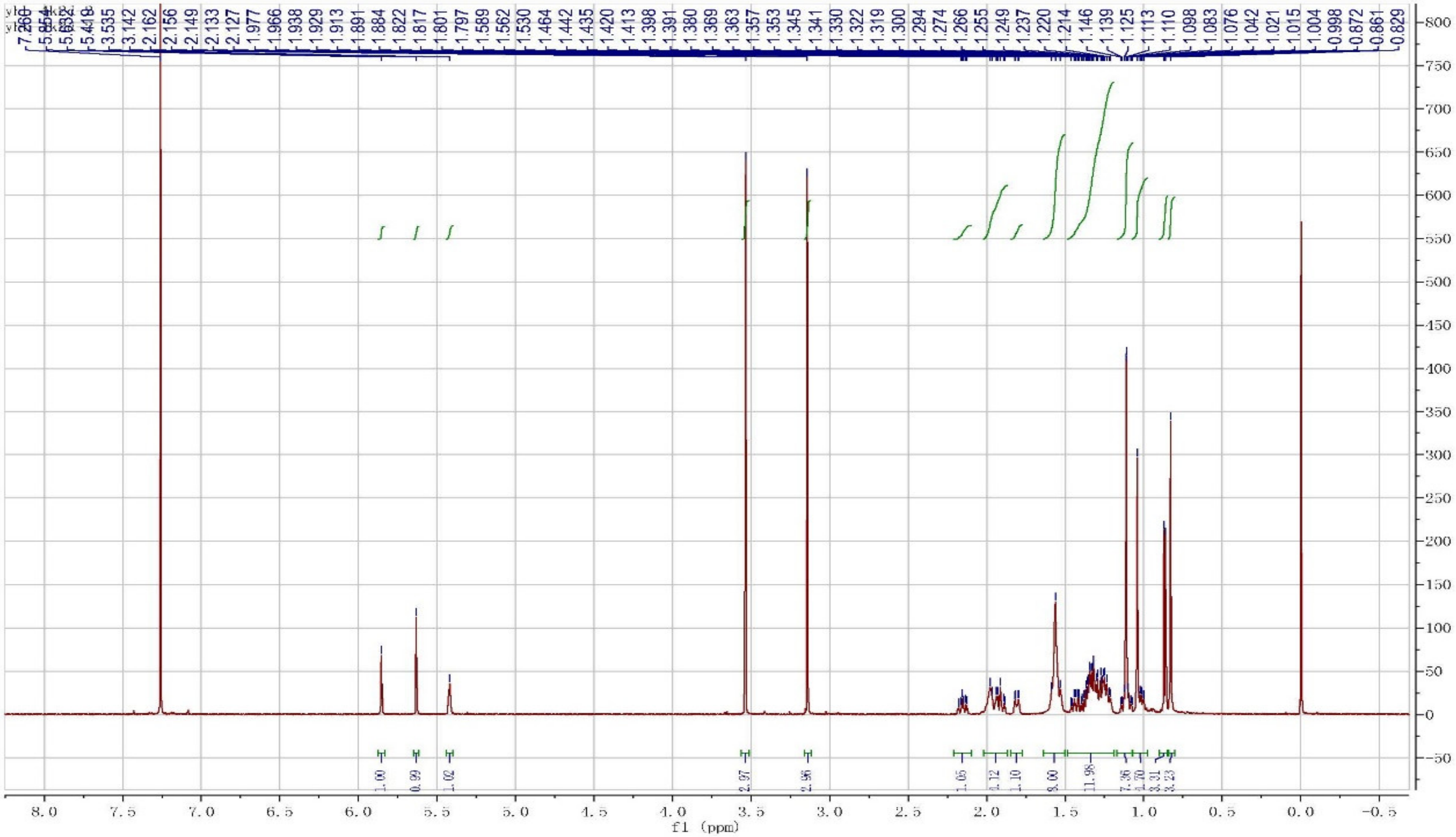

S43 ^{13}C NMR spectrum of dactylospene E (**5**) in $CDCl_3$.

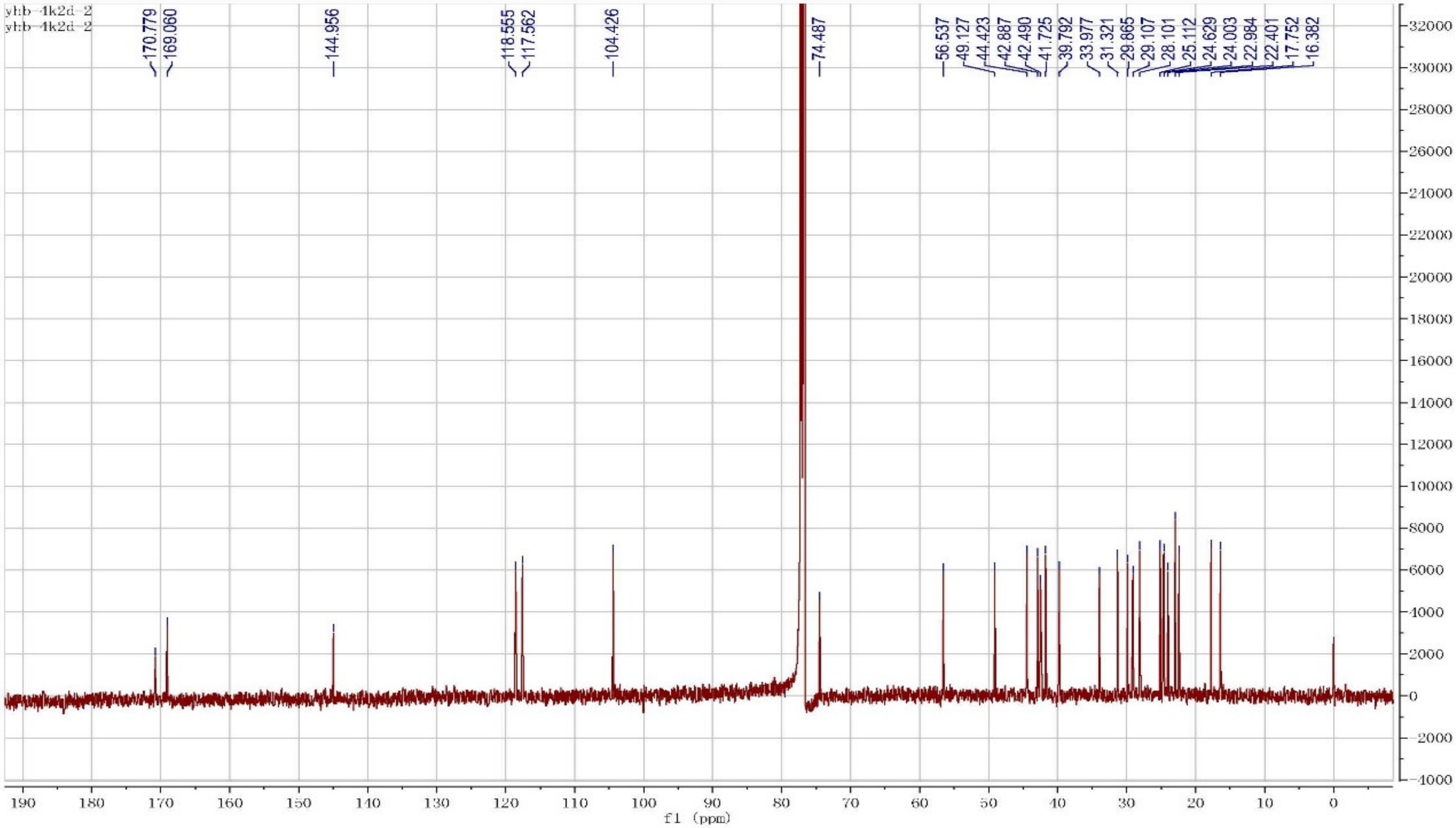

S44 DEPT135 spectrum of dactylospene E (**5**) in $CDCl_3$.

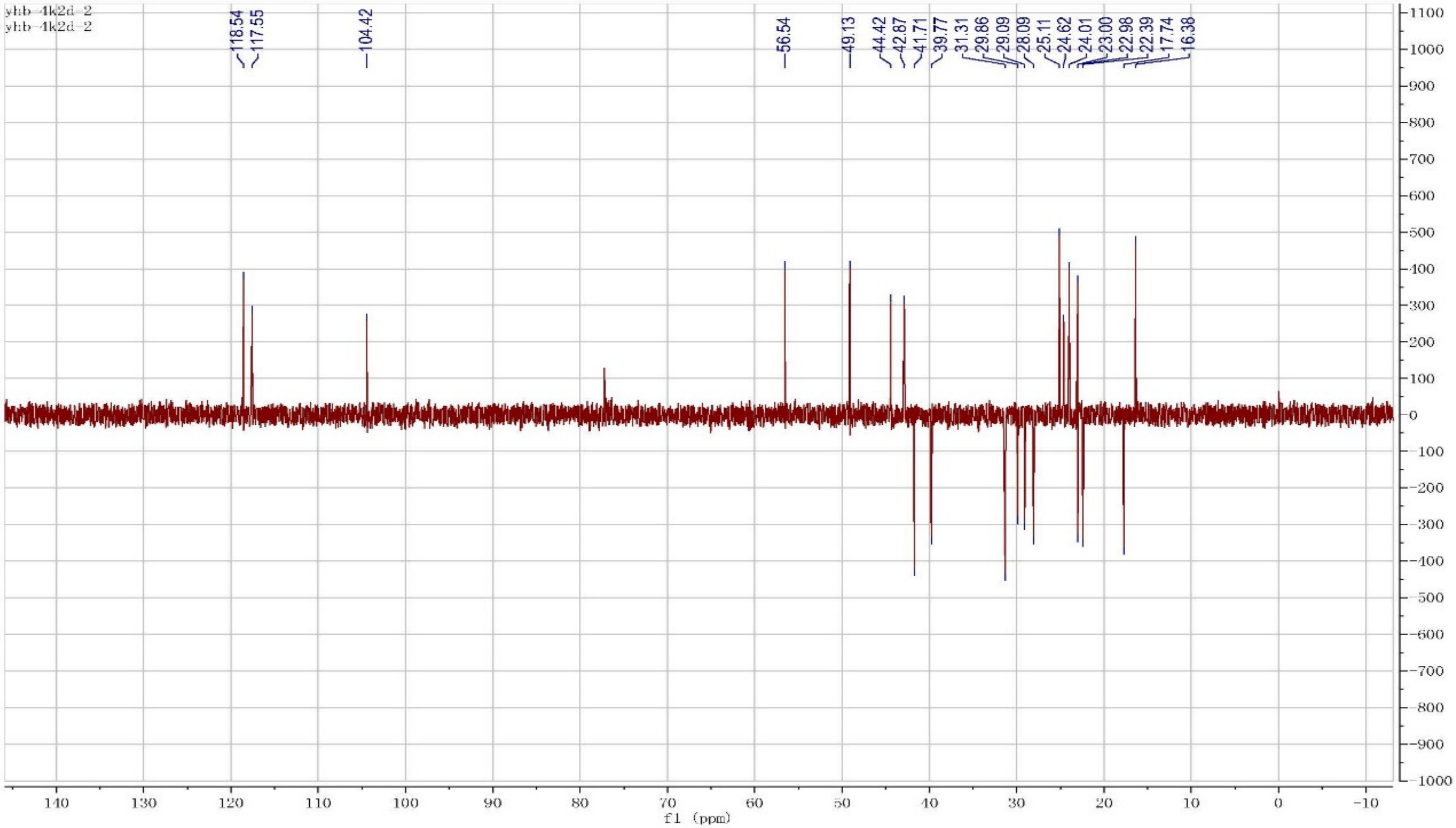

S45 HSQC spectrum of dactylospene E (**5**) in $CDCl_3$.

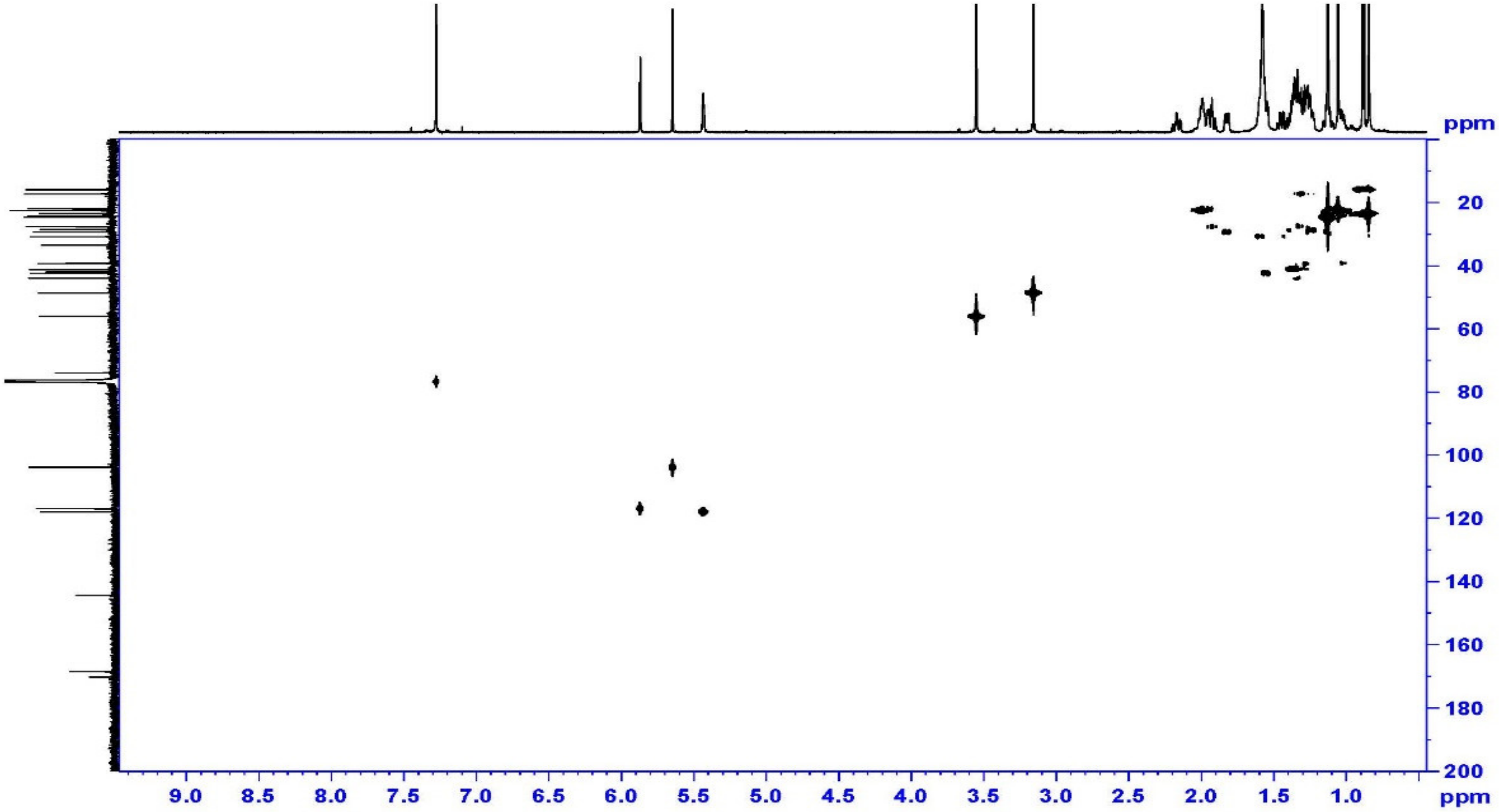

S46 COSY spectrum of dactylospene E (**5**) in $CDCl_3$.

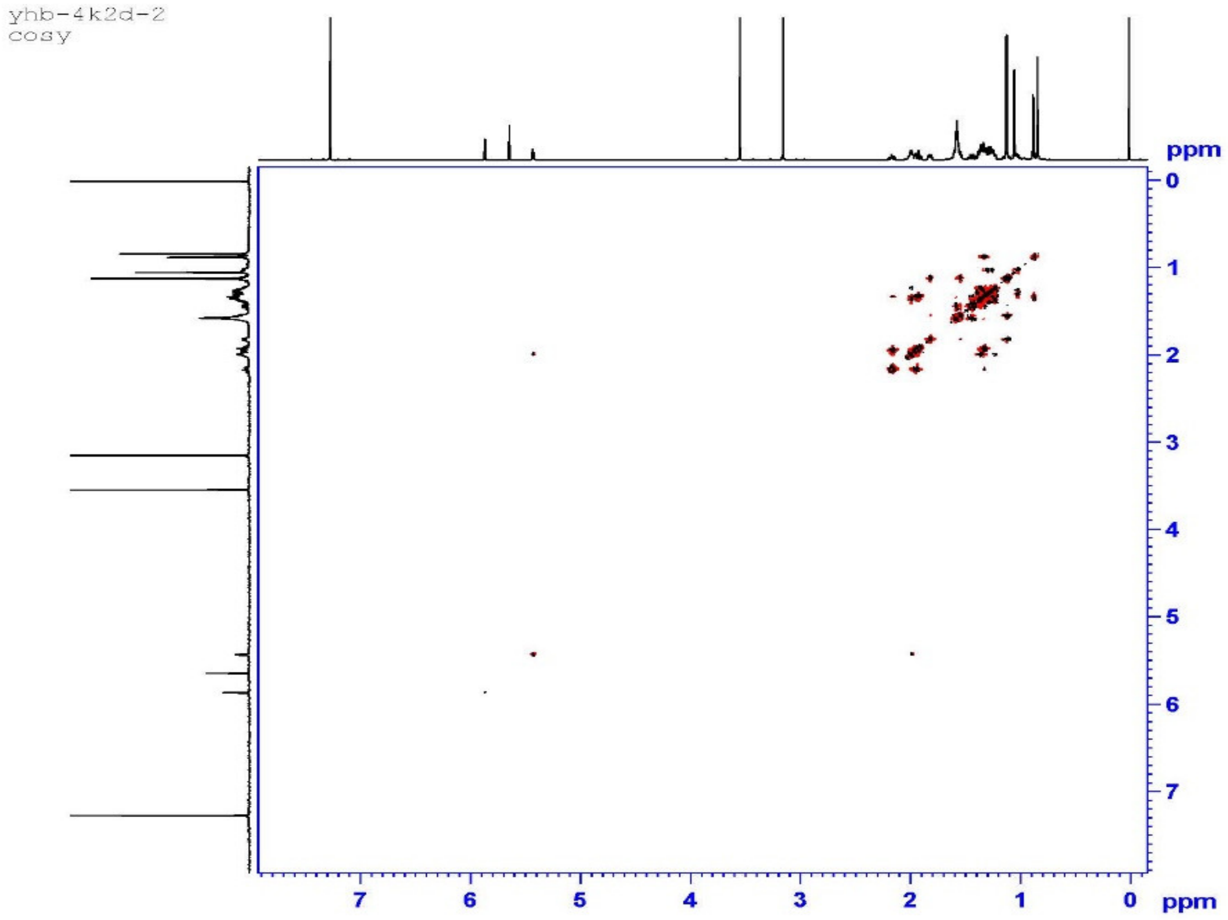

S47 HMBC spectrum of dactylospene E (**5**) in $CDCl_3$.

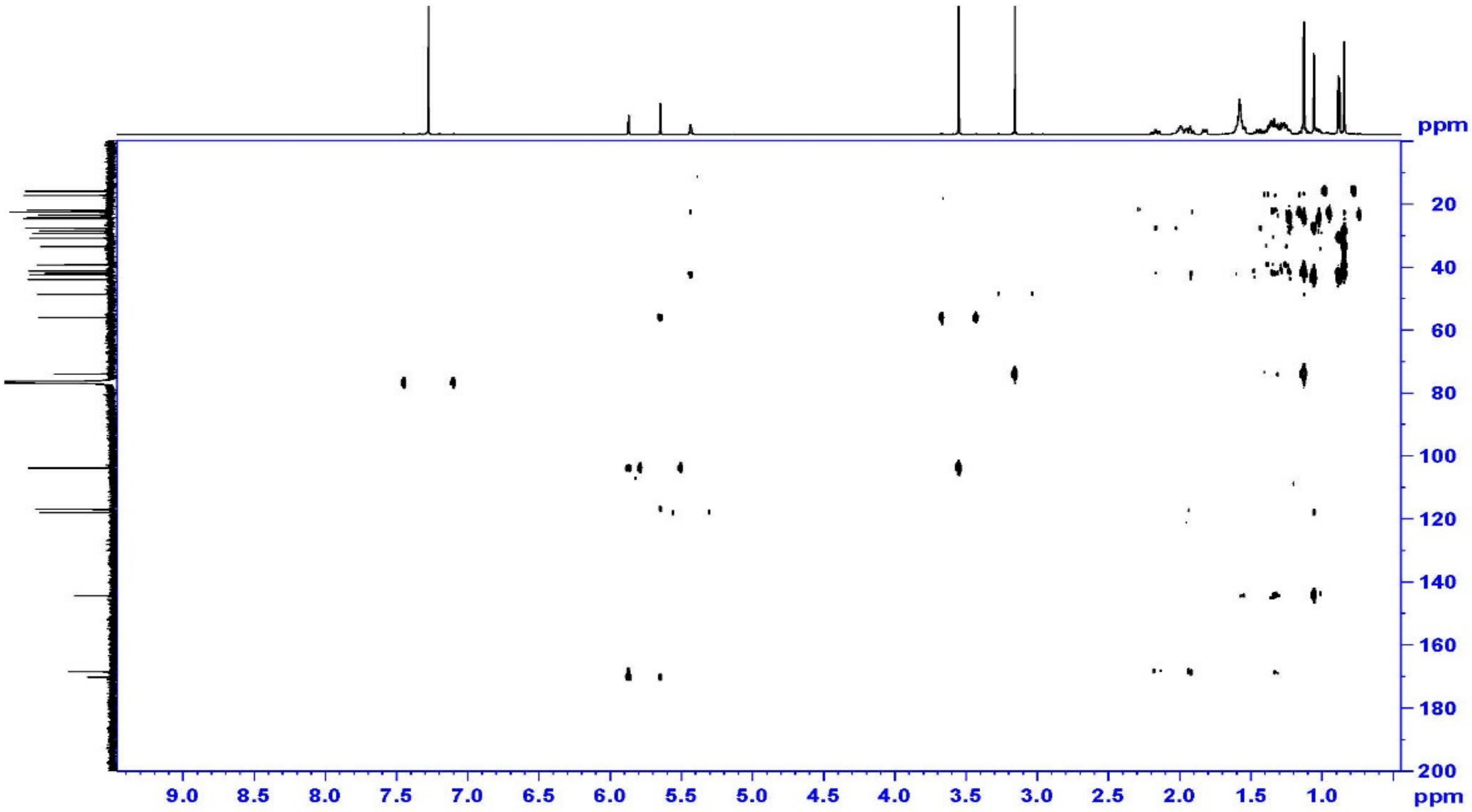

S48 NOESY spectrum of dactylospene E (**5**) in $CDCl_3$.

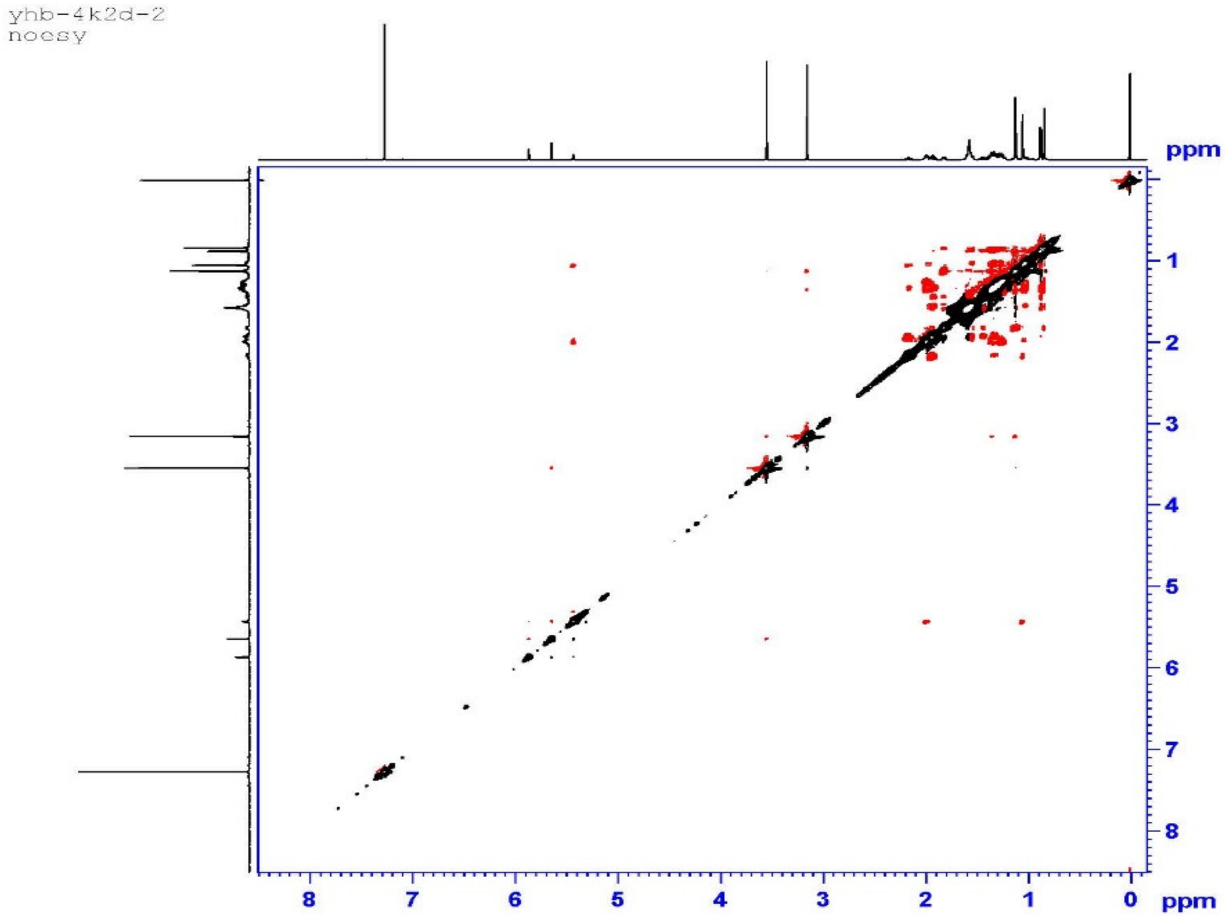

S49 HRESIMS of dactylospene E (**5**).

User Spectra

Fragmentor Voltage	Collision Energy	Ionization Mode
150	0	ESI

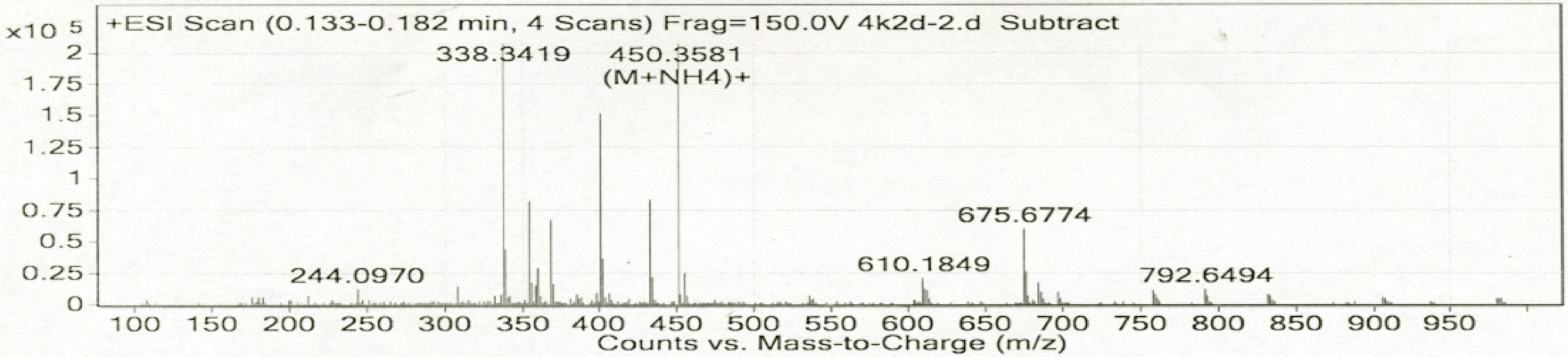

Peak List

m/z	z	Abund	Formula	Ion
338.3419	1	224438.3		
339.3452	1	44001.1		
355.3684	1	82536.3		
369.2792	1	67543.7		
401.3055	1	151442.3		
402.3089	1	36489.6		
433.3316	1	82772.9		
450.3581	1	207072.6	C27 H48 N O4	(M+NH4)+
451.3617	1	50206.2	C27 H48 N O4	(M+NH4)+
675.6774	1	60295.6		

Formula Calculator Results

IonFormula	Measured Mass	Tgt Mass	Diff (ppm)	Score
C27 H48 N O4	450.3581	450.3578	-0.84	94.11

--- End Of Report ---

S50 UV spectrum of dactylospene E (**5**).

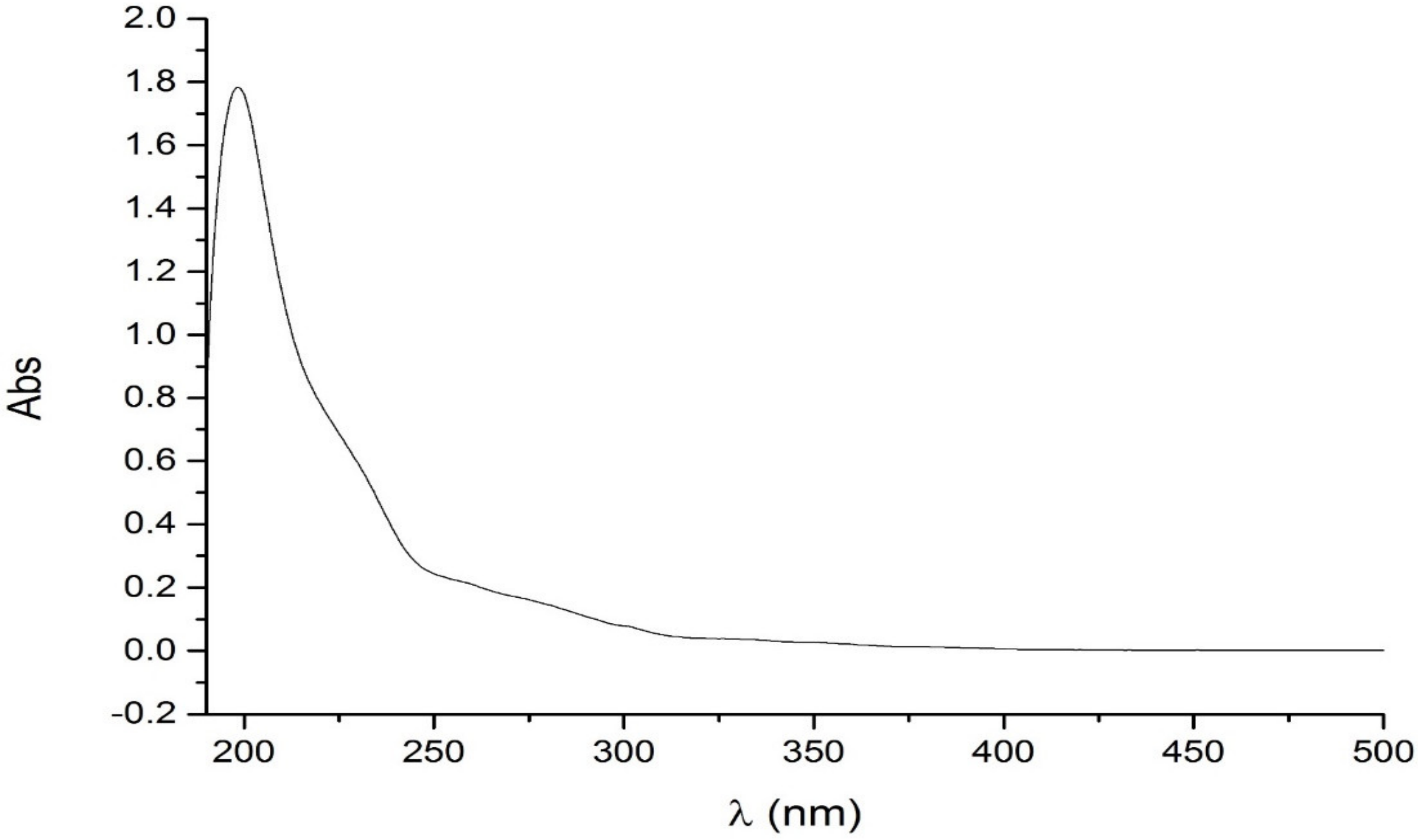

S51 IR spectrum of dactylospene E (**5**).

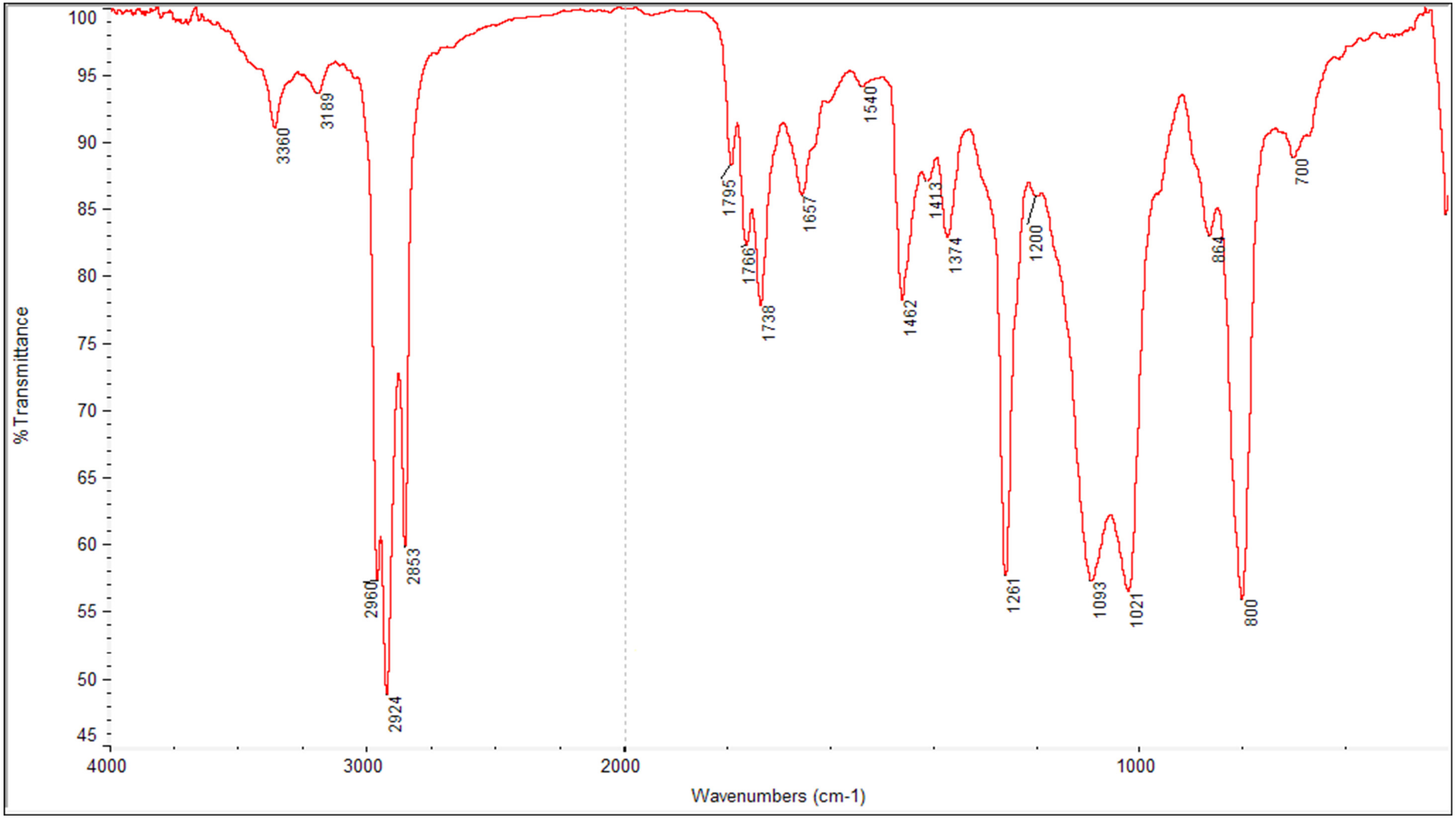

S52 CD spectra of dactylospenes A (**1**).

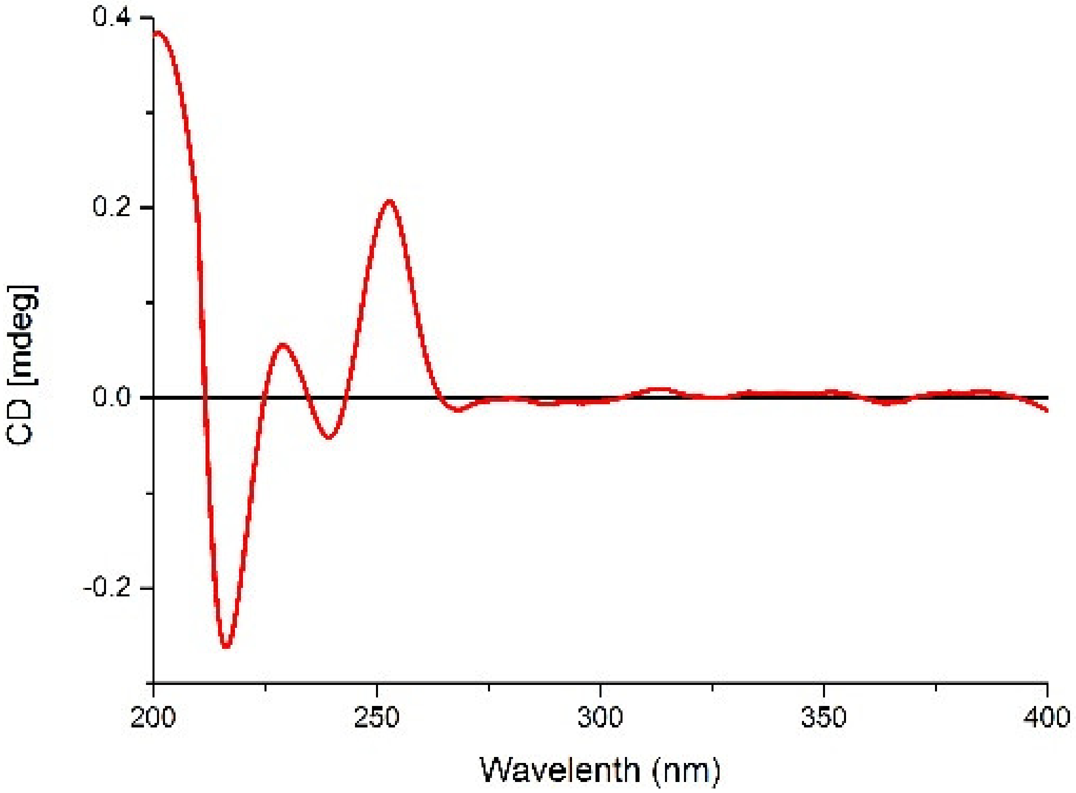

Supplement: Supplementary file 1 [file marinedrugs-18-00491-s001.pdf]
